# What is the volume, diversity and nature of recent, robust evidence for the use of peer support in health and social care? An evidence and gap map

**DOI:** 10.1002/cl2.1264

**Published:** 2022-07-26

**Authors:** Anna Price, Siân de Bell, Naomi Shaw, Alison Bethel, Rob Anderson, Jo Thompson Coon

**Affiliations:** ^1^ Exeter HS&DR Evidence Synthesis Centre University of Exeter Medical School, University of Exeter Exeter UK

## Abstract

**Background:**

Peer support interventions involve people drawing on shared personal experience to help one another improve their physical or mental health, or reduce social isolation. If effective, they may also lessen the demand on health and social care services, reducing costs. However, the design and delivery of peer support varies greatly, from the targeted problem or need, the setting and mode of delivery, to the number and content of sessions. Robust evidence is essential for policymakers commissioning peer support and practitioners delivering services in health care and community settings. This map draws together evidence on different types of peer support to support the design and delivery of interventions.

**Objectives:**

The aim of this map was to provide an overview of the volume, diversity and nature of recent, high quality evidence on the effectiveness and cost‐effectiveness of the use of peer support in health and social care.

**Search Methods:**

We searched MEDLINE, seven further bibliographic databases, and Epistemonikos for systematic reviews (in October 2020), randomised controlled trials (in March 2021) and economic evaluations (in May 2021) on the effectiveness of peer support interventions in health and social care. We also conducted searches of Google Scholar, two trial registers, PROSPERO, and completed citation chasing on included studies.

**Selection Criteria:**

Systematic reviews, randomised controlled trials and economic evaluations were included in the map. Included studies focused on adult populations with a defined health or social care need, were conducted in high‐income countries, and published since 2015. Any measure of effectiveness was included, as was any form of peer support providing the peer had shared experience with the participant and a formalised role.

**Data Collection and Analysis:**

Data were extracted on the type of peer support intervention and outcomes assessed in included studies. Standardised tools were used to assess study quality for all studies: assessing the methodological quality of systematic reviews 2 for systematic reviews; Cochrane risk of bias tool for randomised controlled trials; and consensus health economic criteria list for economic evaluations.

**Main Results:**

We included 91 studies: 32 systematic reviews; 52 randomised controlled trials; and 7 economic evaluations. Whilst most included systematic reviews and economic evaluations were assessed to be of low or medium quality, the majority of randomised controlled trials were of higher quality. There were concentrations of evidence relating to different types of peer support, including education, psychological support, self‐care/self‐management and social support. Populations with long‐term health conditions were most commonly studied. The majority of studies measured health‐related indicators as outcomes; few studies assessed cost‐effectiveness. Studies were unevenly distributed geographically, with most being conducted in the USA. Several gaps were evident regarding the delivery of peer support, particularly the integration of peers and professionals in delivering support and interventions of longer duration.

**Authors' Conclusions:**

Although there is evidence available to inform the commissioning and delivery of peer support in health and social care, there are also clear gaps that need to be addressed to further support provision, particularly regarding cost‐effectiveness. The effectiveness of peer support in different countries, with varying health and social care systems, is a priority for future research, as is the integration of peers with professionals in delivering peer support.

## 1 PLAIN LANGUAGE SUMMARY

### 1.1 Evidence and gap map finds unevenly distributed evidence on effectiveness of using peer support in health and social care

There is evidence related to educational and emotional peer support, and peer support interventions for people with long‐term health conditions, but there are considerable gaps in evidence for peer support in countries and health care systems beyond North America.

### 1.2 What is this evidence and gap map (EGM) about?

Peer support—people drawing on shared personal experience to help one another—can directly benefit individuals, reducing social isolation and mental ill‐health, and potentially improving management of long‐term health conditions. If it increases the effectiveness with which people manage their conditions or address their own needs, it could also lessen demand for health and social care services.

However, peer support varies in its design and delivery, the problems or needs that it seeks to address, the setting in which it takes place, the extent to which it is linked to formal care services, and the number and content of sessions. This variation creates a challenge to those seeking to provide peer support services, such as policymakers and practitioners, in finding and understanding evidence relevant to the type of peer support they are planning to deliver to aid decision making.

**What is the aim of this EGM?**

This EGM is a visual resource presenting recent, high quality evidence on the effectiveness and cost‐effectiveness of the use of peer support in health and social care.

### 1.3 What studies are included?

This EGM includes systematic reviews and impact evaluations (randomised control trials and economic evaluations, if not already included in a systematic review) on the effectiveness and cost‐effectiveness of peer support.

Included studies are published in English, conducted in high‐income countries, and focused on adult populations (aged 18 and over) with a health or social care need.

Any type of peer support was included, as long as peer supporters had the same or a similar health condition as the person they were supporting and had received training, had a contract, or received ongoing support.

The map includes 91 studies: 32 systematic reviews (including 242 impact evaluations) and 59 impact evaluations (52 randomised controlled trials and 7 economic evaluations).

### 1.4 What are the main findings of this EGM?

Studies included in the map varied in quality; the included systematic reviews and economic evaluations tended to be of low or medium quality, whilst randomised controlled trials were generally of higher quality.

The most investigated peer support interventions included education, emotional and wellbeing support, help with self‐care and self‐management, and social support. Few studies, particularly systematic reviews, looked at case management by peers. People with long‐term health conditions were the most frequently studied population.

Studies looked mostly at the effectiveness of peer support in improving health, both physical and mental, as well as wellbeing and social connection. Few studies examined the cost‐effectiveness of peer support. The majority of studies took place in the USA.

On the delivery of peer support, studies tended to focus on in‐person peer support. There was a gap regarding the integration of peers and professionals in delivering support.

The evidence on long‐term peer support was limited, with studies generally focusing on short interventions of up to 3 months or up to 6 months.

### 1.5 What do the findings of the map mean?

This EGM provides information for policymakers and practitioners commissioning or delivering peer support. It also indicates a need for more research on the cost‐effectiveness of peer support, on different ways of delivering it, and in countries beyond the USA.

### 1.6 How up‐to‐date is this EGM?

The authors searched for systematic reviews published from 2015 to 2021 and impact evaluations published up to 2021.

## 2 BACKGROUND

### 2.1 Introduction

#### 2.1.1 The problem, condition or issue

The importance of taking a person‐centred approach to healthcare is increasingly recognised by health and social care services worldwide, with the World Health Organization publishing a global strategy in 2015 (World Health Organization, 2015). Engaging and empowering patients to take a more active role in their own care is particularly important in the context of the increasing rates of non‐communicable diseases (NCDs), along with ageing populations, seen in many countries (Vos et al., 2020). These mean that people are living with health conditions for longer periods of time, placing an increasing burden on healthcare systems (World Health Organization, 2015). Whilst public health has tended to focus on reducing mortality, disability due to NCDs is becoming a greater problem, with a corresponding need to develop interventions to address this issue (Vos et al., 2020). Peer support, which in its simplest form is support exchanged by people who have a shared experience (Mind, 2013), has the potential to be both long‐term and low cost, and is an example of a person‐centred intervention which could support people to manage their own health (Mind, 2013; World Health Organization, 2015).

In the UK, financial pressure on the NHS and social care services is increasing, with the impact of slowed growth and reductions in funding (Health Foundation Kings Fund & Nuffield Trust, 2018) exacerbated both by increased demand from an aging population and the consequences of Covid‐19 (Anderson et al., 2021; NHS Digital, 2016). There was an estimated annual funding deficit of almost £30 billion within the NHS in 2020/2021 (NHS England, 2014), with a minimum annual increase of 4% recommended to maintain and improve services (Anderson et al., 2021). Social care organisations have sought to reduce the impact of reductions in funding and increasing costs associated with complex care (Cromarty et al., 2019). Yet despite an injection of ring‐fenced funding totalling £10 billion between 2017/2018 and 2019/2020 (Local Government Association, 2017), it is estimated that an annual funding increase of at least £3.9 billion is needed by 2023/24 to meet current funding shortfalls in social care (House of Commons Health and Social Care Committee, 2020). Part of the policy response to these pressures is increased focus on enabling patients and carers to support themselves more effectively (Anderson et al., 2021).

The shift in policy and practice towards more patient‐centred and person‐centred care over the last few decades, within the UK and more widely, recognises the capabilities and knowledge of people who manage their conditions themselves and the need to connect with others who have similar problems or care needs (Wilson et al., 2007). The NHS Long Term Plan (for England) outlines an intention to empower patients through increasing their involvement in their own care, focusing on patients' own health and wellbeing goals, improved access to information, and peer support within the community (NHS England, 2019a). This is increasingly important given the prevalence of long‐term, often co‐occurring, conditions including diabetes and heart disease in the population, and the need to enable and empower people to self‐manage associated lifestyle risk factors.

Whilst the use of peer support‐based initiatives is currently increasing in the UK, their emergence to complement or substitute for formal, professionally delivered services has a longer history. An early example, launched by the UK Department of Health in 2002, is the Expert Patients Programme, a 6‐week course for people with long‐term health conditions which was adapted from the Stanford Chronic Disease Self‐Management Programme (Griffiths et al., 2007; Wilson et al., 2007). In mental healthcare, research suggests the ‘recovery movement’ and recovery colleges—groups of people with shared experience of living with mental health problems who help each other understand and care for themselves—can aid recovery (Meddings et al., 2015), including reducing service use (Bourne et al., 2018).

Globally, peer support interventions have been used in a range of populations, to address mental health difficulties (Cabassa et al., 2017; Lyons et al., 2021), physical health problems including HIV (Kanters et al., 2016) and cancer (Hu et al., 2019; Lee & Suh, 2018), as well as other health and social care needs such as those of carers (Dam et al., 2016), or new mothers (Leger & Letourneau, 2015). An early example is the Stanford Chronic Disease Self‐Management Programme, which was developed in the USA in 1979, originally using lay people to teach self‐management for arthritis before being adapted for chronic diseases more widely (Griffiths et al., 2007).

#### 2.1.2 The intervention

The potential to make use of the ‘renewable energy’ of the individuals and networks available within the wider community has been recognised within formal services for people with long‐term physical conditions such as HIV and diabetes (Health Foundation & Nesta, 2016), with guidelines and research to support the development of peer support initiatives also being developed for other population groups, including people living with dementia (Health Innovation Network, 2015), those experiencing mental health difficulties (NHS England, 2019b; World Health Organization, 2017), and women requiring peri and/or postnatal support (Hetherington et al., 2015; Woodman & NHS Health Scotland, 2013).

##### Defining peer support

A consistent factor in definitions of peer support is the importance of peer supporters having experience of the same health condition or difficulty as the people they are supporting. This may be either because they have the same problem, have previously had it, or have cared for someone who has it. Whilst there are range of definitions, all appear broadly consistent with the 2015 report published by Nesta and National Voices, on ‘Peer support: what is it and does it work?’, which defines peer support as:

…people drawing on shared personal experience to provide knowledge, social interaction, emotional assistance or practical help to each other, often in a way that is mutually beneficial (Nesta & National Voices, 2015).

This definition provides a conceptual explanation of peer support but in terms of practical delivery there are a huge variety of peer support interventions (Nesta & National Voices, 2015). Dennis outlines how the different ways people can access peer support lie upon a continuum (Dennis, 2003), with interventions varying in:

– Delivery formats, which include face‐to‐face groups or one‐to‐one contact, online forums, telephone, and email (Mind, 2013; Valenstein et al., 2016). Peer support may also be uni‐directional (one peer provides support but does not receive it) or bidirectional (both peers provide and receive support) (Valenstein et al., 2016).
– Content, including the degree of structure (Valenstein et al., 2016), with interventions focusing on providing information and education, emotional support or a combination (Nesta & National Voices, 2015). Differences are a result of the needs of the population, structure of existing services, resources available and intended outcomes.
– Support from, or association with, formal health and social care services. Peer supporters can be paid employees recruited by health care organisations or third sector agencies, or volunteer workers.

##### How the intervention might work

Qualitative evidence provides some insight into the underlying mechanisms or processes which may underpin the effectiveness of peer support interventions. In their scoping review synthesising evidence regarding the processes or mechanisms underlying one‐to‐one peer support for adults using mental health services, Watson identified five mechanisms: ‘lived experience, love labour, the liminal position of the peer worker, strengths‐focussed social and practical support, and the helper role’ (Watson, 2019). See Box 1.

Box 1 **Mechanisms underpinning peer support (Watson, 2019)**

1 Lived experience—peer supporters use their lived experience of a health condition or difficulty in two different ways. By explicitly sharing their experiences, they build connections and credibility with peers therefore promoting hope, whilst implicitly, their own experiences influence their approach to providing peer support.

Love labour—whilst it can be challenging for the peer supporter, a strong emotional connection between peer supporter and peer enhances the effectiveness of peer support, supporting the wellbeing and recovery of peer and peer supporter.

The liminal position of the peer worker—peer supporters are neither professionals or service users, so can model recovery to both, as well as facilitating communication between them.

Strengths‐focussed social and practical support—peer supporters provide social and practical support which can help peers in their daily lives.

The helper role—peer supporters are benefited through their role as a helper, from feeling useful to gaining a sense of achievement and competence.

The importance of lived experience is also highlighted by Bailie and colleagues, in a study of users of peer support which focused on the core role of the relationship between the professional peer support worker and service user (Bailie et al., 2016). The study emphasised how a shared sense of identity can be developed through disclosure of past experiences, deepening the relationship and helping the service user feel understood. Peer supporters also bring perspectives relating to shared sociodemographic characteristics such as gender and ethnicity (National Collaborating Centre for Mental Health, UCLPartners, Care City Health Education England, & PPL Consulting, 2020); an understanding of the cultural background of the potential participants of proposed peer support may be beneficial in designing interventions that work (Jamison et al., 2017; Mayer et al., 2019).

Peer support‐based interventions and initiatives seek to tackle different problems and needs, and are therefore designed and delivered in different ways. Accordingly, we should expect variation between interventions in the outcomes achieved and likely mechanisms underlying these. Outcomes resulting from peer support interventions range from direct benefits to the health of individuals, both physical and mental (Health Foundation & Nesta, 2016; Ramchand et al., 2017), to intermediate outcomes, such as empowerment, improved self‐management, or social inclusion (Mind, 2013), as well as increased knowledge about a health condition (Ramchand et al., 2017). For example, reviews of peer support for Type 2 diabetes have found reductions in blood glucose and increased diabetes knowledge as a result of peer support interventions (Gatlin et al., 2017; Krishnamoorthy et al., 2019). Additionally, some peer support programmes have resulted in reduced use of health services (Campos et al., 2016; Mind, 2013).

It should be noted, that in some cases, interventions may have no effect, or even negative impacts (Burke et al., 2019; Chien et al., 2019). One critique of peer support programs, as with other health interventions, is that they may fail to recruit those most in need (Sokol & Fisher, 2016; Wilson et al., 2007). Other criticisms can be considered in relation to the mechanisms described above, which can have negative as well as positive impacts (Watson, 2019). Participants may not benefit as they compare themselves negatively to peer supporters, whilst peer supporters might be affected by revisiting past experiences, or the emotional or practical burdens of providing support (Embuldeniya et al., 2013; Watson, 2019).

##### Existing evidence: Systematic reviews and grey literature

Background scoping searches found a range of evidence on the effectiveness of peer support in specific populations published in peer‐reviewed academic journals. These populations include people living with HIV (Kanters et al., 2016), breast cancer (Hu et al., 2019), or mental health difficulties (Campos et al., 2016); caring for someone with dementia (Dam et al., 2016), or in the critical care setting (Haines et al., 2018). Broader summaries of peer support, across populations, or considering content or mode of delivery, are limited. Whilst there are no existing EGMs on the effectiveness of using peer support in health and social, there are two systematic reviews which focus on peer support for health promotion and disease prevention and peer support for ‘hardly reached’ populations, both are restricted to evidence from the USA (Ramchand et al., 2017; Sokol & Fisher, 2016).

However, reports have been published in the grey literature in recent years. Two key reports from national care/policy organisations in the UK were published in 2015, by National Voices and Nesta, and in 2013, by the mental health charity Mind (Mind, 2013; Nesta & National Voices, 2015). Both looked at the range of peer support interventions available and the different ways they are described, with Mind also collating the experiences of peer support groups with a view to supporting the development of emerging peer support projects. National Voices and Nesta (2015) concluded that it was not possible to identify which method of peer support delivery was most effective, as the majority of studies identified did not compare different modes of delivery to one another. Furthermore, evidence pertaining to the cost‐effectiveness of peer support interventions was inconclusive. Mind highlighted several key issues, including the lack of evidence regarding use of peer support in minority and marginalised communities, the sustainability of funding, the need for adequate training, support and supervision for those delivering peer interventions and whether management of peer support interventions is overseen by services or devolved to service users.

Whilst these reports highlight the variety and broadly perceived value of peer support interventions being implemented across multiple populations and settings, the usefulness of this study to inform the commissioning or delivery of peer support in particular contexts or populations is less certain.

#### 2.1.3 Why it is important to develop the EGM

Peer support can directly benefit participants, addressing a range of health and social needs, including social isolation (Jones et al., 2014) and mental ill‐health (Repper & Carter, 2010). It is an inherently person‐centred form of care, which also has the potential to reduce demand on services through improved condition management (NHS England & Local Government Association, 2017) and reduced emergency admissions (Deeny et al., 2018). There has been a desire to professionalise peer supporters and maintain standards through increased accreditation and training. This creates a dilemma, as it may conflict with the wish to maintain the ‘authenticity’ of peer support services by their separation from formal services (National Collaborating Centre for Mental Health et al., 2020; Q LAB, 2018). Whilst this is beyond the scope of this EGM to address, our focus on the effectiveness of peer support by peer supporters with a formal role is important, as this form of provision is increasing.

By presenting an overview of the evidence in an accessible format, this EGM will allow those designing and providing services, to find studies on the effectiveness and cost‐effectiveness of peer support, as well as rapidly discover where evidence of effectiveness is currently lacking. The decision to produce an EGM, rather than a detailed synthesis of the effectiveness of a current uncertainty, was guided by stakeholders, with the ability to find evidence to inform ongoing decision‐making felt to be most helpful in meeting the needs of multiple stakeholders. As peer support interventions vary in terms of delivery, content and underlying actual or intended mechanisms, this presents a challenge to commissioners and service providers as these models may differ in effectiveness, particularly depending on the circumstances in which they are used (Valenstein et al., 2016). Being able to access the spread of evidence relating to specific populations and for specific peer support interventions, or features of these interventions, and determine whether or not they are effective, will aid decision‐makers in providing appropriate interventions to achieve their aims.

Conversely, it is also important to see for what health and social needs, or what models of delivery of peer support, there has been no or little research (i.e. the ‘gaps’ in the evidence map). If these evidence gaps relate to needs and types of peer support where there are peer support initiatives being developed and delivered, then this may help research funders prioritise where high quality evaluations are currently needed. Similarly, if there is a growing body of primary research on some types of peer support intervention, but none of it has yet been synthesised as part of a systematic review, or very little of it is high quality, this may also enable more targeted research funding.

## 3 OBJECTIVES

Our aim was to identify and appraise the volume, diversity and nature of recent, high quality evidence (systematic reviews, randomised controlled trials and economic evaluations published after 2015) for the use of peer support in health and social care. Nature refers to the information contained within each individual review e.g. the study population, whereas diversity refers to the variation, in terms of content and focus, between reviews.

Specific research objectives were to:

– Map the recent, robust evidence for the effectiveness of peer support interventions across health and social care.
– Map the recent, robust evidence for the cost‐effectiveness of peer support interventions across health and social care.

## 4 METHODS

### 4.1 Evidence and gap map: Definition and purpose

EGMs provide an overview of the evidence in a given area. They are produced using the same principles as systematic reviews but give a visual presentation of the types and focus of existing studies available rather than a synthesis of findings (Snilstveit et al., 2013; White et al., 2020).

This EGM is presented in two dimensions as a table, with subcategories associated with both dimensions (Saran & White, 2018). The rows typically list types of interventions (i.e. different ways of delivering peer support) and the columns different outcomes; each cell shows the number of studies containing evidence on that particular combination of intervention and outcome. Within cells, studies are separated by type (systematic reviews, and randomised controlled trials and economic evaluations) and their assessed quality.

By displaying the volume, diversity and key characteristics of existing evaluations (including systematic reviews), EGMs allow users to identify and locate the research evidence (or evidence gaps) relevant to their patient or intervention focus. They can therefore support evidence‐informed policy, commissioning, and provision, or prioritise the focus of future research (Snilstveit et al., 2013).

### 4.2 Framework development and scope

The scope of this EGM is to capture recent evidence on the effectiveness and cost‐effectiveness of peer support in health and social care. Recent was defined as evidence published after 2015, with this date chosen to focus on evaluations not included in other comprehensive reviews of evidence relating to peer support (Mind, 2013; Nesta & National Voices, 2015).

Framework development was informed by previous research (Nesta & National Voices, 2015), which was used to define key terms and create a list of important categories. This was refined itteratively, through consultation with and input from Fiona Campbell (University of Sheffield), who is experienced in producing Evidence and Gap Maps, and stakeholder engagement as detailed below. Categories were designed to be accessible and usable on the map.

Where categories were identified in the included research that did not fit the Framework, categories were renamed, or their scope adjusted to ensure that all reported population characteristics, and outcomes relating to effectiveness and cost‐effectiveness, were included in the EGM. This adjustment was discussed in group meetings, to ensure consistency, and resolved in consultation with stakeholders, where appropriate.

For a list and description of intervention and outcome categories included in the Framework, please see the EGM Glossary, included below as Supporting Information: Appendix 1.

### 4.3 Stakeholder engagement

User involvement in evidence synthesis is important to ensure useful outputs from the review process (Konnerup & Sowden, 2008). Stakeholders consulted in the production of this EGM included policymakers, health care professionals, academics, third sector organisations providing peer support, and online providers of peer support resources and training, from the following organisations:

- Central & North West London NHS Foundation Trust
- Mind
- National Perinatal Mental Health, NHS England
- Prostate Cancer UK
- Q Lab
- With‐you
- University College London

We also recruited users of peer support to form a public patient involvement (PPI) group. Whilst not the primary intended audience of the EGM, we felt it was important to involve users of peer support for the insight their lived experience could provide on improving and extending use of the map (Gierisch et al., 2019). All stakeholders were identified through word‐of‐mouth and snowballing techniques and invited to workshops and individual meetings, to suit project progress and stakeholder availability.

We consulted stakeholders throughout the review, with early conversations focused on defining and developing the scope of our research questions and protocol, and later consultation focusing on production of the EGM (Haddaway et al., 2017; Konnerup & Sowden, 2008). We also consulted stakeholders on aspects of the report such as the plain language summaries describing our review and its findings, to ensure they are accessible to a range of audiences.

Initial consultations helped to identify key populations and outcome categories within our EGM as well as focus the definition of peer support. Once a prototype version of the EGM had been developed and populated with some study data, later trials of the use of the map in workshops with stakeholders resulted in discussion of the language used in the EGM. We incorporated feedback from all stages of consultation to ensure the final EGM provides a level of information useful to the intended audiences. Table 1 details specific points of feedback and decisions regarding these.

**Table 1:** Changes made to the EGM as a result of stakeholder consultation

Type of change	Comment	Actions and response
Definitions and language	Having a narrow medicalised definition of peer support as an intervention could be considered problematic. Peer support is a process and not something that is done to people. The word ‘intervention’ was also removed from the Health Education England Peer Support Worker Framework (National Collaborating Centre for Mental Health et al., 2020).	Discussed more helpful terms and how these depend on the audience; consulted with additional stakeholders to ensure all views were represented.
		As the main audience for the EGM are service providers and the term intervention is familiar to them, we decided to keep this language in the EGM but add context to glossary and report.
	Measuring outcomes could also be considered problematic for reasons outlined above.	We are limited by the EGM aims, which are to map evidence of effectiveness, but have reflected and contextualised this in the report to acknowledge what is being missed in this EGM.
	Definition of community based/community delivered, these can be interpreted differently. For example, peer support could be delivered in a community venue, delivered by a community practitioner, or a community‐based peer support group. All may fall in the same category at present but are very different.	We are limited by the information provided by included studies, currently these distinctions are not clearly or consistently defined in the literature, so we are unable to include them in the EGM.
	Clarify the ‘People at risk’ heading and definition: need to be mindful of language and judgement.	We discussed, and consulted on, alternative language and decided to change this category to ‘Vulnerable’.
Map presentation	Difficult to distinguish between SRs and RCTs when first looking at the map. The donut rather than bubble style of presenting studies in the cells might represent the study types more clearly.	RCTs and SRs have been clearly defined in the glossary/definitions section of the map; we have written out the terms in full on the map rather than using abbreviations for clarity.
		It is not possible to pre‐programme the map to start with the donut style, but we have provided map instructions so that people know how to change the style when viewing.
	Filters for countries would be useful as evidence from different countries may have different contextual relevance.	Added country filter to map, with a focus on distinguishing between UK and non‐UK studies, as well as studies with potentially similar health systems.
	Include filter for year.	Communicated more clearly in the EGM description that only research from 2015 onwards is included and added a filter for year to the EGM.
	The indication of quality of evidence in the EGM could be more intuitive. A general description might work better than risk of bias and quality appraisal.	Discussion about what might work, particularly in terms of alternative colours.
		Changed wording to refer to ‘quality’ and used broad categories within the EGM. Kept data on specific quality appraisal tool (AMSTAR2 and ROB) in filters on map with full detail in report.
	It may be helpful to filter the peer support provider, e.g., NHS/non NHS.	This data is not generally available in SRs and is only available in a limited number of RCTs. We have coded for ‘who facilitates the intervention’ to indicate the degree of professional involvement in peer support provision.
Useability	The filtering aspect of the map needs more explanation.	Information on how to ‘select filtering mode’ under filtering added to the map instructions.
	Having ‘Segment’ as the default when clicking on the map would indicate which records are SRs and RCTs more clearly.	This is not currently an option when creating the map. Information on how to choose the ‘Segment’ option has been added to the map instructions.
	It would be useful to be able to find studies on specific health conditions in the map.	There is a ‘search’ function available in the map, this has been indicated in the map instructions.
	Find a way to update the map, this is important in such a fast moving field.	The team has discussed putting together a funding application. This could, for example, be to update the map every 6 months and to work on developing the methodology.

Abbreviations: AMSTAR2, assessing the methodological quality of systematic reviews; EGM, evidence and gap map; RCT, randomised controlled trial; ROB, Cochrane risk of bias tool.

### 4.4 Conceptual framework

Peer support is defined by the shared lived experience of peers and peer supporters and this is also the key mechanism in its effectiveness [Box 1; (Watson, 2019)]. However, previous reviews of evidence relating to peer support indicate diversity in the forms of peer support available and that people are therefore experiencing (Dennis, 2003). Effectivness may vary depending on the type of support (Mind, 2013; Nesta & National Voices, 2015).

Key categories through which peer support interventions can be described and which may influence its effectiveness are (Nesta & National Voices, 2015):

- Who—the people involved, the health condition(s) they are experiencing, and their sociodemographic characteristics;
- What—the focus and type of support offered, whether education, emotional or practical, and who facilitates it;
- How—whether meetings are in person or online, occur individually or in groups, and the support offered to peer supporters;
- Where—the location of its provision, for example, healthcare or community settings;
- When—the duration of the intervention.

### 4.5 Dimensions

#### 4.5.1 Types of study design

Impact evaluations study change in an outcome which can be attributed to an intervention (3ie, 2012). This EGM includes:

- Systematic reviews (SRs) of impact evaluations;
  and two types of impact evaluation:
- Randomised controlled trials (RCTs);
- Economic evaluations (EEs e.g. comparative costing studies, cost‐effectiveness studies).

SRs can be defined as studies which collect and synthesise all the research available on a topic to answer a specific question (Chandler et al., 2022). They typically seek to minimise bias by specifying their methods in advance, and assessing the quality of included studies using standard critical appraisal tools. RCTs are experimental studies intended primarily to assess the effectiveness of alternative interventions, in which people are randomly allocated to different groups, and each group receives a different intervention [Cochrane Effective Practice and Organisation of Care (EPOC), 2017]. EEs are studies which simultaneously compare the effectiveness or benefits of alternative inteventions, alongside their resource use and costs (Goodacre & McCabe, 2002).

We included high‐quality SRs of impact evaluations focused on quantitative evidence because they summarise evidence on the effectiveness of peer support in health and social care. We considered SRs for inclusion where they reported a research question, a reproducible search strategy, inclusion/exclusion criteria, screening methods, quality assessment of included studies, and a reproducible method of data analysis (Krnic Martinic et al., 2019).

We included RCTs that were not in the SRs already included in the map because they provide evidence on the effectiveness of peer support in health and social care. We also included EEs, to map evidence relating to the cost‐effectiveness of peer support‐based interventions.

Both published and ongoing studies were screened for inclusion. Detailed eligibility criteria are given in Supporting Information: Appendix 2.

#### 4.5.2 Types of intervention/problem

Interventions involved delivery of peer support as defined in the Background with additional criteria identified through consultation with stakeholders. Peers had to have experience of the same, or a similar health, condition as intervention users. We excluded interventions not meeting this definition of peer support, for example where ‘peers’ were only peers by virtue of being from the same community, or had experience of caring for a person with a condition, but did not have lived experience of the same health condition. In cases where there were a mix of peers, we included the study if the majority of peers met our definition.

Peer support could be delivered in any format (such as face‐to‐face, online, group, individual, mixed modes, etc.) and with any content, delivered by paid or unpaid peer supporters. However, peers had to have a formalised and ongoing role. This was evidenced by one or more of the following: they have received training to fulfil the peer support role; they receive ongoing support to fulfil the peer support role; they are paid or have a contract to fulfil the peer support role.

#### 4.5.3 Types of population

The populations of interest for this EGM were users (or potential users) of adult services with a defined health and/or social care need. Adults were considered to be 18 years or older; where studies focused on young people they were included if the majority of the sample was over 18. For studies focused on populations where only a subset had a health condition, they were included if the effect of peer support on this subset could be distinguished.

We excluded populations identified as ‘at risk’ or engaging in ‘risky behaviour’ unless they had a current defined health or social care need, and were using services. Further examples of population criteria are given in Supporting Information: Appendix 2.

#### 4.5.4 Types of outcome measures

All reported outcomes relating to effectiveness and cost‐effectiveness were of interest. This included outcomes that may not benefit the participant but do benefit the health of others, for example, transmission risk behaviour among HIV‐positive participants. Acceptability outcomes, including feedback on an intervention, were excluded, as they relate to the effectiveness of the intervention rather than of peer support.

#### 4.5.5 Other eligibility criteria

##### Types of location

We included only studies conducted within high‐income countries as defined and listed by the World Bank (as of 27 September 2021) (World Bank, 2021). This was because the funders of this map are working within a healthcare system in a high‐income country, and this restriction ensured the map contained the most relevant information for them.

##### Types of settings

The EGM included all settings where peer support interventions are provided. These might be home, community, online/virtually, health or social care settings.

Studies were only excluded based on setting if additional support was provided to participants in this setting which meant that the effectiveness of the peer support component of the intervention could not be separately distinguished, for example, Alcoholics Anonymous (AA) recovery homes.

### 4.6 Search methods and sources

As described in the EGM protocol (Shaw et al., 2021), studies were identified in two stages:

1. Stage 1 searched for SRs of peer support interventions published from 2015 to October 2020.
2. Stage 2 searched for RCTs and EEs of peer support interventions not included in recent, high quality SRs.

#### 4.6.1 Stage 1 search methods

We searched the following bibliographic databases between 1st and 5th October 2020: Cochrane Database of Systematic Reviews (via the Cochrane Library), CINAHL Complete (via EBSCOhost), Embase, MEDLINE including In‐Process & Other Non‐Indexed Citations, APA PsycINFO (via Ovid), ASSIA (via ProQuest), Epistemonikos (https://www.epistemonikos.org), and ProQuest Dissertations & Theses. The database searches were restricted to SRs published from January 2015 to the search date in October 2020. No language restrictions were applied (Box 2).

Box 2 **Ovid MEDLINE search strategy**

1

**Table:** 

1 (peer* adj3 (administer* or adviser* or advisor* or advocate* or coach* or co‐facilitat* or cofacilitat* or consultant* or counsel* or deliver* or educator* or expert* or facilitator* or group* or helper* or instructor* or leader* or led or listener* or mentor* or navigator* or network* or program* or provider* or specialist* or support* or trainer* or trained or tutor* or worker*)).tw. (17015)	11 (“support network*” or “mutual aid” or “mutual support”).tw. (4051)
	12 (expert adj patient*).tw. (262)
	13 “shared experience”.tw. (365)
	14 *Self‐Help Groups/(5250)
	15 or/1‐14 (54295) [peer support search terms]
	16 ((systematic* or systematized or integrative or mapping or rapid or scoping) adj3 review*).tw. (207271)
2 (“peer‐based” or “peer based”).tw. (423)	17 ((evidence or interpretive or meta or quantitative) adj1 synthes?s).tw. (7427)
3 “peer to peer”.tw. (1394)	18 ((evidence adj2 map) or “systematic map”).tw. (443)
4 peer group/(20749)	19 (“mixed method*” adj3 review*).tw. (572)
5 (buddy or buddies or befriend*).tw. (1078)	20 (“meta‐analys?s” or metaanalys?s).tw. (179152)
6 (“service user*” adj1 (involv* or led or run)).tw. (381)	21 systematic review.pt. (135796)
7 (consumer* adj (deliver* or provider* or led or run)).tw. (329)	22 meta‐analysis.pt. (120248)
8 ((lay or voluntary or volunteer) adj2 (adviser* or advisor* or advocate* or coach* or consultant* or counsel* or educator* or expert* or facilitator* or helper* or instructor* or leader* or led or listener* or mentor* or provider* or specialist* or support* or trainer* or trained or tutor* or worker*)).tw. (5047)	23 (cost* adj3 review*).tw. (3218)
	24 (data adj extraction).ab. (22169)
	25 (narrative adj (review* or synthes?s)).tw. (14659)
9 “lay health care worker*”.ti, ab. (25)	26 or/16‐25 (348857) [systematic review search]
10 ((“social support” adj5 intervention*) or “support group*”).tw. (8568)	27 15 and 26 (1851)
	28 limit 27 to yr=“2015 ‐Current” (1123)

The bibliographic database search strategies were developed using MEDLINE by a team of information specialists (SB/NS/AB) in consultation with the review team. They included both relevant controlled vocabulary, if available (e.g., MeSH in MEDLINE), and free‐text search terms for the concept of peer support with search terms for SRs.

In January 2021 we searched Google Scholar (via Publish or Perish; Harzing), OpenGrey (www.opengrey.eu) and BL Explore (http://explore.bl.uk) and in July 2021 we conducted searches on PROSPERO (https://www.crd.york.ac.uk/prospero) to identify any relevant SR protocols.

#### 4.6.2 Stage 2 search methods

We searched the following bibliographic databases for RCTs between 15 and 17 March 2021: MEDLINE including In‐Process & Other Non‐Indexed Citations, APA PsycINFO (via Ovid), Cochrane Central Register of Controlled Trials (CENTRAL) (via the Cochrane Library), CINAHL Complete (via EBSCOhost). For EEs (searches completed 13th May 2021), we searched the following databases: MEDLINE including In‐Process & Other Non‐Indexed Citations, EMBASE (via Ovid), NHS Economic Evaluation Database (via the Centre for Reviews and Dissemination https://www.crd.york.ac.uk/CRDWeb), HTA database (via the Centre for Reviews and Dissemination https://www.crd.york.ac.uk/CRDWeb), and INAHTA HTA database (http://database.inahta.org).

The search strategies for Stage 2 were developed and tested using the primary articles extracted from included SRs identified in Stage 1, we used MEDLINE for the search development. We combined peer support terms, both free text and controlled vocabulary, with the Cochrane filter for identifying RCTs in MEDLINE (sensitivity and precision maximising 2008 version) (Lefebvre et al., 2021) and the CADTH narrow economic filter (CADTH, 2021). The MEDLINE search strategy was then adapted for each database (Box 3).

Box 3 **Ovid MEDLINE search strategy**

1

**Table:** 

1 randomized controlled trial.pt. (524960)	36 (peer* adj1 run*).ab. (53)
2 controlled clinical trial.pt. (94095)	37 (lay adj2 led). tw. (92)
3 randomi?ed.ab. (613218)	38 “mutual support”.tw. (671)
4 placebo.ab. (216056)	39 (expert adj patient*).tw. (270)
5 exp Clinical Trials as Topic/(353812)	40 (peer* adj2 administer*).tw. (53)
6 randomly.ab. (353046)	41 (peer* adj2 adviser*).tw. (10)
7 trial.ti. (236085)	42 (peer* adj2 advisor*).tw. (46)
8 1 or 2 or 3 or 4 or 5 or 6 or 7 (1459955)	43 (peer* adj2 advocate*).tw. (74)
9 exp animals/not humans.sh. (4799281)	44 (peer* adj2 consultant*).tw. (39)
10 8 not 9 (1351468)	45 (peer* adj2 helper*).tw. (37)
11 *Peer Group/(9557)	46 (peer* adj2 implement*).tw. (329)
12 (Peer* adj support*).ab. (4633)	47 (peer* adj2 instructor*).tw. (94)
13 (Peer* adj3 support*).ti. (1421)	48 (peer* adj2 intervention*).tw. (1370)
14 (peer* adj1 led).tw. (1159)	49 (peer* adj2 listener*).tw. (6)
15 (peer* adj2 mentor*).tw. (924)	50 (peer* adj2 mediated).tw. (271)
16 (peer* adj2 program*).tw. (1638)	51 (peer* adj2 mentor*).tw. (924)
17 (peer* adj3 group*).ti. (650)	52 (peer* adj2 network*).tw. (914)
18 (peer* adj group*).ab. (2660)	53 (peer* adj2 provider*).tw. (233)
19 (peer* adj2 coach*).tw. (325)	54 (peer* adj specialist*).tw. (122)
20 (peer* adj2 counsel*).tw. (731)	55 (peer* adj2 trainer*).tw. (68)
21 (peer* adj2 deliver*).tw. (433)	56 (peer* adj2 trained).tw. (454)
22 (peer* adj2 educat*).tw. (2253)	57 (peer* adj2 tutor*).tw. (310)
23 (peer* adj2 expert*).ti. (30)	58 (peer* adj2 volunteer*).tw. (216)
24 (peer* adj1 expert*).ab. (122)	59 (peer* adj2 worker*).tw. (438)
25 (peer* adj2 facilitat*).tw. (547)	60 “peer based”.tw. (445)
26 (peer* adj2 leader*).tw. (487)	61 (lay adj2 leader*).tw. (131)
27 (peer* adj2 navigat*).tw. (133)	62 “mutual aid”.tw. (393)
28 (peer* adj2 provider*).tw. (233)	63 “mutual help”.ti, ab. (312)
29 (peer* adj specialist*).tw. (122)	64 “shared experience”.tw. (387)
30 (peer* adj2 trained).tw. (454)	65 (survivor* adj2 deliver*).tw. (129)
31 (peer* adj2 cofacilitat*).tw. (3)	66 (survivor* adj led).tw. (25)
32 (peer* adj2 co facilitat*).tw. (9)	67 (consumer* adj provide*).tw. (245)
33 “peer to peer”.ti. (293)	68 (consumer* adj deliver*).tw. (10)
34 (peer* adj1 led).tw. (1159)	69 “consumer case management”.tw. (8)
35 (peer* adj3 run*).ti. (26)	70 or/11‐69 (24942)
	71 10 and 70 (3190)

We also searched Google Scholar (via Publish or Perish; Harzing) to identify any additional relevant studies and the Cochrane Central Register of Controlled Trials (CENTRAL) and clinical trial registers (ClinicalTrials.gov and WHO ICTRP) for any ongoing RCTs in May 2021.

We did not restrict the Stage 2 searches using publication date or language database limits.

Manual checking of references and forward citation searching using Scopus (Elsevier), Web of Science (Clarivate Analytics) and Citation Chaser (https://estech.shinyapps.io/citationchaser) was conducted on SRs, RCTs and EEs that met our inclusion criteria. We did not contact individuals, organisations, or stakeholders to identify studies. All search strategies along with two search summary tables for Stage 1 and Stage 2 are reported in Supporting Infomation: Appendix 3.

Results from all searches were imported into EndNote X9.2 (Clarivate Analytics) and de‐duplication was conducted using a combination of EndNote duplicate detection options and manual checks. We removed RCTs and EEs included in SRs identified in Stage 1.

### 4.7 Analysis and presentation

#### 4.7.1 Description of intervention

As we were interested in any form of peer support, the included studies reported a diverse range of peer support interventions. We detail important characteristics of the interventions in the EGM, these are: description of peer support intervention; support structure for peers; structure of meetings; who facilitates the intervention; duration of intervention; location of intervention; and main focus of the research. Table 2 provides definitions of the subcategories within the description of peer support intervention category; further information on the subcategories within the additional characteristics is provided in the data extraction forms in Supporting Infomation: Appendix 4.

**Table 2:** Peer support intervention categories

Category	Definition/example
Case management, health service liaison	Helping participant to make contact with available health and social care support. This might include accompanying peers to appointments or identifying relevant services.
Education, coaching, mentoring	Provision of information, education, training, mentoring and/or coaching by the peer supporter. This could be through a manualised intervention covering specific topics or informal discussion.
Practical support for health behaviours	Practical help with health behaviours such as exercising, or learning to monitor blood glucose levels.
Psychological, emotional, wellbeing support	Mental health support, mindfulness, wellbeing, and quality of life interventions.
Self‐care, self‐management	A focus on self‐management, self‐care and goal setting.
Social, community	By definition all peer support includes a social element. This category related specifically to interventions which aimed to help peers build relationships, participate in the community, or support social interaction.
Not clearly defined	Interventions where information was not provided on the content of contact between peer and peer supporter.

#### 4.7.2 Description of outcomes

Any effectiveness outcome was included in the EGM; we grouped these into five broad categories: health‐related indicators; self‐regulation; supporting self‐regulation; wellbeing and social connectedness; cost‐effectiveness and service use; and experience of peer support. Table 3 lists subcategories within each category and gives examples of measures used for each in included studies.

**Table 3:** Examples of outcomes of interest in the EGM; with relevant definitions given in italics

Outcomes	Sub‐categories	Example
Health related	Physical health	Body Mass Index HIV viral load HCV relapse
	Mental health	Center for Epidemiologic Studies Depression Scale Warwick‐Edinburgh Mental Wellbeing Scale Brief Psychiatric Rating Scale
Self‐regulation *Skills for self‐management, and practising behaviours linked to health and social outcomes*	Self‐management *Learning and practicing skills to enable management of health and social needs on a day‐to‐day basis*	Patient Activation Scale Illness Management and Recovery Scale
	Health behaviour *Repeated behaviours that influence social and/or health outcomes*	Levels of physical activity Eating 5 portions of fruit/veg per day Housing stability
	Addiction recovery *Outcomes related to addictive behaviours; such as smoking cessation, or changes in substance abuse*	Smoking cessation Addiction Severity Index
Supporting self‐regulation *Skills, understanding and attitudes potentially linked to self‐regulation and management of health and social needs*.	Self‐efficacy *Measures that quantify mental state and attitudes that are likely to translate into health behaviours*	Patient Self‐Advocacy Scale Self‐esteem Hopefulness Personal empowerment
	Knowledge and understanding *Knowledge about own health condition and how to meet own needs*	Understanding of healthcare options Nutrition label reading confidence
Wellbeing and social connectedness	Wellbeing and quality of life	Lehman's Quality of Life Interview Health‐Related Quality of Life Scale Brief Symptom Inventory
	Social support and relationships	Martial interaction/satisfaction Community participation/integration Interpersonal Support Evaluation List
Cost‐effectiveness and service use	Cost‐effectiveness *Comparative analysis of two or more alternative interventions in terms of health, social and economic consequences*	Healthcare costs based on type and length of treatment
	Service use *Measure of use of any health, social or other services*	Number of emergency department admissions Readmission to acute services Appointment attendance
	Employment	Employment status
Experience of peer support	Experience of peer support	Service Engagement Scale Working Alliance Inventory
	Peer outcomes	Any outcome asked to peer supporters

Abbreviations: EGM, evidence and gap map; HIV, human immunodeficiency virus.

##### Filters for presentation

EGMs usually present two primary dimensions, different outcomes as columns and different interventions as rows (as discussed above). Additionally, we added the following filters to this EGM. These filter categories allow the user to change what subset of studies are shown in the map:

1. Type of study: SR of impact evaluations; RCT, or EE.
2. Population characteristics: these were broad categories indicating whether studies focused on populations with either acute or chronic mental or physical conditions, addiction difficulties, parents or carers of people with a health difficulty, and vulnerable people. More specific population/user characteristics were drawn from the text and included in study summaries in the EGM.
3. Age: young people (18–25); adults (26–64); older adults (65+); or not clearly defined.
4. Location of study: UK; Europe; USA or Canada; Australia or New Zealand; or Other.
5. Follow‐up time frame: at intervention end; up to 3 months; over 3, up to 6 months; over 6, up to 12 months; over 12, up to 24 months; over 24 months, up to 5 years; over 5 years, up to 10 years; lifetime; none specified.
6. Study quality: the EGM can be filtered by summary assessments of study quality, based on assessing the methodological quality of systematic reviews (AMSTAR 2) for SRs, Cochrane ROB for RCTs, or consensus health economic criteria list (CHEC) list for EEs.
7. Publication year

##### Dependency: Avoiding double counting of studies

SRs and impact evaluations (RCTs and EEs) are reported as separate items, with risk of bias represented independently as high or low for each. Where an impact evaluation is already included in a SR, it is not shown separately on the map. Whilst each item in the map details one study, we found some publications which used data from the same sample. In these cases, they were treated as a single study, with details of the additional publications provided at the end of the study abstract and a link to all relevant publications included. Although all SRs and impact evaluations have individual entries in the EGM, evidence does overlap as studies may be found in more than one cell if they report on multiple interventions or measure multiple outcomes.

### 4.8 Data collection and analysis

#### 4.8.1 Screening and study selection

Records from the search results were imported from bibliographic databases into EndNote libraries for screening. The screening was carried out based on predefined eligibility criteria [as specified in the EGM protocol (Shaw et al., 2021)] with pre‐specified mechanisms for dealing with situations such as where only a part of the population met eligibility criteria. It was undertaken in two stages: first for SRs and second for impact evaluations.

#### 4.8.2 Stage 1: Title and abstract screening

As an initial calibration exercise of inclusion judgements and the clarity of our inclusion criteria, all reviewers applied inclusion and exclusion criteria to the same sample of search results (*n* = 100 for SRs and *n* = 100 for impact evaluations). Decisions were discussed in a group meeting to ensure consistent application of criteria with some revisions made to enable more consistent reviewer interpretation of the criteria.

The revised inclusion and exclusion criteria (listed in Table 4; full details in Supporting Information: Appendix 2) were then applied to the title and abstract of each identified citation independently by two reviewers, with disagreements being resolved by discussion.

**Table 4:** Eligibility criteria for inclusion in the EGM

	Include	Exclude
Study design	SRs RCTs EEs	Any other study design
Population	Over 18 Defined health and social care need	Under 18 At risk population Engaging in high risk behaviours
Intervention	Any intervention where:	Any intervention where:
	peer supporters had a formalised role e.g. through training, and shared experience with peers e.g. same or similar health condition	peer supporters did not have a formal role e.g. mutual support groups peers did not have similar experience e.g. a difference health condition
Outcomes	Any outcome related to effectiveness	Outcomes related to the acceptability of the intervention, or which only focused on delivering peer support
Publication date	2015 onwards	

Abbreviations: EE, economic evaluation; EGM, evidence and gap map; RCT, randomised controlled trial; SR, systematic review.

#### 4.8.3 Stage 2: Full text

The full texts were obtained for papers that appeared to meet the inclusion criteria, and those for which a decision was not possible based on the information contained within the title and abstract alone. For SRs, the full text of each record was then assessed independently for inclusion by two reviewers. For impact evaluations, a second calibration exercise was undertaken by two reviewers for a sample of the full text articles (*n* = 50), before full texts were screened as for SRs. Disagreements were settled by discussion.

Ongoing SRs and RCTs were screened using the screening criteria listed in Table 4 and Supporting Information: Appendix 2. Where limited information at full text made it difficult to assess eligibility, this was discussed by the team, and a decision mechanism agreed and consistently applied. For example, for studies where it was implied, but not explicitly stated, that peers had the same health condition as participants (thus meeting our definition of peer support) these were included.

#### 4.8.4 Data extraction and management

We imported records from the EndNote libraries into our data management software, EPPI‐Reviewer 4 (Thomas et al., 2010). Standardised data extraction forms were developed for SRs, RCTs and EEs in EPPI‐Reviewer 4 (see Supporting Information: Appendix 4), to collect information on different aspects of peer support interventions, including the content, method, and duration, and outcomes. These were piloted by the review team on a selection of included studies.

To ensure an efficient use of limited resources, data for impact evaluations (RCTs and EEs) that were included in SRs was only extracted from information reported within the relevant SR. However, as the aims, methodology, and reporting of included SRs may have been slightly different to those of the EGM, extracted data on these impact evaluations is more limited than data extracted directly from RCTs and EEs. Data extraction was performed by one reviewer and checked by a second, with disagreements being settled through discussion.

Data were extracted from all included studies identified by the searches; these are listed under ‘Included studies’. An additional 4 SRs and 1 RCT were identified after the searches had been completed. Due to time constraints on the construction of the map, data was not extracted from these studies. They can be found in the list of ‘Studies awaiting classification’. Ongoing SRs and RCTs can be found in the list of ‘Ongoing studies’.

#### 4.8.5 Tools for assessing risk of bias/study quality of included reviews

Quality assessment of SRs, RCTs, and EEs was conducted to indicate confidence in study findings. All assessments were performed by one reviewer and checked by a second, with disagreements settled through discussion. We did not exclude any study based on study quality.

#### 4.8.6 AMSTAR 2

All SRs identified as eligible following full‐text screening were appraised using the AMSTAR2 quality appraisal tool (Shea et al., 2017) for SRs of primary studies of randomised and non‐randomised study designs. AMSTAR‐2 is a 16‐item checklist, covering all aspects of the conduct of a SR, from pre‐specifying a protocol to appropriately analysing and discussing risk of bias. Full details of the checklist can be found in Supporting Information: Appendix 5.

Items 2, 4, 7, 9, 11, 13 and 15 of the checklist are considered “critical” in assessing overall study quality, with studies rated from high to critically low depending on the number of weaknesses (Shea et al., 2017). A rating of “high” means the study has no more than one noncritical weakness, “moderate” that there is no critical weakness but more than one noncritical weakness, “low” that a study has one critical weakness, and “critically low” that a study has more than one critical weakness. Low and critically low studies may also have multiple noncritical weaknesses.

#### 4.8.7 ROB tool

We used the Cochrane ROB tool (Higgins et al., 2011; Higgins & Green, 2011) to assess risk of bias in included RCTs. As our EGM focuses on multiple outcomes, we were assessing all outcomes that met our inclusion criteria in the included studies for risk of bias. We therefore decided to use ROB rather than ROB2, as this tool is more suitable for considering multiple outcomes.

ROB considers trial design, conduct and reporting, focusing on seven domains related to the internal validity of the study: sequence generation, allocation concealment, blinding of participants and personnel, blinding of outcome assessment, incomplete outcome data, selective outcome reporting and other biases. Review authors discussed the application of the domains to the studies throughout the process of critical appraisal. For the selective reporting domain, as we were not performing a meta‐analysis, we considered risk of bias to be low if all measured outcomes were reported in the results; these could be in any format. ‘Other biases’ are normally prespecified: we did not consider there to be any specific issues relating to peer support so did not use this domain.

As well as considering risk of bias due to each domain/study feature, review authors designate key domains in relation to the review topic which inform a summary of overall risk of bias for a study (Higgins & Green, 2011). Due to the difficulties of blinding in studies of peer support, the study team decided that using the sequence generation, incomplete outcome data, and selective reporting domains would be most indicative of overall risk of bias.

#### 4.8.8 CHEC list

We used the CHEC list (Evers et al., 2005) for assessing the quality of EEs. The CHEC‐list was developed using expert consensus; it is a series of yes/no questions focusing on the methodological quality of EEs, and includes items on generalisability and the distribution of impacts across different groups or users (i.e., equity considerations). It can also be used to assess the quality of comparative costing studies, although in these cases the items relating to outcome identification, measurement and valuation are not applicable. To derive an overall assessment of quality so that EEs could be grouped with RCTs on the map, we calculated the percentage of applicable questions which were answered ‘yes’ (Wijnen et al., 2017). Studies scoring over 75% were classified as high quality, studies between 50% and 74% as medium quality, and studies below 50% as low quality (Ahumada‐Canale et al., 2019).

#### 4.8.9 Methods for mapping

EPPI‐Mapper was used to visualise the data entered in EPPI‐Reviewer 4 for each study as an interactive map (Digital Solution Foundary and EPPI‐Centre, 2020). We produced a brief narrative synthesis to accompany the map, this describes the spread and concentration of studies found across the different intervention and outcome categories as well as the filters for the map (White et al., 2020). Tables and figures were used to visualise the analysis (Saran & White, 2018).

## 5 RESULTS

### 5.1 Description of studies

In this section, we describe the 91 studies (reported in 100 publications) included in this EGM. In figures and tables, the number of studies shown is the total number of studies in the category. Individual studies may be included in more than one category, as some studies measure multiple outcomes or represent a number of different intervention types. This means the sum of studies for a figure, table, or in a descriptive summary, may be greater than the number of unique studies included within it.

#### 5.1.1 Results of the search

**Figure 1:**
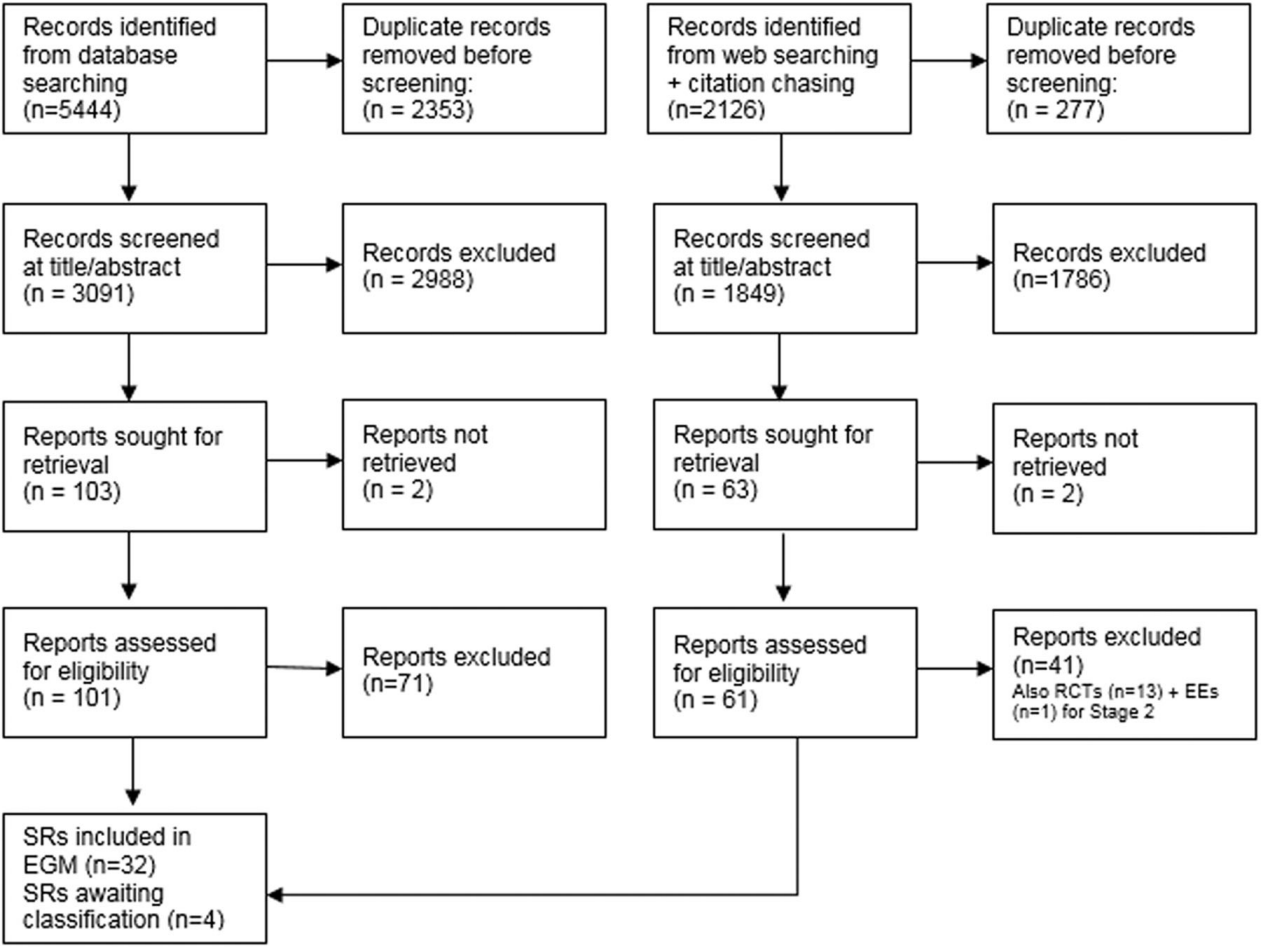
PRISMA flow diagram for SRs. PRISMA, preferred reporting items for systematic reviews and meta‐analyses; SR, systematic review.

**Figure 2:**
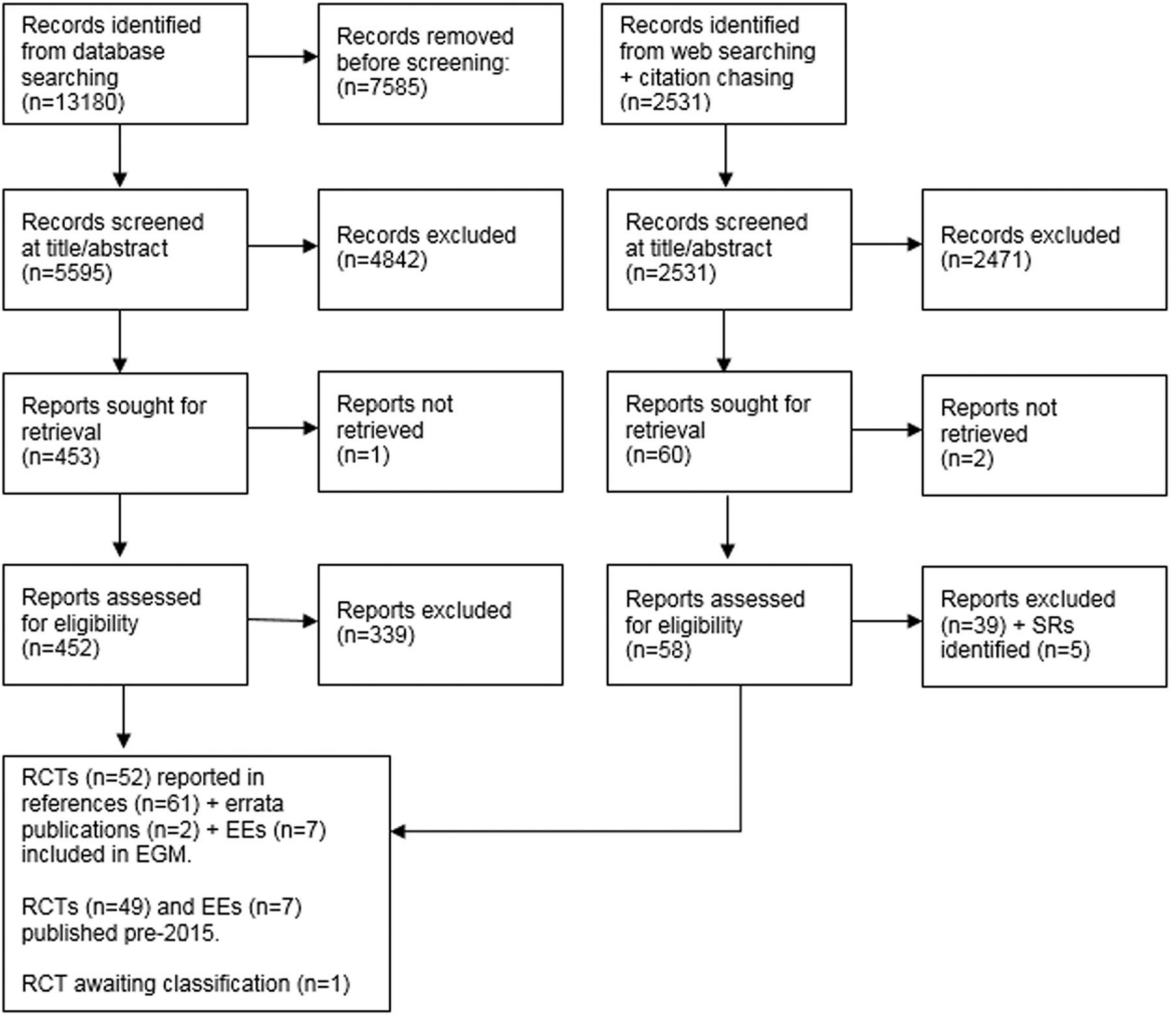
PRISMA flow diagram for impact evaluations. PRISMA, preferred reporting items for systematic reviews and meta‐analyses.

Figure 1 provides an overview of the search and screening process for SRs, Figure 2 shows this information for impact evaluations (RCTs and EEs). Bibliographic database searches retrieved 5444 records for Stage 1 and 13,180 records for Stage 2, with 2531 additional records identified through Google Scholar searches and citation chasing. After deduplication, 3091 records for Stage I, 5595 records for Stage 2, and 2531 records from other sources were double screened at title and abstract. Following this, full texts of 103 reports for Stage 1, 452 reports for Stage 2, and 58 reports (from other sources) were assessed for eligibility.

We included 91 studies in the EGM: 32 SRs (that included syntheses of evidence from 242 impact evaluations); and 59 impact evaluations (not included in a SR), made up of 52 RCTs (reported in 61 publications) and 7 EEs. Please note, that the results reported below reflect data either captured from the 32 SRs, or from the 59 impact evaluations. Findings from the 242 impact evaluations synthesised in included SRs are not reported individually.

#### 5.1.2 Excluded studies

Studies excluded after screening at full text, along with reasons for exclusion, can be found in the list of ‘Excluded studies’. Primary reasons for exclusion were that the peer support intervention or study design did not meet our inclusion criteria.

#### 5.1.3 Studies awaiting classification

One SR and four RCTs are awaiting classification in the EGM, all were published after the last searches had been completed. They can be found in the list of ‘Studies awaiting classification’.

A number of ongoing studies were identified for inclusion: 31 SRs from PROSPERO and 55 RCTs from trial registeries. Additonally, as a result of searching trial registries, we found 27 completed RCTs for which there are currently no published results and 5 RCTs with unknown completion status which met our inclusion criteria. These studies can be found in the list of ‘Ongoing studies’.

### 5.2 Synthesis of included studies

This EGM includes recent SRs and impact evaluations (RCTs and EEs), with recent defined as those published after 2015. SRs published per year have remained fairly constant since 2015, varying from 3 to 8 between 2015 and 2020. Our initial search for impact evaluations was not limited by date but as recent SRs will include studies before their publication date, we only included impact evaluations published after 2015 and that were not in an included SR in the EGM. We found 59 impact evaluations (52 RCTs and 7 EEs) meeting our inclusion criteria published before 2015 that were not in any included SR (a list of these studies can be found in Supporting Information: Appendix 6) and 242 impact evaluations that were already in an included SR (a list of these studies can be found in Supporting Information: Appendix 7). Of the 242 impact evaluations already in any SR, this EGM only includes data captured from the relevant SR, as data extraction was not carried out on each of the 242 impact evaluations individually. For impact studies not included in any SR (59), this EGM includes data extracted from the original study. As more recently published RCTs are less likely to be in SRs, there were increasing numbers of included RCTs over time, from 2 in 2015 to 12 in 2020. EEs varied between 0 and 2 published per year, as can be seen in Table 5.

**Table 5:** Number of included studies published since 2015

Year	SR (*n* = 32)	RCT (*n* = 52)
2015	4	2
2016	7	3
2017	3	9
2018	3	12
2019	6	7
2020	8	12
2021	1	7

Abbreviations: RCT, randomised controlled trial; SR, systematic review.

**Figure 3:**
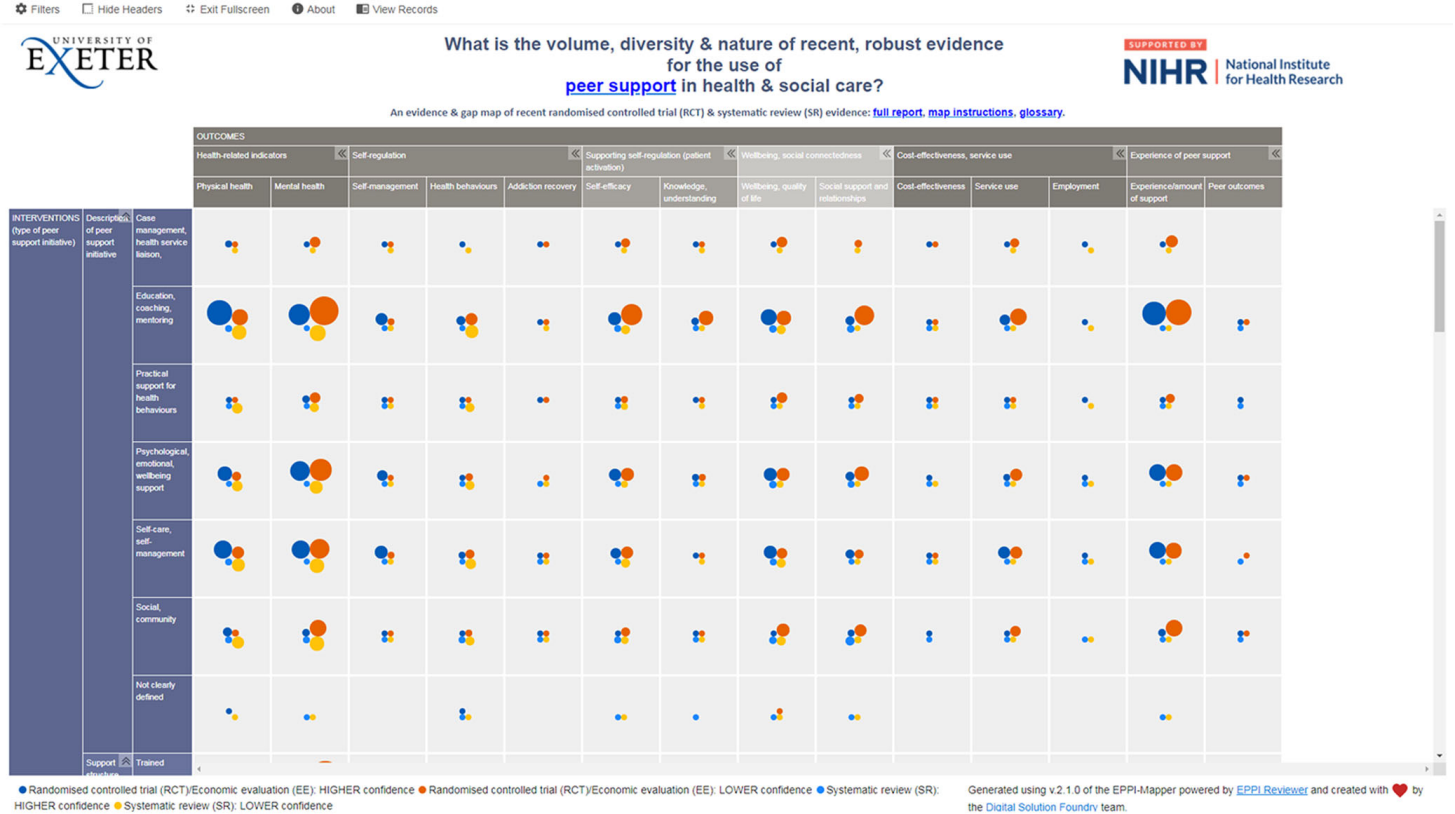
EGM of included studies, showing intervention and outcome categories (sub‐categories can be accessed in the interactive map) and study quality/risk of bias (blue indicates higher quality and orange lower quality). EGM, evidence and gap map.

Included studies can be viewed by visiting the online EGM in Supporting Information: Appendix 8. The map is accompanied by detailed instructions for use and a structured abstract with a summary of key charateristics is available for each study. Figure 3 presents an example of the interactive EGM, with the intervention and outcome categories displayed at the sides of the map; circle size indicates the number of studies found in the cell and colour represents study type and quality.

### 5.3 Distribution by intervention

Included studies described a wide range of peer support interventions. All SRs and the majority of impact evaluations (30 RCTs and 4 EEs) included research in which peer support was the main focus (i.e. component) of the intervention, although some studies did report the inclusion of peer support as part of a more complex intervention (5 SRs, 22 RCTs, and 3 EEs).

Below, we detail where we found evidence regarding the content of peer support interventions, the delivery of these interventions, and logistics, in terms of who facilitated the intervention and their support structures.

#### 5.3.1 Description of peer support intervention

**Figure 4:**
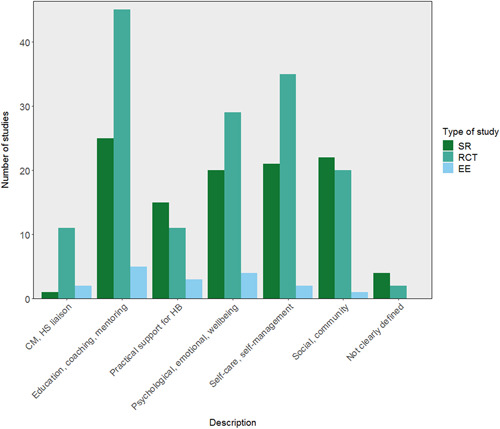
Number of included studies reporting each description of peer support intervention category (SRs including 242 impact evaluations). CM, case management; EE, economic evaluation; HB, health behaviours; HS, health service; RCT, randomised controlled trial; SR, systematic review.

There were several areas of evidence concentration, including both SRs and impact evaluations, regarding the description of peer support interventions. Most studies included in the EGM described peer support interventions including education, coaching, and mentoring (25 SRs, 45 RCTs, and 5 EEs), with over half of studies also reporting psychological and emotional peer support, or peer support including self‐care and self‐management (Figure 4). There was a lack of SRs including case management and health service liaison but 11 RCTs focused on this type of peer support. A greater proportion of SRs (22 studies) than RCTs included social and community peer support.

Table 7 shows an aggregate map of outcome categories and described peer support interventions for SRs. Health‐related indicators are measured in the majority of described peer support interventions, there is also a concentration of evidence among social and community interventions measuring wellbeing and social connectedness. Table 8 indicates similar concentrations of evidence among RCTs regarding health‐related indicators. Other populated areas in RCTs include education, coaching and mentoring interventions measuring outcomes relating to supporting self‐regulation and wellbeing, and both psychological, emotional, and wellbeing support, and self‐care and self‐management interventions measuring wellbeing‐related outcomes.

**Table 6:** Risk of bias in included studies for SRs (AMSTAR 2, *n* = 32), RCTs (ROB, *n *= 52), and EEs (CHEC, *n* = 7)

	Risk of bias	*N*
AMSTAR 2	High overall confidence in results	5
	Moderate confidence in results	6
	Low confidence in results	13
	Critically low confidence in results	8
ROB	Low risk of bias	24
	Unclear risk of bias	16
	High risk of bias	12
CHEC	Low risk of bias	1
	Medium risk of bias	4
	High risk of bias	2

Abbreviations: AMSTAR 2, assessing the methodological quality of systematic reviews; CHEC, consensus health economic criteria list; EE, economic evaluation; RCT, randomised controlled trial; ROB, Cochrane risk of bias tool; SR, systematic review.

**Table 7:** Aggregate map showing the number of SRs (*n* = 32) by description of peer support intervention category and outcome category, with colours indicating greater (purple) and lesser (blue) concentrations of evidence

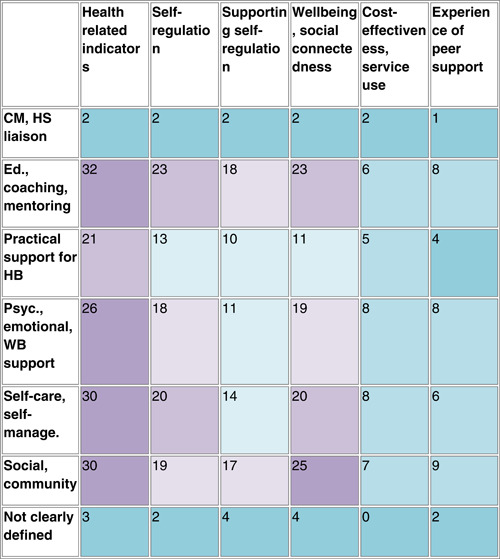

Abbreviations: CM, case management; HB, health behaviour; HS, health service; RCT, randomised controlled trial.

**Table 8:** Aggregate map showing the number of RCTs by description of peer support intervention category and outcome category, with colours indicating greater (purple) and lesser (blue) concentrations of evidence

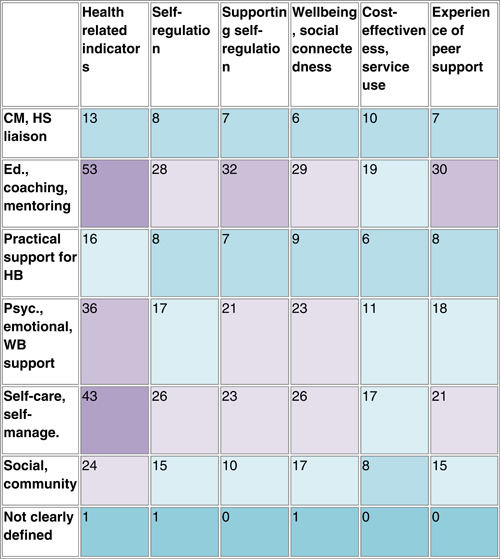

Abbreviations: CM, case management; HB, health behaviour; HS, health service; RCT, randomised controlled trial.

#### 5.3.2 Delivery of peer support intervention

**Figure 5:**
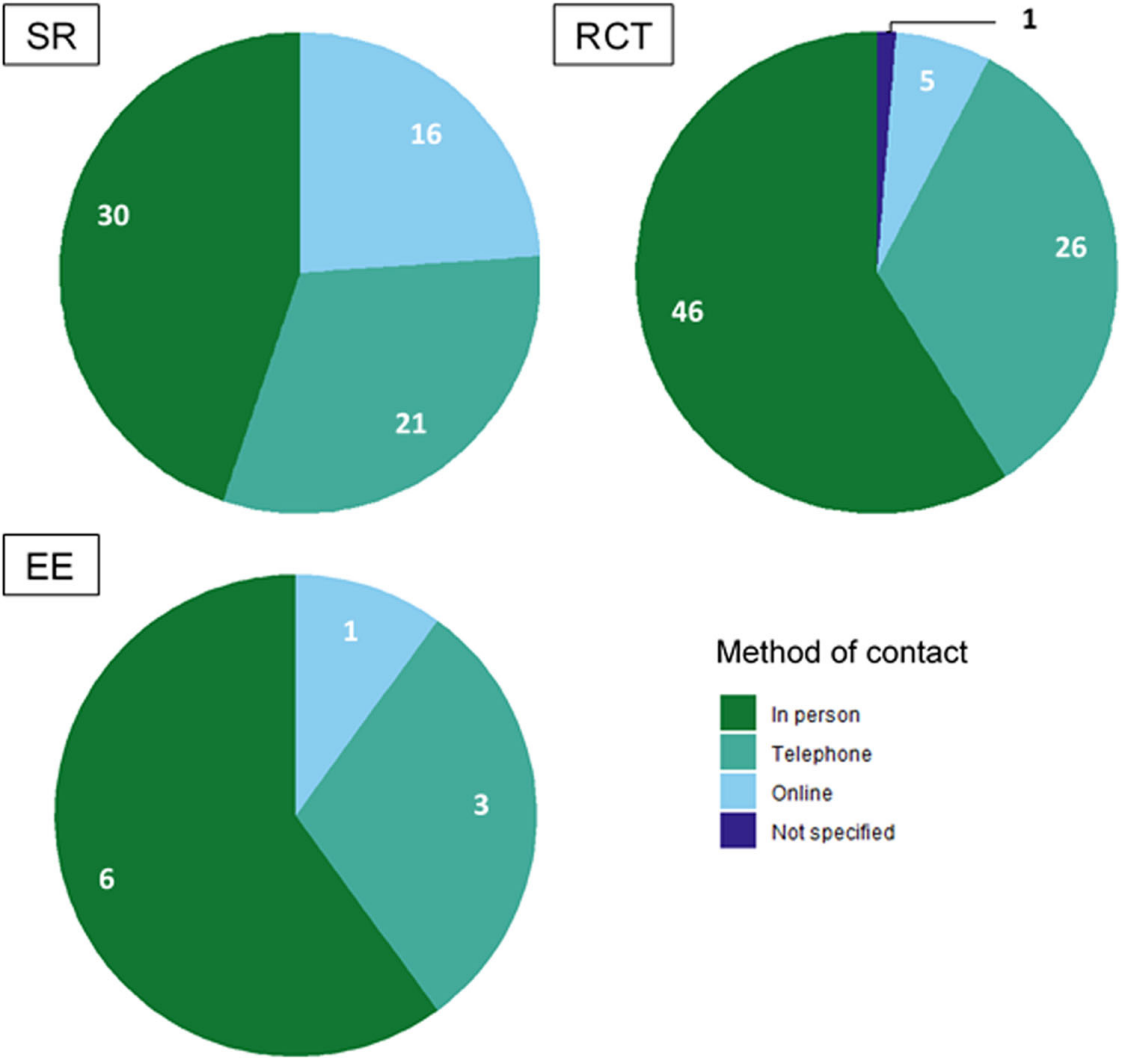
Number of included studies reporting each method of contact (SRs including 242 impact evaluations). EE, economic evaluation; RCT, randomised controlled trial; SR, systematic review.

**Figure 6:**
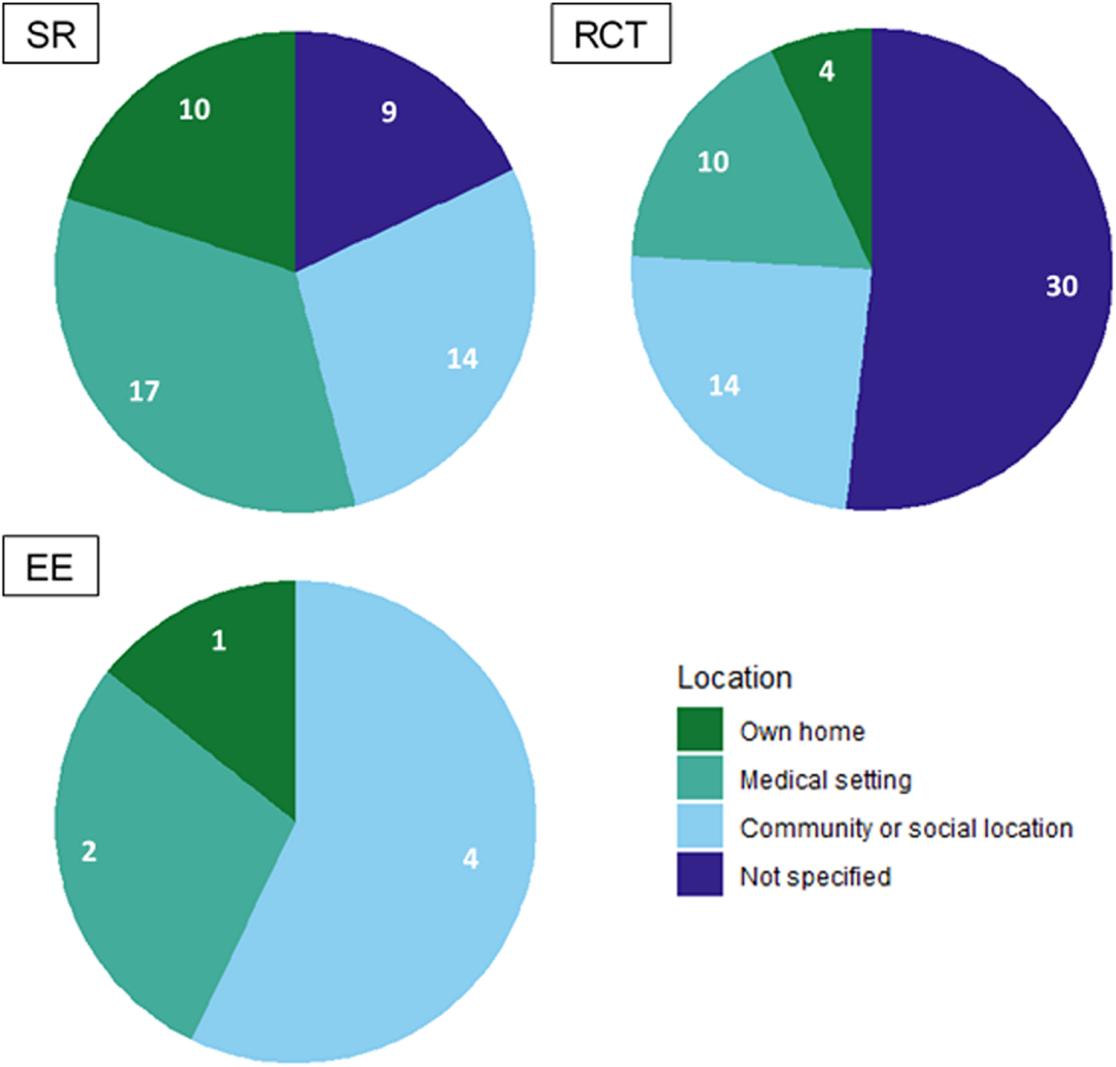
Number of studies reporting each location category (SRs including 242 impact evaluations). EE, economic evaluation; RCT, randomised controlled trial; SR, systematic review.

**Figure 7:**
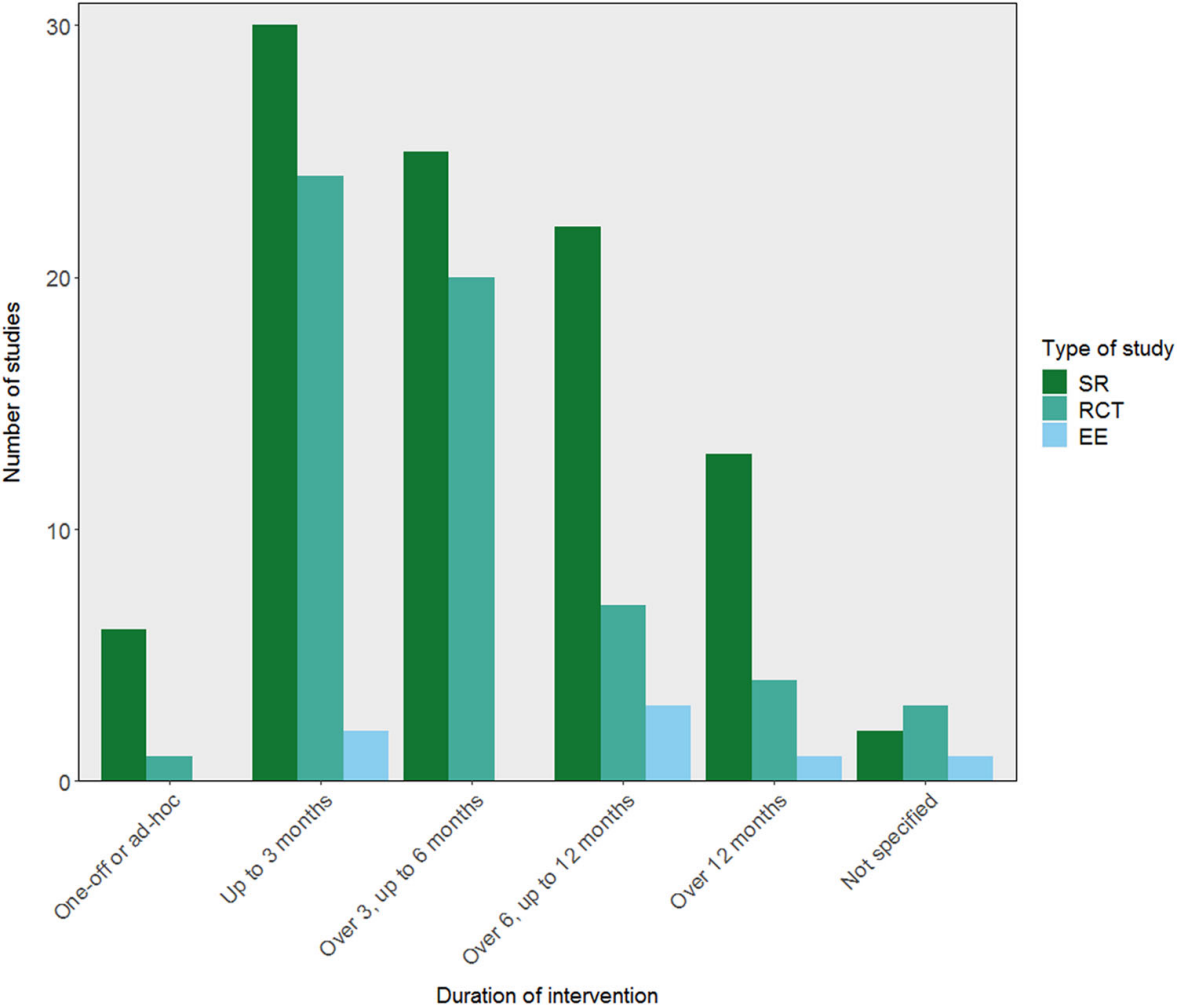
Duration of interventions reported by included studies (SRs including 242 impact evaluations not already in the map). EE, economic evaluation; RCT, randomised controlled trial; SR, systematic review.

Whilst almost equal numbers of SRs included in the map reported on peer support delivered one‐to‐one (26 studies) and in groups (27 studies), peer support delivered one‐to‐one was the most populated section of the map for impact evaluations (40 RCTs and 7 EEs). There was a concentration of studies on peer support delivered in person (30 SRs, 46 RCTs and 6 EEs; Figure 5). Half of SRs reported on online peer support but few RCTs (5 studies). Whilst there was a concentration of evidence on peer support delivered in medical or community settings in SRs, the location of the intervention was a gap in RCTs, with this being unspecified in 30 studies (Figure 6). Over half of EEs took place in a community or social location. Both SRs and RCTs tended to report peer support interventions of shorter duration, with categories of ‘up to 3 months’ and ‘up to 6 months’ containing the most evidence (Figure 7), although interventions of up to 12 months were most commonly reported in EEs.

#### 5.3.3 Logistics of peer support intervention

**Figure 8:**
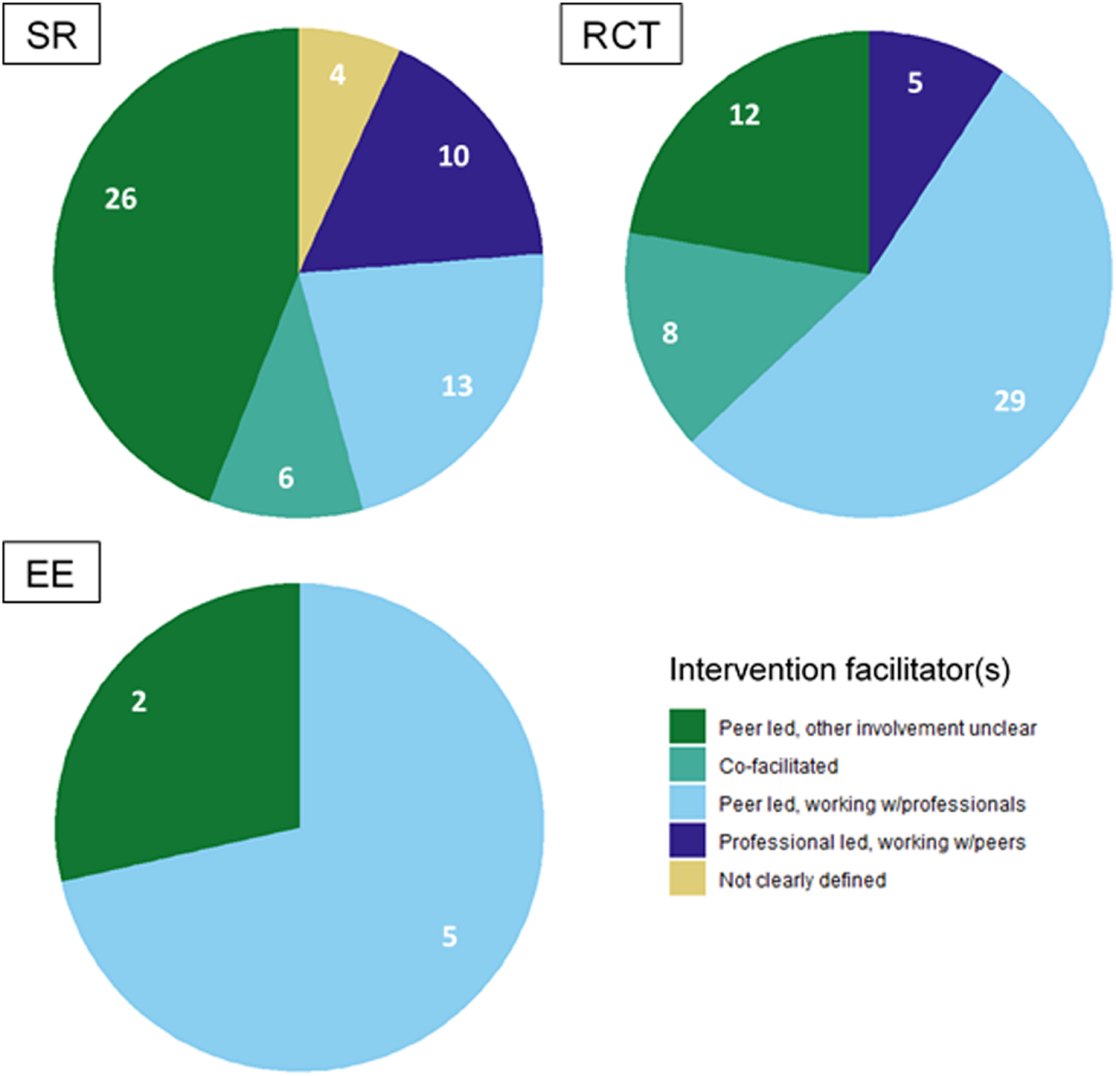
Intervention facilitators reported in the included studies (SRs including 242 impact evaluations not already in the map). EE, economic evaluation; RCT, randomised controlled trial; SR, systematic review.

There were some concentrations of evidence regarding who facilitated peer support interventions. Twenty‐six SRs reported on studies in which peers facilitated but it was not clear how professionals were involved although this was not the case among RCTs (12 studies). The next most populated category was interventions led by peers working with professionals (13 SRs, 29 RCTs, and 5 EEs). As can be seen in Figure 8, few studies reported on interventions co‐facilitated by peers and professionals, with only 6 SRs, 8 RCTs, and no EEs including evidence on this type of facilitation.

Our inclusion criteria set out three types of support, of which peers needed to have at least one to be included. Of these, training was the most populated category of support (25 SRs, 49 RCTs, and 7 EEs). Fewer studies included peers who were paid or had a contract (9 SRs, 18 RCTs, and 2 EEs).

#### 5.3.4 Methods used in included EEs

**Figure 9:**
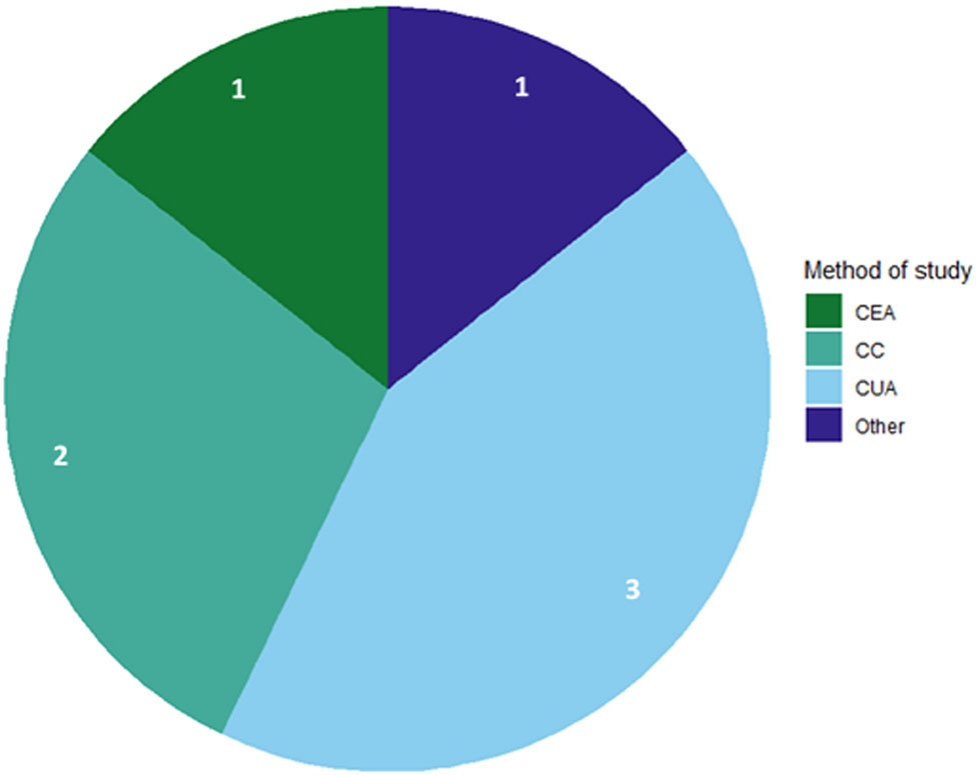
Methods used by included EEs (*n* = 7). CC, comparative costing; CEA, cost‐effectiveness analysis; CUA, cost‐utility analysis; EE, economic evaluation; RCT, randomised controlled trial; SR, systematic review.

Only 2 of the included EEs were model‐based, 5 used data from existing sources such as RCTs. Three of the EEs were linked to RCTs also included in the map. Cost‐utility analyses were the most commonly used method for assessing the cost‐effectiveness of peer support interventions (3 studies); two EEs were comparative costing studies, with the methods used by the remaining EEs being cost‐effectiveness analysis and other (cost‐offset) (Figure 9).

#### 5.3.5 Time period for follow‐up

As with the duration of the intervention, there was a concentration of evidence regarding follow‐up of interventions in the ‘up to 6 months’ category (24 SRs and 42 RCTs), with a large number of studies also reporting follow‐up at ‘up to 12 months’ (26 SRs and 23 RCTs). Longer follow‐up periods were less common except in EEs.

### 5.4 Study populations

**Figure 10:**
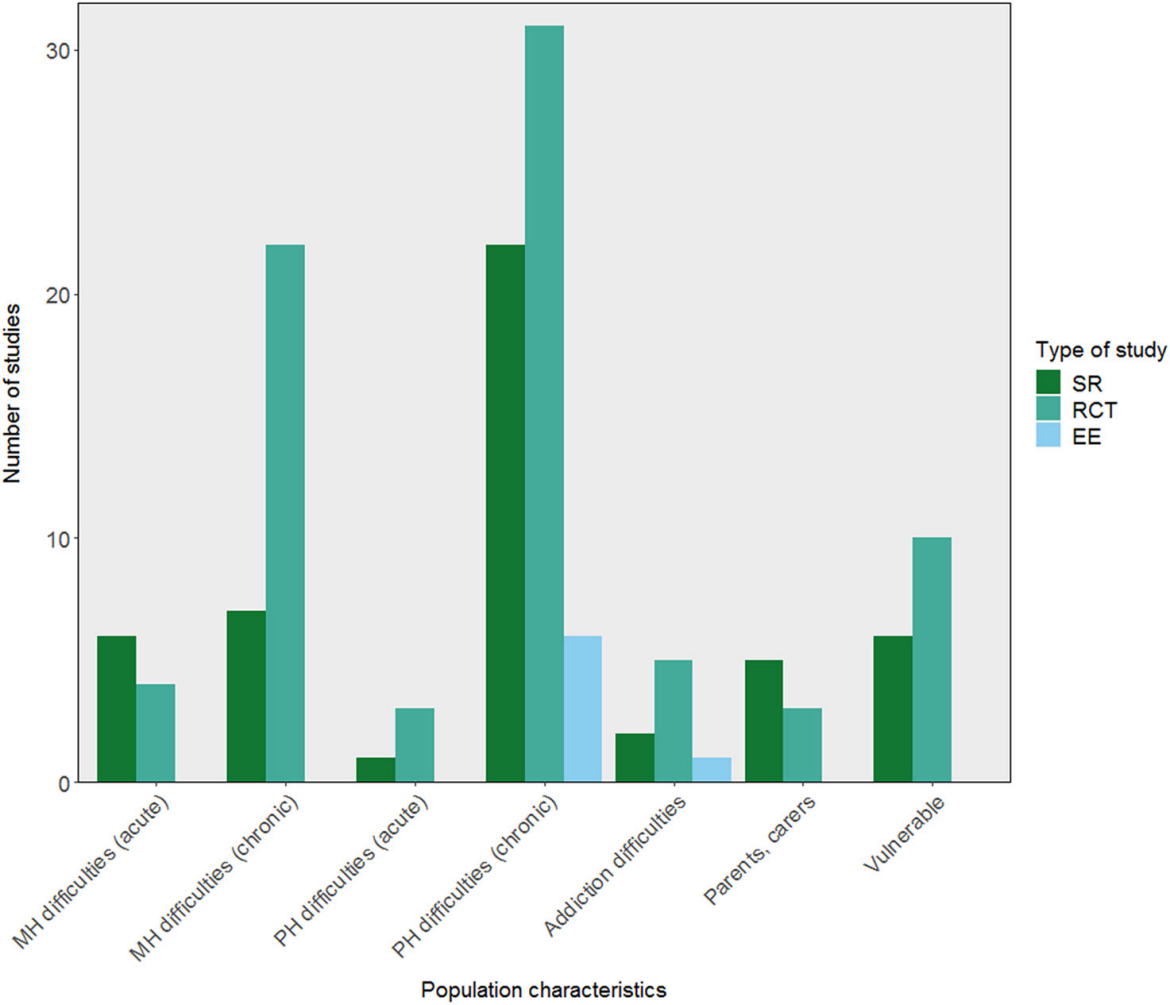
Number of included studies reporting on each population category (SRs including 242 impact evaluations). EE, economic evaluation; MH, mental health; PH, physical health; RCT, randomised controlled trial; SR, systematic review.

Included studies tended to focus on populations with chronic health difficulties, as shown in Figure 10. There was a concentration of evidence for chronic physical health difficulties for SRs (22 studies) and impact evaluations (31 RCTs and 6 EEs). Chronic mental health difficulties was the next most populated category for SRs and RCTs although a greater proportion of RCTs focused on chronic mental health difficulties than SRs (Figure 10). Evidence on acute health difficulties was limited, particularly among SRs for physical health, with only 1 study containing evidence for this category, and for both acute physical and mental health for RCTs. There were also few studies on those with addiction difficulties and parents and carers.

In terms of age, included studies mainly reported evidence on the use of peer support in adults aged between 26 and 64 (24 SRs, 50 RCTs and 6 EEs) or older adults. A greater proportion of SRs (19 studies) than RCTs (18 studies) reported evidence from older adults. Evidence on young people aged 18–25 was reported in only 10 RCTs.

### 5.5 Distribution by outcome category

**Figure 11:**
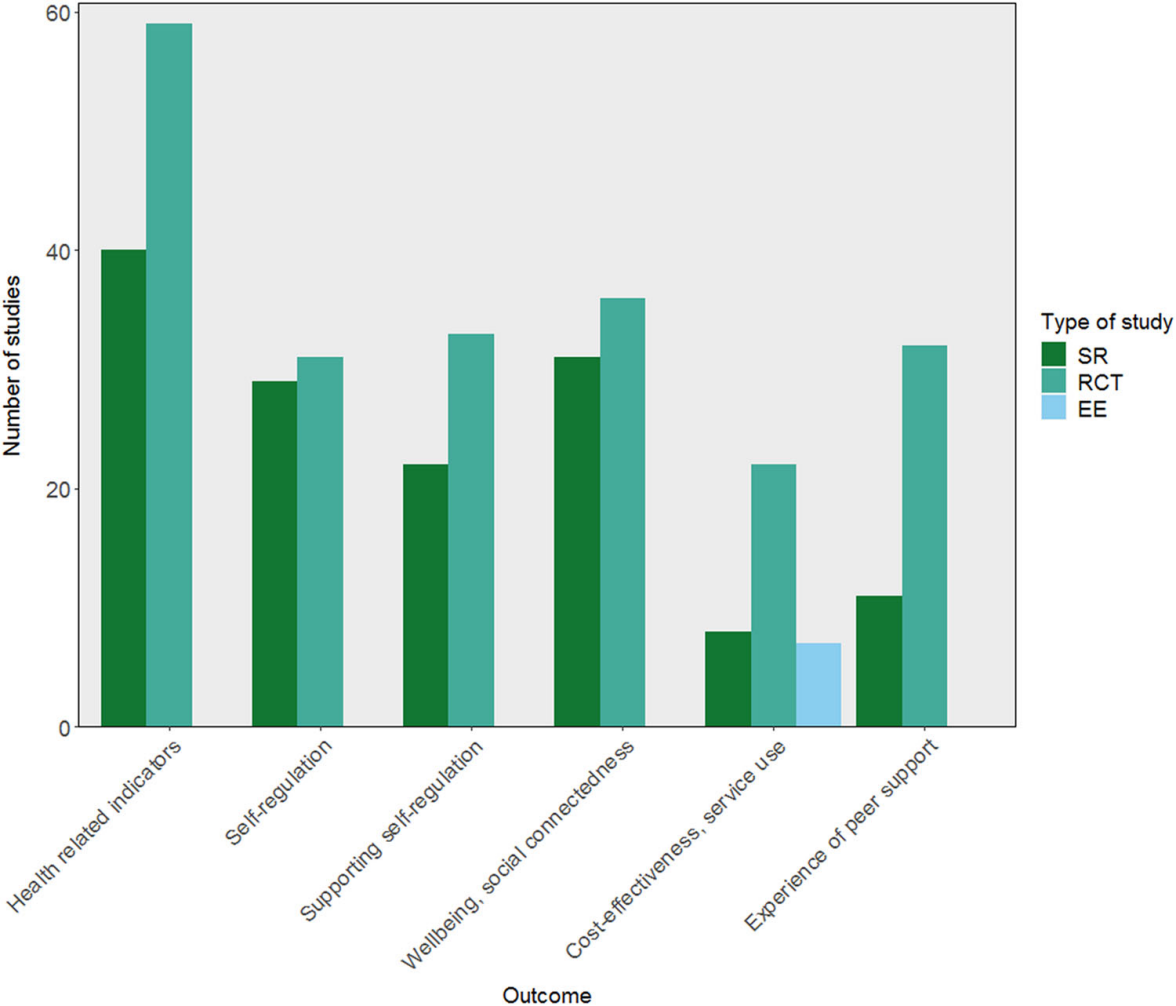
Number of included studies reporting each outcome category (SRs including 242 impact evaluations). EE, economic evaluation; RCT, randomised controlled trial; SR, systematic review.

Any outcome relating to effectiveness of peer support was included in the map. Figure 11 shows that distributions of SRs and RCTs were similar within our five broad outcome categories (Table 3). Of these, health‐related indicators, which include measures of physical and mental health, were the most populated section of the map for both SRs (40 studies) and RCTs (52 studies). A large number of studies measured self‐regulation (29 SRs and 31 RCTs) and wellbeing and social connectedness (31 SRs and 36 RCTs); there was also a concentration of RCTs (33 studies) with outcomes focused on supporting self‐regulation. Excluding EEs, cost‐effectiveness and service use was the least populated outcome category in the EGM (Figure 11).

There were some differences between subcategories in the concentrations of evidence from SRs and RCTs. Of studies measuring self‐regulation, a greater proportion of SRs measured health behaviours than RCTs. Within the cost‐effectiveness and service use category, whilst all 7 EEs measured cost‐effectiveness, this was an outcome in only 3 SRs and 1 RCT. However, there was a concentration of evidence regarding service use in RCTs (20 studies). A greater proportion of RCTs (30 studies) than SRs (9 studies) measured experience or amount of peer support, due to the reporting of the number of sessions or time participants spent with peer supporters.

### 5.6 Distribution by location

**Figure 12:**
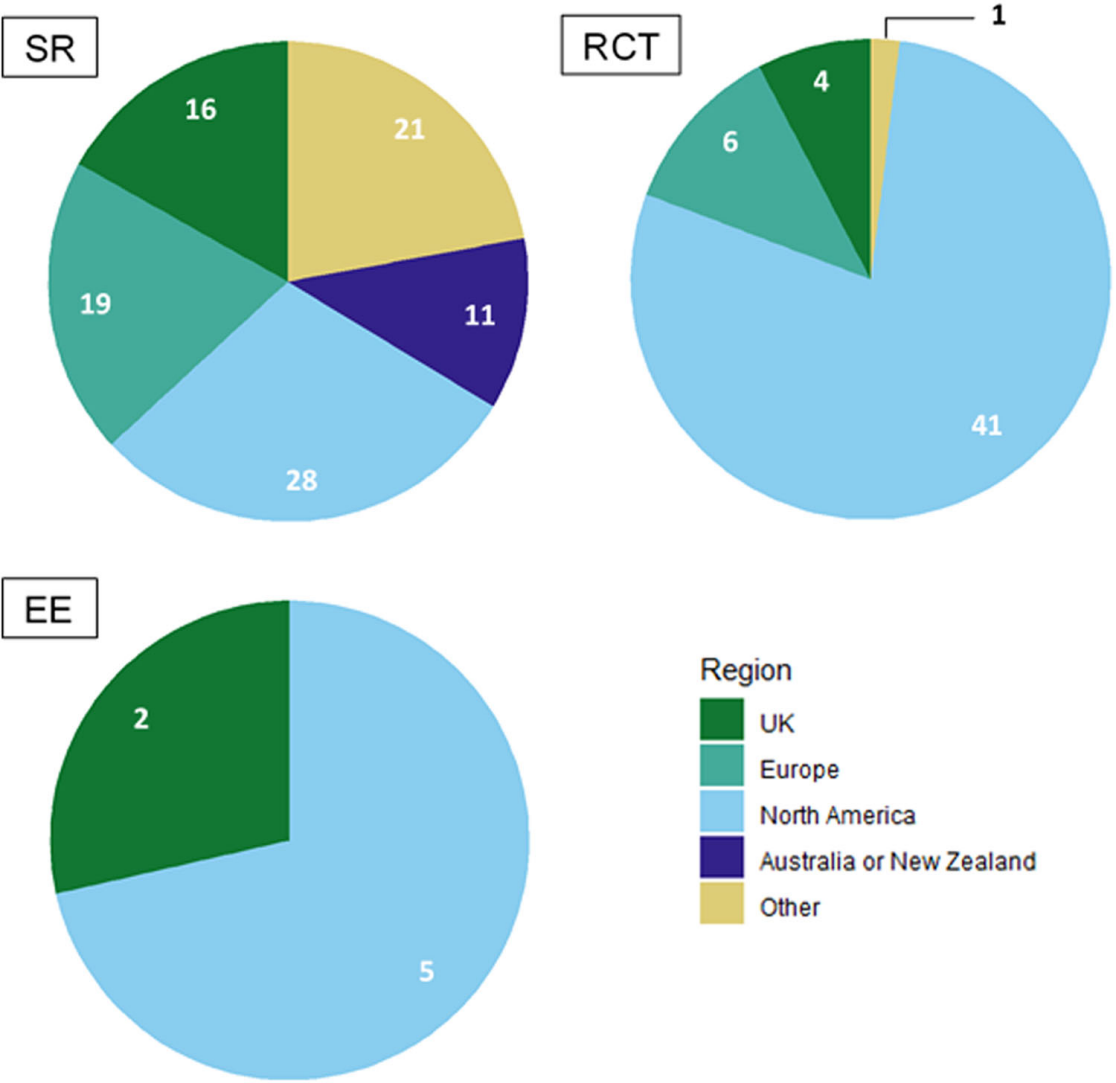
Geographic distribution of included studies (SRs including 242 impact evaluations). EE, economic evaluation; RCT, randomised controlled trial; SR, systematic review.

Included studies were concentrated in North America, with the majority of SRs (28 studies), RCTs (41 studies), and EEs (5 studies) reporting evidence from the USA or Canada. Whilst 16 SRs reported on studies from the UK, this was an evidence gap for impact evaluations, with only 4 RCTs and 2 EEs being based in the UK. After North America, the most common location for RCTs to be conducted was Europe (6 studies) (Figure 12).

### 5.7 Risk of bias in included studies

Table 6 show the results of quality assessment of all studies included in the EGM; risk of bias ratings for individual studies can be found in Supporting information: Appendix 9. Study quality did not vary significantly between map dimensions for SRs and RCTs, with similar proportions of low and high quality studies found in different intervention and outcome categories.

Included SRs (32 studies) were assessed for risk of bias using AMSTAR 2 (Shea et al., 2017), which indicated that overall confidence in the results of most SRs was critically low (8 studies) or low (13 studies). Only five SRs were considered to have a high overall level of confidence. Half of SRs were considered to have high risk of bias due to the lack of a protocol, whilst there was an unclear risk of bias regarding the search strategy in 78% (25 studies) and the details of excluded studies in 72% (23 studies). Most (72%, 23 studies) used satisfactory techniques to assess risk of bias in included RCTs and discussed these adequately in the results (84%, 27 studies).

**Figure 13:**
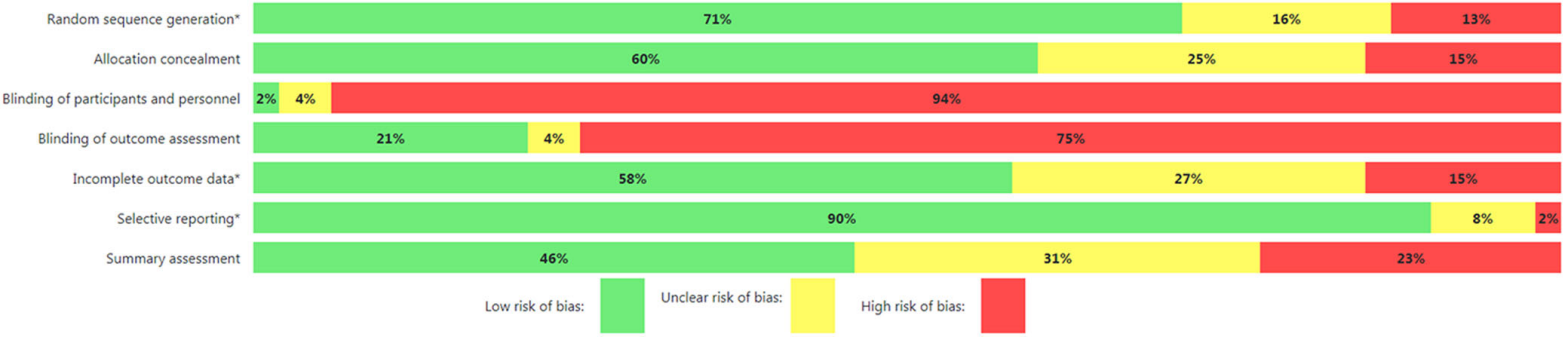
Risk of bias diagram for RCTs. *Key domains used to calculate summary assessment. RCT, randomised controlled trial.

ROB (Higgins et al., 2011; Higgins & Green, 2011) was used to assess RCTs (52 studies); key domains used to calculate overall risk of bias for RCTs can be seen in Figure 13. Overall, the majority of RCTs had a low risk of bias (24 studies). Certain domains, relating to blinding, were consistently at high risk of bias in the included RCTs. Only one study adequately blinded participants and personnel, 94% (49 studies) had a high risk of bias for this domain. Similarly, for blinding of outcome assessment, 75% (39 studies) had a high risk of bias. Almost all studies had a low risk of bias in terms of selective reporting (90%, 47 studies) and random sequence generation (71%, 37 studies). Most studies also had a low risk of bias for allocation concealment (60%, 31 studies) and incomplete outcome data (58%, 30 studies), although for both of these domains a significant proportion of studies had an unclear risk of bias (25%/13 studies for allocation concealment and 27%/14 studies for incomplete outcome data) (Figure 13).

For EEs, all except one were judged to be of low or medium quality using CHEC (Evers et al., 2005). No studies satisfied the criteria for whether the generalisability of the results was adequately discussed (CHEC item 17) or whether the article notes ethical aspects, characteristics of the population experiencing the disease/intervention and potential distributional implications (CHEC item 19). Only two studies were judged to have comprehensively explored uncertainty through sensitivity analysis, which is usually seen as an essential feature of a high quality economic evaluation (where, in general, few variables tend to be known with absolute certainty). Of the four economic evaluations, three tended not to clearly report or adequately justify: the time horizon of the analysis, the perspective of the analysis, and the separate presentation and justification of which resources were included, that they were counted in relevant physical units, and that they were valued appropriately. Two of the three costing analyses met these criteria more convincingly, and both were judged to be of ‘medium’ study quality. Looking at the overall pattern of strengths and weaknesses, Campbell (2014), Ye et al. (2021) and Patel et al. (2017) provide the most valid and reliable evidence about the cost‐effectiveness of those peer support interventions [respectively: for people with Type 2 diabetes in the USA (Campbell, 2014; Ye et al., 2021) and for people with latent TB in Canada (Patel et al., 2017)].

## 6 DISCUSSION

### 6.1 Summary of main results

This EGM focuses on the effectiveness of peer support interventions in health and social care. Overall, we found a considerable volume of studies, distributed across many categories in the map. There were some areas where there was a lack of SRs but a number of RCTs, or vice versa. However, there were some areas of clear evidence concentration or absence for all types of included studies, these are discussed below.

### 6.2 Overall completeness and applicability of evidence

Assessment with ROB indicated a concentration of high quality RCTs in the map, with substantial evidence from SRs and RCTs found for several types of peer support:

- education, coaching, and mentoring;
- psychological, emotional and wellbeing support;
- self‐care and self‐management;
- social and community.

Education, coaching and mentoring contained the most studies of any category describing the type of peer support intervention, as a result they also had the most evidence relating to all types of outcome.

Studies tended to focus on peer support for populations with chronic health difficulties, as do the majority of studies awaiting classification (Berg et al., 2021; Ghahramani et al., 2021; Lyons et al., 2021; Wan et al., 2021). As might be expected, we found a concentration of evidence focusing on health‐related indicators, both physical and mental, of the effectiveness of peer support. Outcomes relating to wellbeing and social connectedness, and among RCTs self‐regulation, were also well represented.

There was a significant concentration of studies conducted in the USA. Regarding the delivery of peer support, there was a concentration of evidence on support delivered in person. Both SRs and RCTs tended to focus on shorter interventions, of up to 3 or up to 6 months.

### 6.3 Areas of major gaps in the evidence

There was a lack of studies reporting on case management and health service liaison as a type of peer support. Significant gaps were also seen for some population groups, particularly those with acute physical health difficulties. Despite some EEs meeting our inclusion criteria, there was a clear evidence gap relating to the cost‐effectiveness of peer support, with CHEC also indicating that the majority of these studies were of low quality. There was an evidence gap in relation to the quality of SRs, with AMSTAR 2 indicating a low overall confidence in results for the majority of SRs, though no patterns were seen in the outcomes, type, or delivery of peer support reported by these SRs.

The geographic distribution of studies had gaps, with limited evidence available for the UK and Europe. In terms of the delivery of peer support, there were a lack of RCTs reporting on online peer support or specifying the location of the intervention. There were also gaps concerning peer support co‐facilitated by peers and professionals, and the duration of the intervention, particularly for interventions delivered over a longer time frame.

### 6.4 Limitations of the EGM

The volume of literature on the use of peer support in health and social care meant that we had to use strict inclusion and exclusion criteria. Whilst these criteria were based on consultation with stakeholders, they do introduce limitations to the evidence shown in the map. Our criteria regarding training and support for peer supporters may have meant the inclusion of more formal interventions was more likely whilst some community‐organised initiatives may have been excluded. As we excluded at‐risk populations and those engaging in high risk behaviours, such as heavy drinking or drug use, this meant that peer support interventions intended to increase preventative health measures such as health screening or vaccinations were largely excluded from the map. Similarly, our decisions around multi‐component interventions (those including peer support as one component) mean that some relevant evidence may have been excluded (Lobban et al., 2020).

Reporting of certain details in the included studies was sometimes unclear. This makes it difficult to determine what evidence is available, particularly regarding the facilitation of peer support. There is some discussion of maintaining the ‘authenticity’ of peer support through its separation from formal services (National Collaborating Centre for Mental Health et al., 2020), and stakeholders were interested in determining whether peer support was led by medical or community providers. However, whilst in many studies peers led the peer support sessions, it was not evident who had initiated or organised the peer support intervention.

Due to resource limitations, data for impact evaluations included within SRs was only extracted from outcomes presented in the relevant SR. However, as the methodology of included SRs may have been slightly different to those of the EGM, extracted data on these impact evaluations is more limited than data extracted directly from RCTs and EEs. For this reason, it is possible that some relevant data may not have been extracted in relation to these studies.

There was also a lack of clarity in the reporting of time frames for follow‐up. Some studies reported outcomes from baseline, others from the end of the intervention. This creates limitations for users looking for evidence regarding the effectiveness of peer support over different timeframes, as peer support interventions included in the EGM ranged in length from one‐off sessions to those lasting several years.

Finally, it remains unclear whether the gaps in the EGM are due to a lack of peer support interventions occurring in these contexts, and among groups with these health and social care needs. It may be that these gaps reflect an absence of recent effectiveness research in these areas.

### 6.5 Stakeholder engagement throughout the EGM process

The only change to our stakeholder engagement during the EGM process occurred after initial conversations with stakeholders involved in policy and practice highlighted the value of consulting users of peer support, due to the insight their experience could bring to, and as potential users of, the map. As a result, we recruited a PPI group with experience of using peer support and held a workshop in which we trialled the use of a draft version on the EGM.

## 7 AUTHORS' CONCLUSIONS

This EGM provides a comprehensive and up‐to‐date overview of the evidence currently available on the effectiveness of peer support interventions in health and social care. Whilst it does not tell us what the evidence says, it allows the user to identify whether and what evidence exists relating to their specific interest in peer support interventions and their areas of practice. There are some clear areas of evidence concentration which could be used in policy and practice. There are also clear gaps in a number of areas, which may prompt extension of the use of peer support for different needs, or in terms of methods of delivery that have not yet been tried, or indicate the need for further research.

### 7.1 Implications for research

RCTs shown individually in this EGM are those not included in the published SRs already on the map. Areas of the map with high numbers of RCTs therefore indicate the potential for systematic reviews on these topics:

- The effectiveness of peer‐led case management and health service liaison as a type of peer support.
- The effectiveness of peer support for populations with chronic mental health difficulties and vulnerable populations.

However, it is also evident from this EGM that there is a lack of research relating to some aspects of peer support which are essential for those commissioning and delivering these services. Impact evaluations, particularly RCTs, are needed on:

- The cost‐effectiveness of peer support compared to other forms of support and/or usual care. Also, research on outcomes beyond physical and mental health, to aid understanding of the mechanisms by which peer support leads to health outcomes.
- The effectiveness of peer support in different geographic locations with different health care systems and contexts.
- The effectiveness of online peer support. Delivering health and social care during the pandemic has led to many adaptations and innovations, such as the provision of services online. This has the potential to increase accessibility, for example, for those with long‐term health conditions who may benefit most from peer support.

Research included in the EGM suggests a need for clarity in the reporting of research. Clear reporting of time frames for follow‐up is particularly important to allow assessment of the sustainability of programmes and outcomes. Studies of peer support should also provide more information on how and by whom the intervention is organised, as well as aspects such as the location of the intervention. Similarly, clearer reporting within SRs would have given greater confidence in the results of these studies.

### 7.2 Implications for policy and practice

- Concentrations of evidence on health‐related outcomes of peer support and populations with chronic health conditions provide a source of information for those looking to commission or provide peer support.
- Integrating into multidisciplinary teams and having an unclear role have been identified as challenges for peer supporters (National Collaborating Centre for Mental Health et al., 2020). Evidence was found in impact evaluations (RCTs and EEs) on the delivery of peer support led by peers who were working with care professionals. These studies could inform policy and practice, particularly as some related to particular types of peer support such as case management and practical support. More evidence is needed, however, on co‐facilitated peer support.

## CONTRIBUTIONS OF AUTHORS

AP led the development of the interactive EGM and strategic planning for drafting the report and refining map categories. SDB, NS, AB, RA, and JTC contributed to the development of the interactive EGM.

AP and SDB carried out screening, data extraction and quality appraisal. RA carried out screening, data extraction quality appraisal of economic evaluations. NS and AB supported screening, data extraction and quality appraisal.

NS and AB designed and ran the search strategies, carried out citation chasing and managed the bibliographic libraries.

SDB led on the drafting, assembly and formatting of the final report. AP, SDB, NS, AB, RA drafted sections of the report and read, provided feedback on, edited and approved the final version of the report. JTC read, provided feedback on, edited and approved the final version of the report.

JTC provided overall project management. RA and JTC contributed to the scoping process, refining of research questions and development of the protocol in collaboration with the protocol authorship team. SDB and AP led on stakeholder engagement/PPI, with support from RA and JTC.

- **Content:**
  All authors have experience of health service and social care research. Although no authors have direct experience in peer support, stakeholders with expertise were involved throughout the development of the EGM. Additionally, authors had topic expertise relevant to studies included in the EGM, including economic evaluations (RA), mental health and mapping health service provision (AP), and care for older adults (JTC).
- **EGM methods:**
  JTC has previously worked on evidence gaps maps. All authors have prior experience in systematic reviews and are proficient in carrying out the various stages required to produce an EGM, including eligibility screening, quality assessment and data extraction.
- **Information retrieval:**
  AB and NS both have training and extensive experience in designing and implementing search strategies.

## DECLARATIONS OF INTEREST

Jo Thompson Coon is a member of the NIHR Health Technology Assessment General Funding Committee and Co‐chair and Editor of the Campbell Ageing Co‐ordinating Group which receives occasional editorial support from the Campbell Social Welfare Co‐ordinating Group. Until July 2019, Rob Anderson was a member of the NIHR Health Services and Delivery Research (Researcher‐Led) Prioritisation Committee. Drs Thompson Coon and Anderson also report grants from NIHR HS&DR grant: Project no. 16/47/22 during the conduct of the study. They and the Department for Health and Social Care are the main policy customer for this systematic review and report. All other authors report no conflict of interests.

## PLANS FOR UPDATING THE EGM

There are no current plans to update this EGM. However, the authors will consider updating the EGM in the future if relevant funding is available.

## DIFFERENCES BETWEEN PROTOCOL AND REVIEW

The only difference in the methods we ultimately used, from those described in the protocol, was to recruit a PPI group with experience of using peer support rather than consult the NIHR PenARC Public Engagement Group (PenPEG) regarding the map.

We did not make any post‐hoc decisions regarding eligibility but as planned in the protocol, we clarified the application of inclusion and exclusion criteria. Similarly, as described in the protocol, for the stage 2 search for impact evaluations, we specified a 2015 cut‐off date for inclusion in the EGM after full screening and as a team decided to list pre‐2015 studies in Supporting Information: Appendix 6.

## SOURCES OF SUPPORT

**Internal sources**

• No sources of support provided

**External sources**

• NIHR HS&DR (Award ID NIHR130538)

## Supporting information

Supporting information.

Supporting information.

Supporting information.
