# Supplementary material for: What is the volume, diversity and nature of recent, robust evidence for the use of peer support in health and social care? An evidence and gap map
Source: Campbell Syst Rev. 2022 Jul 26;18(3):e1264. doi: 10.1002/cl2.1264 (PMC9316011; doi:10.1002/cl2.1264)
Supplement: Supplementary file 1 — Supporting information. [file CL2-18-e1264-s001.docx]

# Appendices

## 1 Glossary

**Glossary**

Glossary of key terms, categories (interventions, outcomes) and filters included in the Evidence and Gap Map. Please note that map category coding reflects information reported in the associated publication (and linked publications, where relevant). Where information was implied, but not stated, this was not coded onto the map.

**KEY TERMS**

| **Term** | **Definition** |
| --- | --- |
| Evidence and gap map (EGM) | An Evidence and Gap Map (EGM) is an interactive tool, designed to provide visual presentations of the availability of rigorous evidence for a particular topic, theme, or policy area. EGMs commonly include systematic reviews and evaluations of impact on ‘what works’ in relation to a specified topic. Derived from a systematic and pre-defined search of available evidence, EGMs consist of a matrix of intervention categories (rows) and outcome domain (columns) placed into an intuitive graphical display. Navigating the map reveals areas for which there is strong, weak, or non-existent evidence available on the effect of interventions. In addition, filters may be available, for example for the type of study, or population characteristics. It is important to note that while EGMs show what evidence is available, they do not comment on what the evidence says. |
| Peer support | Whilst there are many different definitions of peer support, for the purposes of this EGM, we employed the one used within the Nesta and National Voices 2015 report:  *“Peer support involves people drawing on shared personal experience to provide knowledge, social interaction, emotional assistance or practical help to each other, often in a way that is mutually beneficial”*  In addition, we set the condition that there must be an identifiable peer support role, ongoing and formalised to include at least one of the following methods:   - they have received training to fulfil the peer support role; - they receive ongoing support to fulfil the peer support role; - they are paid or have a contract to fulfil the peer support role. |
| Systematic review | A study which collects and synthesises all of the research available on a topic in order to answer a specific question. Systematic reviews seek to minimise bias by setting out their methods in advance, defining the types of study that will be included and methods that will be used to analyse the data for example. |
| Impact evaluation | A study investigating the change in an outcome resulting from an intervention. Different study designs, such as RCTs, can be used to do this. |
| Randomised Controlled Trial | An experimental study in which people are randomly allocated into different groups and each group receives a different intervention. |
| Economic evaluation | A study which looks at the cost of delivering an intervention. This EGM includes economic evaluations, which compare the costs and outcomes of an intervention, as well as broader economic studies. |

**INTERVENTIONS (type of peer support initiative)**

| **Category** | **Sub-category** | **Explanatory notes** |
| --- | --- | --- |
| Description of peer support initiative | Case management, health service liaison | Helping participant to make contact with available health and social care support. |
|  | Education, coaching, mentoring | Provision of information, education, training, mentoring and/or coaching. |
|  | Practical support for health behaviours | Practical help with health behaviours such as exercising or learning to monitor blood glucose levels. |
|  | Psychological, emotional, wellbeing support | Mental health support, mindfulness, wellbeing, and quality of life interventions. |
|  | Self-care, self-management | A focus on self-management, self-care and goal setting. |
|  | Social, community | Social or community input. Aiming to help build relationships and/or support social interaction. |
|  | Not clearly defined | NA |
| Support structure for peers | Trained | NA |
|  | Paid or with contract | NA |
|  | Receive ongoing support | Supervision or additional training/mentoring provided for the peer. |
|  | Not specified | NA |
| Structure of meetings | One-to-one | NA |
|  | In groups | NA |
| Method of contact | In person | NA |
|  | Telephone | NA |
|  | Online | NA |
|  | Not specified | NA |
| Who facilitates intervention | Peers facilitate, not clear whether or how professionals involved | Peers facilitate. Not clear whether there are clinicians also involved. |
|  | Co-facilitated by peers and professionals | Peers and professionals facilitate together. |
|  | Led by peers, working with professionals | Peers lead delivery of the intervention, working with professionals. |
|  | Led by professionals, working with peers | Professionals lead delivery of the intervention, working with peers. |
|  | Not clearly defined | NA |
| Main focus of research | Focus on peer support | NA |
|  | Focus not on peer support alone | Peer support is part of a complex intervention, or one of multiple intervention arms. Effects of peer support are identifiable. |
| Duration of intervention | One-off or ad-hoc | NA |
|  | Up to 3 months | NA |
|  | Over 3, up to 6 months | NA |
|  | Over 6, up to 12 months | NA |
|  | Over 12 months | NA |
|  | Not specified | NA |
| Location of intervention | Own home | NA |
|  | Medical setting | NA |
|  | Community or social location | NA |
|  | Not specified | NA |

**OUTCOMES**

| **Category** | **Sub-category** | **Explanatory notes** |
| --- | --- | --- |
| Health related indicators  Outcomes measuring physical or mental health status | Physical health | Indicator of physical health. |
|  | Mental health | Indicator of mental health. |
| Self-regulation  Skills for self-management, and practising behaviours linked to health and social outcomes | Self-management | Learning and practicing skills to enable management of health and social needs on a day-to-day basis. |
|  | Health behaviours | Health service utilisation (for example, appointment attendance, treatment engagement). Repeated behaviours that influence social and/or health outcomes. |
|  | Addiction recovery | Outcomes related to addictive behaviours; such as smoking cessation, or changes in substance abuse. |
| Supporting self-regulation  Skills, understanding and attitudes potentially linked to self-regulation and management of health and social needs. | Self-efficacy | Self-efficacy, empowerment. Self-advocacy. Self-esteem. Measures that quantify mental state and attitudes that are likely to translate into health behaviours. |
|  | Knowledge, understanding | Knowledge, understanding, information held about own health condition, how to meet own social needs etc. |
| Wellbeing, social connectedness | Wellbeing, quality of life | Measures of quality of life, balance, wellbeing, positive lifestyle. |
|  | Social support and relationships | Perceived social connectedness, social support. Relationships. |
| Cost-effectiveness, service use | Cost effectiveness | Comparative analysis of two or more alternative interventions in terms of health, social and economic consequences. |
|  | Service use | Measure of use of any health, social or other services. |
|  | Employment status | Measure of employment status. |
| Experience of peer support | Experience of support | Experience of receiving or providing peer support. |
|  | Peer outcomes | Outcomes for peer supporters. |

**FILTERS**

| **Category** | **Sub-category** | **Explanatory notes** |
| --- | --- | --- |
| Study type | Economic evaluation (EE) | Effectiveness study looking at the economic impact of peer support interventions. |
|  | Randomised controlled trial (RCT) | Effectiveness study looking at the effectiveness of peer support interventions. |
|  | Systematic review (SR) | A formalised (systematic) review of the available evidence on a specific question related to the effectiveness of peer support. May include RCTs, other effectiveness studies and/or other research. |
| Population characteristics | Mental health difficulties/needs (acute) | A short-term difficulty or need or a crisis related to chronic difficulties. |
|  | Mental health difficulties/needs (chronic) | Chronic needs or difficulties. |
|  | Physical health difficulties/needs (acute) | A short-term difficulty or need or a crisis related to chronic difficulties. |
|  | Physical health difficulties/needs (chronic) | Chronic needs or difficulties. |
|  | Addiction difficulties | Can be with addition or in recovery. Includes alcohol and drugs. |
|  | Parents, carers | Parents or carers of people who have health or social care needs/difficulties. |
|  | Vulnerable (e.g. experienced trauma, homelessness, criminal justice contact) | People identified as vulnerable, including people who have experienced homelessness, trauma, and/or contact with the criminal justice system. , those who have experienced trauma, in criminal justice system. |
| Population age When range not given, reflects mean age, plus/minus standard deviation. | Includes young people 18-25 | NA |
|  | Includes adults 26-64 | NA |
|  | Includes older adults 65+ | NA |
|  | Not clearly defined | NA |
| Study location/s by type of health system  Following stakeholder feedback, these categories were developed to indicate  organisation of health systems. | United Kingdom (UK) | Location of EEs and/or of studies included in  SRs. |
|  | Europe (other than UK) | Location of EEs and/or of studies included in  SRs. |
|  | USA or Canada | Location of EEs and/or of studies included in  SRs. |
|  | Australia or New Zealand | Location of EEs and/or of studies included in  SRs. |
|  | Other / not specified | Location of EEs and/or of studies included in  SRs. |
| Assessment timeframe  Where possible this reflects time from intervention end. However, in some studies, reflects time since baseline. | At intervention end | NA |
|  | Up to 3 months | NA |
|  | Over 3, up to 6 months | NA |
|  | Over 6, up to 12 months | NA |
|  | Over 12, up to 24 months | NA |
|  | Over 24 months, up to 5 years | NA |
|  | Over 5 years, up to 10 years | NA |
|  | Lifetime | Relevant for economic evaluations. |
|  | Not specified | NA |
| Quality appraisal: SR overall confidence in quality of results (AMSTAR-2) | High overall confidence | Higher overall confidence. |
|  | Moderate overall confidence | Higher overall confidence. |
|  | Low overall confidence | Lower overall confidence. |
|  | Critically low overall confidence | Lower overall confidence. |
| Quality appraisal: RCT summary assessment of risk of bias (RoB) | Low risk of bias | Higher overall confidence. |
|  | Unclear risk of bias | Lower overall confidence. |
|  | High risk of bias | Lower overall confidence. |
| Quality appraisal: EE overall quality assessment (CHEC-list) | High overall quality | Higher overall confidence. |
|  | Medium overall quality | Higher overall confidence. |
|  | Low overall quality | Lower overall confidence. |
| Study year | 2015 | NA |
|  | 2016 | NA |
|  | 2018 | NA |
|  | 2019 | NA |
|  | 2020 | NA |
|  | 2021 | NA |

## 2 Inclusion and exclusion criteria

|  | **Include** | **Exclude** |
| --- | --- | --- |
| Literature type | Published journal articles  Theses  Ongoing SR and RCT protocols | Commentary or conceptual papers  Editorial  Conference proceedings  Case studies |
| Study design | All study designs must include quantitative data  Permissible study designs included:   - SRs - Meta-analysis/meta-regressions - RCTs which have not been included in a recent, high quality SR - EEs, including: cost-effectiveness analyses, cost-utility analyses, cost-benefit analyses, comparative costing studies | Studies reporting only qualitative data were excluded regardless of design  Literature reviews  Pilot RCTs, or feasibility RCTs without power to detect differences between groups  Mediator/moderator analyses of RCTs, as focus is not effectiveness  Non-randomised quantitative studies e.g. quasi-experimental studies |
| Date | SRs published after 2015  RCTs and EEs published in any year; data was only extracted for those published after 2015 i.e. those not included in a recent SR | SRs published before 2015, to avoid duplication of previous reviews (Mind, 2013; Nesta & National Voices, 2015) |
| Language | Studies reported in English | Studies not reported in English, due to study team expertise and time and resources constraints |
| Location | Conducted in high-income countries as defined by the World Bank (World Bank, 2021), to focus on evidence in contexts, e.g. in terms of health care systems or available resources, most relevant to intended users of the map | Studies conducted in low or middle income countries, except SRs covering both high and low or middle income countries where results of studies from high income countries could be distinguished |
| Population | Users of adult services with a defined health and/or social care need (include both users and carers)  Users of care homes or other social care services  These criteria focused the EGM on populations most relevant to the intended users of the map.  Adults 18+. We included studies on young people if the majority of participants were over 18 and studies of adult carers for children with a defined health need, if the study included outcomes for these adults. | We excluded populations identified as ‘at risk’ or engaging in ‘risky behaviour’, unless they had a current defined health or social care need and were using services. This means that some important preventative interventions e.g. health screening, healthy eating, were only included if they focussed on a population which already had identifiable health needs.  Studies where the majority of participants were below 18 years of age. |
| Comparators | Any comparator was eligible for inclusion. Examples included wait-list control, treatment as usual, education. | Studies that did not have a comparator which contrasted with the peer support intervention e.g. comparison of two forms of peer support, as this would mean the effectiveness of PS could not be identified. |
| Interventions | Intervention must focus on peer support as unique element, involving delivery of peer support as defined below:  *People drawing on shared personal experience (to provide knowledge, social interaction, emotional assistance or practical help to each other, often in a way that is mutually beneficial).*  Delivered by identifiable peer supporter/s, with the same or similar health condition(s) to the peer, who (one or more):   - have received training - receive ongoing support - are paid or have a contract   Peer support delivered in any format (such as face-to-face, online, group, individual, mixed modes etc.) and with any content, delivered by paid or unpaid peer supporters  We included studies where peers were a mix of those with a health condition and those caring for someone with a health condition if the majority had the condition or if we were unable | Interventions not meeting the definition or described poorly enough to preclude assessment of intervention type.  Studies were excluded where peer support was part of a multi-component intervention e.g. along with strength training/motivational videos, so its effects on outcomes could not be identified  Peer support delivered outside a health or social care context, e.g. in education.  In line with our definition of peer support, we did not include ‘peers’ where this meant from same community with experience of caring for person with condition. This excluded some non-disease specific peer support programmes, such as the expert patient programme, where patients with experience of living with a LTC (peers) deliver a self-management course to people living with LTCs. We only included studies where peers had experience of same specific health condition.  Interventions where participants are being trained as peer supporters |
| Setting | Any setting e.g. home, community, health or social care | Studies were only excluded on setting where this meant the effects of peer support on outcomes could not identified e.g. in AA recovery homes peer support is only one component of the support provided to residents |
| Outcomes | We included any outcomes associated with effectiveness or cost-effectiveness of peer support interventions (and related to health and social care needs):   - Capabilities and intentions (self-efficacy) - Health related behaviours (healthy eating, exercise, medication adherence) - Service use levels (attendance at scheduled events, use of emergency services) - Wellbeing and quality of life - Health outcomes (mental, physical health) - Social care outcomes (need for adult social care)   This included outcomes that might not benefit the participant but do benefit the health of others e.g. transmission risk behaviour among HIV-positive participants | Outcomes that only measured acceptability or feedback, as we were interestred in the effectiveness of the intervention  Outcomes only related to delivering peer support, not receiving it |

## 3 Search strategies

**Stage 1 search strategies:**

**Ovid MEDLINE (ALL 1946 to September 29, 2020)**

Search completed: 1/10/2020

1 (peer* adj3 (administer* or adviser* or advisor* or advocate* or coach* or co-facilitat* or cofacilitat* or consultant* or counsel* or deliver* or educator* or expert* or facilitator* or group* or helper* or instructor* or leader* or led or listener* or mentor* or navigator* or network* or program* or provider* or specialist* or support* or trainer* or trained or tutor* or worker*)).tw. (17015)

2 ("peer-based" or "peer based").tw. (423)

3 "peer to peer".tw. (1394)

4 peer group/ (20749)

5 (buddy or buddies or befriend*).tw. (1078)

6 ("service user*" adj1 (involv* or led or run)).tw. (381)

7 (consumer* adj (deliver* or provider* or led or run)).tw. (329)

8 ((lay or voluntary or volunteer) adj2 (adviser* or advisor* or advocate* or coach* or consultant* or counsel* or educator* or expert* or facilitator* or helper* or instructor* or leader* or led or listener* or mentor* or provider* or specialist* or support* or trainer* or trained or tutor* or worker*)).tw. (5047)

9 "lay health care worker*".ti,ab. (25)

10 (("social support" adj5 intervention*) or "support group*").tw. (8568)

11 ("support network*" or "mutual aid" or "mutual support").tw. (4051)

12 (expert adj patient*).tw. (262)

13 "shared experience".tw. (365)

14 *Self-Help Groups/ (5250)

15 or/1-14 (54295) [peer support search terms]

16 ((systematic* or systematized or integrative or mapping or rapid or scoping) adj3 review*).tw. (207271)

17 ((evidence or interpretive or meta or quantitative) adj1 synthes?s).tw. (7427)

18 ((evidence adj2 map) or "systematic map").tw. (443)

19 ("mixed method*" adj3 review*).tw. (572)

20 ("meta-analys?s" or metaanalys?s).tw. (179152)

21 systematic review.pt. (135796)

22 meta-analysis.pt. (120248)

23 (cost* adj3 review*).tw. (3218)

24 (data adj extraction).ab. (22169)

25 (narrative adj (review* or synthes?s)).tw. (14659)

26 or/16-25 (348857) [systematic review search]

27 15 and 26 (1851)

28 limit 27 to yr="2015 -Current" (1123)

**Ovid APA PsycINFO <1806 to September Week 3 2020>**

Search completed: 1/10/2020

1 (peer* adj3 (administer* or adviser* or advisor* or advocate* or coach* or co-facilitat* or cofacilitat* or consultant* or counsel* or deliver* or educator* or expert* or facilitator* or group* or helper* or instructor* or leader* or led or listener* or mentor* or navigator* or network* or program* or provider* or specialist* or support* or trainer* or trained or tutor* or worker*)).tw. (23917)

2 ("peer-based" or "peer based").tw. (468)

3 "peer to peer".tw. (1557)

4 peers/ (12950)

5 peer relations/ (16335)

6 peer counseling/ (1128)

7 peer tutoring/ (1527)

8 (buddy or buddies or befriend*).tw. (1158)

9 ("service user*" adj1 (involv* or led or run)).tw. (515)

10 (consumer* adj (deliver* or provider* or led or run)).ti,ab. (341)

11 ((lay or voluntary or volunteer) adj2 (adviser* or advisor* or advocate* or coach* or consultant* or counsel* or educator* or expert* or facilitator* or helper* or instructor* or leader* or led or listener* or mentor* or provider* or specialist* or support* or trainer* or trained or tutor* or worker*)).tw. (3446)

12 "lay health care worker*".tw. (10)

13 (("social support" adj5 intervention*) or "support group*").tw. (9200)

14 ("support network*" or "mutual aid" or "mutual support").ti,ab. (5618)

15 (expert adj patient*).ti,ab. (99)

16 "shared experience".tw. (732)

17 *Support Groups/ (3443)

18 or/1-17 (65444) [peer support search terms]

19 ((systematic* or systematized or integrative or mapping or rapid or scoping) adj3 review*).tw. (39781)

20 ((evidence or interpretive or meta or quantitative) adj1 synthes?s).tw. (1814)

21 ((evidence adj2 map) or "systematic map").tw. (84)

22 ("mixed method*" adj3 review*).tw. (231)

23 ("meta-analys?s" or metaanalys?s).tw. (36306)

24 "systematic review"/ (442)

25 (cost* adj3 review*).tw. (444)

26 (data adj extraction).ab. (2031)

27 (narrative adj (review* or synthes?s)).tw. (3316)

28 or/19-27 (69529) [systematic review search]

29 18 and 28 (1030)

30 limit 29 to yr="2015 -Current" (581)

**Ovid Embase (<1974 to 2020 September 30>)**

Search completed: 1/10/2020

1 (peer* adj3 (administer* or adviser* or advisor* or advocate* or coach* or co-facilitat* or cofacilitat* or consultant* or counsel* or deliver* or educator* or expert* or facilitator* or group* or helper* or instructor* or leader* or led or listener* or mentor* or navigator* or network* or program* or provider* or specialist* or support* or trainer* or trained or tutor* or worker*)).tw. (22606)

2 ("peer-based" or "peer based").tw. (526)

3 "peer to peer".tw. (2016)

4 peer group/ (23950)

5 (buddy or buddies or befriend*).tw. (1541)

6 ("service user*" adj1 (involv* or led or run)).tw. (486)

7 (consumer* adj (deliver* or provider* or led or run)).tw. (391)

8 ((lay or voluntary or volunteer) adj2 (adviser* or advisor* or advocate* or coach* or consultant* or counsel* or educator* or expert* or facilitator* or helper* or instructor* or leader* or led or listener* or mentor* or provider* or specialist* or support* or trainer* or trained or tutor* or worker*)).tw. (6235)

9 "lay health care worker*".tw. (27)

10 (("social support" adj5 intervention*) or "support group*").tw. (12912)

11 ("support network*" or "mutual aid" or "mutual support").ti,ab. (5294)

12 (expert adj patient*).ti,ab. (473)

13 "shared experience".tw. (485)

14 exp *support group/ (1622)

15 or/1-14 (64023) [peer support search terms]

16 ((systematic* or systematized or integrative or mapping or rapid or scoping) adj3 review*).tw. (257388)

17 ((evidence or interpretive or meta or quantitative) adj1 synthes?s).tw. (8241)

18 ((evidence adj2 map) or "systematic map").tw. (491)

19 ("mixed method*" adj3 review*).tw. (623)

20 ("meta-analys?s" or metaanalys?s).tw. (234317)

21 "systematic review"/ (263121)

22 exp meta analysis/ (197766)

23 (cost* adj3 review*).tw. (4585)

24 (data adj extraction).ab. (27248)

25 (narrative adj (review* or synthes?s)).tw. (15736)

26 or/16-25 (501029) [systematic review search]

27 15 and 26 (2387)

28 limit 27 to yr="2015 -Current" (1484)

**Cochrane Database of Systematic Reviews (Wiley)**

Search completed: 1/10/2020

#1 (peer* near/3 (administer* or adviser* or advisor* or advocate* or coach* or co-facilitat* or cofacilitat* or consultant* or counsel* or deliver* or educator* or expert* or facilitator* or group* or helper* or instructor* or leader* or led or listener* or mentor* or navigator* or network* or program* or provider* or specialist* or support* or trainer* or trained or tutor* or worker*)):ti,ab,kw

#2 ("peer-based" or "peer based"):ti,ab,kw

#3 ("peer to peer"):ti,ab,kw

#4 MeSH descriptor: [Peer Group] explode all trees

#5 (buddy or buddies or befriend*):ti,ab,kw

#6 ("service user*" next (involv* or led or run)):ti,ab,kw

#7 (consumer* next (deliver* or provider* or led or run)):ti,ab,kw

#8 ((lay or voluntary or volunteer) near/2 (adviser* or advisor* or advocate* or coach* or consultant* or counsel* or educator* or expert* or facilitator* or helper* or instructor* or leader* or led or listener* or mentor* or provider* or specialist* or support* or trainer* or trained or tutor* or worker*)):ti,ab,kw

#9 ("lay health care worker" or "lay health care workers"):ti,ab,kw

#10 (("social support" near/5 intervention*) or "support group" or "support groups"):ti,ab,kw

#11 ("support network" or "support networks" or "mutual aid" or "mutual support"):ti,ab,kw

#12 (expert next patient*):ti,ab,kw

#13 (shared next experience):ti,ab,kw

#14 MeSH descriptor: [Self-Help Groups] explode all trees

#15 #1 or #2 or #3 or #4 or #5 or #6 or #7 or #8 or #9 or #10 or #11 or #12 or #13 or #14

**EBSCOhost CINAHL Complete**

Search completed: 1/10/2020

S25 S14 AND S24 877

S24 (S15 OR S16 OR S17 OR S18 OR S19 OR S20 OR S21 OR S22 OR S23) 185,122

S23 AB "data extraction" 8,161

S22 TI (cost* N2 review*) OR (narrative N0 (review* or synthes?s)) OR AB (cost* N2 review*) OR (narrative N0 (review* or synthes?s)) 8,377

S21 (MH "Meta Analysis") 48,491

S20 (MH "Systematic Review") 85,635

S19 TI ( ("meta-analys?s" or metaanalys?s) ) OR AB ( ("meta-analys?s" or metaanalys?s) ) 71,403

S18 TI ("mixed method*" N2 review*) OR AB ("mixed method*" N2 review*) 364

S17 TI ( (evidence N1 map) or "systematic map" ) OR AB ( (evidence N1 map) or "systematic map" ) ) 170

S16 TI ( ((evidence or interpretive or meta or quantitative) N1 synthes?s) ) OR AB ( ((evidence or interpretive or meta or quantitative) N1 synthes?s) ) 3,626

S15 TI ( ((systematic* or systematized or integrative or mapping or rapid or scoping) N2 review*) ) OR AB ( ((systematic* or systematized or integrative or mapping or rapid or scoping) N2 review*) ) 112,687

S14 S1 OR S2 OR S3 OR S4 OR S5 OR S6 OR S7 OR S8 OR S9 OR S10 OR S11 OR S12 OR S13 39,279

S13 (MM "Support Groups") 5,426

S12 TI ("expert patient*" OR "shared experience") OR AB ("expert patient*" OR "shared experience") 533

S11 AB ( ("support network*" or "mutual aid" or "mutual support") ) OR TI ( ("support network*" or "mutual aid" or "mutual support") ) 3,452

S10 AB ( ("social support" N4 intervention*) or "support group*" ) OR TI (("social support" N4 intervention*) or "support group*" ) 6,741

S9 AB ( ("lay health care worker*") ) OR TI ( ("lay health care worker*") ) 17

S8 AB ( ((lay or voluntary or volunteer) N1 (adviser* or advisor* or advocate* or coach* or consultant* or counsel* or educator* or expert* or facilitator* or helper* or instructor* or leader* or led or listener* or mentor* or provider* or specialist* or support* or trainer* or trained or tutor* or worker*)) ) OR TI ( ((lay or voluntary or volunteer) N1 (adviser* or advisor* or advocate* or coach* or consultant* or counsel* or educator* or expert* or facilitator* or helper* or instructor* or leader* or led or listener* or mentor* or provider* or specialist* or support* or trainer* or trained or tutor* or worker*)) ) 3,343

S7 AB ( (consumer* N0 (deliver* or provider* or led or run)) ) OR TI ( (consumer* N0 (deliver* or provider* or led or run)) ) 389

S6 AB ( ("service user*" N0 (involv* or led or run)) ) OR TI ( ("service user*" N0 (involv* or led or run)) ) 600

S5 AB ( (buddy or buddies or befriend*) ) OR TI ( (buddy or buddies or befriend*) ) 978

S4 (MH "Peer Group") 13,308

S3 TI "peer to peer" OR AB "peer to peer" 900

S2 TI ( ("peer-based" or "peer based") ) OR AB ( ("peer-based" or "peer based") ) 300

S1 TI ( (peer* N2 (administer* or adviser* or advisor* or advocate* or coach* or co-facilitat* or cofacilitat* or consultant* or counsel* or deliver* or educator* or expert* or facilitator* or group* or helper* or instructor* or leader* or led or listener* or mentor* or navigator* or network* or program* or provider* or specialist* or support* or trainer* or trained or tutor* or worker*)) ) OR AB ( (peer* N2 (administer* or adviser* or advisor* or advocate* or coach* or co-facilitat* or cofacilitat* or consultant* or counsel* or deliver* or educator* or expert* or facilitator* or group* or helper* or instructor* or leader* or led or listener* or mentor* or navigator* or network* or program* or provider* or specialist* or support* or trainer* or trained or tutor* or worker*)) ) 12,159

**ProQuest Applied Social Sciences Index & Abstracts (ASSIA)**

Search completed: 5/10/2020

Note: ProQuest would not allow combinations of required searches, so 3 separate searches were completed with all results exported to an EndNote library and de-duplicated. (Total 188 records from ASSIA searches after de-duplication.)

*Search #1: limited to 2015 – 5^th^ October 2020 (41 records)*

((ab(((systematic* OR systematized OR integrative OR mapping OR rapid OR scoping) NEAR/2 review*)) OR ti(((systematic* OR systematized OR integrative OR mapping OR rapid OR scoping) NEAR/2 review*))) OR (ab(((evidence OR interpretive OR meta OR quantitative) NEAR/1 synthes?s)) OR ti(((evidence OR interpretive OR meta OR quantitative) NEAR/1 synthes?s))) OR (ab(((evidence NEAR/1 map) OR "systematic map")) OR ti(((evidence NEAR/1 map) OR "systematic map"))) OR (ab(("mixed method*" NEAR/2 review*)) OR ti(("mixed method*" NEAR/2 review*))) OR (ab(("meta-analys?s" OR metaanalys?s)) OR ti(("meta-analys?s" OR metaanalys?s))) OR (MAINSUBJECT.EXACT("Systematic reviews") OR MAINSUBJECT.EXACT("Meta-analysis")) OR (ab((cost* NEAR/2 review*)) OR ti((cost* NEAR/2 review*))) OR (ab(("data extraction")) OR ti(("data extraction"))) OR (ab((narrative NEAR/1 (review* OR synthes?s))) OR ti((narrative NEAR/1 (review* OR synthes?s))))) AND ((ab("lay health care worker*") OR ti("lay health care worker*")) OR (ab((("social support" NEAR/4 intervention*) OR ("support group" OR "support groups"))) OR ti((("social support" NEAR/4 intervention*) OR ("support group" OR "support groups")))) OR (ab("expert patient*") OR ti("expert patient*")) OR (ab("shared experience") OR ti("shared experience")))

*Search #2: limited to 2015 – 5^th^ October 2020 (56 records)*

((ab(((systematic* OR systematized OR integrative OR mapping OR rapid OR scoping) NEAR/2 review*)) OR ti(((systematic* OR systematized OR integrative OR mapping OR rapid OR scoping) NEAR/2 review*))) OR (ab(((evidence OR interpretive OR meta OR quantitative) NEAR/1 synthes?s)) OR ti(((evidence OR interpretive OR meta OR quantitative) NEAR/1 synthes?s))) OR (ab(((evidence NEAR/1 map) OR "systematic map")) OR ti(((evidence NEAR/1 map) OR "systematic map"))) OR (ab(("mixed method*" NEAR/2 review*)) OR ti(("mixed method*" NEAR/2 review*))) OR (ab(("meta-analys?s" OR metaanalys?s)) OR ti(("meta-analys?s" OR metaanalys?s))) OR (MAINSUBJECT.EXACT("Systematic reviews") OR MAINSUBJECT.EXACT("Meta-analysis")) OR (ab((cost* NEAR/2 review*)) OR ti((cost* NEAR/2 review*))) OR (ab(("data extraction")) OR ti(("data extraction"))) OR (ab((narrative NEAR/1 (review* OR synthes?s))) OR ti((narrative NEAR/1 (review* OR synthes?s))))) AND ((ab("peer-based" OR "peer based") OR ti("peer-based" OR "peer based")) OR (ab("peer to peer") OR ti("peer to peer")) OR (ab(buddy OR buddies OR befriend*) OR ti(buddy OR buddies OR befriend*)) OR (ab((("service user" OR "service users") NEAR/4 (involv* OR led OR run))) OR ti((("service user" OR "service users") NEAR/4 (involv* OR led OR run)))) OR (ab((consumer* NEAR/4 (deliver* OR provider* OR led OR run))) OR ti((consumer* NEAR/4 (deliver* OR provider* OR led OR run)))) OR (ab(((lay OR voluntary OR volunteer) NEAR/1 (adviser* OR advisor* OR advocate* OR coach* OR consultant* OR counsel* OR educator* OR expert* OR facilitator* OR helper* OR instructor* OR leader* OR led OR listener* OR mentor* OR provider* OR specialist* OR support* OR trainer* OR trained OR tutor* OR worker*))) OR ti(((lay OR voluntary OR volunteer) NEAR/1 (adviser* OR advisor* OR advocate* OR coach* OR consultant* OR counsel* OR educator* OR expert* OR facilitator* OR helper* OR instructor* OR leader* OR led OR listener* OR mentor* OR provider* OR specialist* OR support* OR trainer* OR trained OR tutor* OR worker*)))))

*Search #3: limited to 2015 – 5^th^ October 2020 (107 records)*

(ti((peer* NEAR/2 (administer* OR adviser* OR advisor* OR advocate* OR coach* OR co-facilitat* OR cofacilitat* OR consultant* OR counsel* OR deliver* OR educator* OR expert* OR facilitator* OR group* OR helper* OR instructor* OR leader* OR led OR listener* OR mentor* OR navigator* OR network* OR program* OR provider* OR specialist* OR support* OR trainer* OR trained OR tutor* OR worker*))) OR ab((peer* NEAR/2 (administer* OR adviser* OR advisor* OR advocate* OR coach* OR co-facilitat* OR cofacilitat* OR consultant* OR counsel* OR deliver* OR educator* OR expert* OR facilitator* OR group* OR helper* OR instructor* OR leader* OR led OR listener* OR mentor* OR navigator* OR network* OR program* OR provider* OR specialist* OR support* OR trainer* OR trained OR tutor* OR worker*)))) AND ((ab(((systematic* OR systematized OR integrative OR mapping OR rapid OR scoping) NEAR/2 review*)) OR ti(((systematic* OR systematized OR integrative OR mapping OR rapid OR scoping) NEAR/2 review*))) OR (ab(((evidence OR interpretive OR meta OR quantitative) NEAR/1 synthes?s)) OR ti(((evidence OR interpretive OR meta OR quantitative) NEAR/1 synthes?s))) OR (ab(((evidence NEAR/1 map) OR "systematic map")) OR ti(((evidence NEAR/1 map) OR "systematic map"))) OR (ab(("mixed method*" NEAR/2 review*)) OR ti(("mixed method*" NEAR/2 review*))) OR (ab(("meta-analys?s" OR metaanalys?s)) OR ti(("meta-analys?s" OR metaanalys?s))) OR (MAINSUBJECT.EXACT("Systematic reviews") OR MAINSUBJECT.EXACT("Meta-analysis")) OR (ab((cost* NEAR/2 review*)) OR ti((cost* NEAR/2 review*))) OR (ab(("data extraction")) OR ti(("data extraction"))) OR (ab((narrative NEAR/1 (review* OR synthes?s))) OR ti((narrative NEAR/1 (review* OR synthes?s)))))

**Proquest Dissertations & Theses**

Search completed: 1/10/2020

(ti((peer* NEAR/2 (administer* OR adviser* OR advisor* OR advocate* OR coach* OR co-facilitat* OR cofacilitat* OR consultant* OR counsel* OR deliver* OR educator* OR expert* OR facilitator* OR group* OR helper* OR instructor* OR leader* OR led OR listener* OR mentor* OR navigator* OR network* OR program* OR provider* OR specialist* OR support* OR trainer* OR trained OR tutor* OR worker*))) OR ti(("peer-based" OR "peer based")) OR ti("peer to peer") OR ti((buddy OR buddies OR befriend*)) OR ti((("service user" OR "service users") NEAR/4 (involv* OR led OR run))) OR ti((consumer* NEAR/4 (deliver* OR provider* OR led OR run))) OR ti(((lay OR voluntary OR volunteer) NEAR/1 (adviser* OR advisor* OR advocate* OR coach* OR consultant* OR counsel* OR educator* OR expert* OR facilitator* OR helper* OR instructor* OR leader* OR led OR listener* OR mentor* OR provider* OR specialist* OR support* OR trainer* OR trained OR tutor* OR worker*))) OR ti(("lay health care worker*")) OR ti(("social support" NEAR/4 intervention*) OR ("support group" OR "support groups")) OR ti("expert patient*") OR ti("shared experience") OR mainsubject.Exact("peer relationships" OR "peers") OR mainsubject.Exact("support groups")) AND pd(20150101-20201231)

**Epistemonikos**

<https://www.epistemonikos.org/>

Search completed: 2/10/2020

Note: Epistemonikos would not allow combinations of full search string, so 8 separate searches were completed with all results exported to EndNote and de-duplicated. (Total 332 records from all Epistemonikos searches after de-duplication.)

*Search #1: limited to 2015-2021. No publication type filter applied. 85 records*

Title/Abstract: peer adminster* OR peer adviser* OR peer advisor* OR peer advocate* OR peer coach* OR peer cofacilitator* OR peer co-facilitator* OR peer counsel* OR peer deliver* OR peer educator* OR peer expert* OR peer faciltator* OR peer group* OR peer helper* OR peer instructor* OR peer leader* OR peer led OR peer listener* OR peer mentor* OR peer navigator* OR peer network* OR peer program* OR peer provider* OR peer specialist* OR peer support* OR peer trainer* OR peer trained OR peer worker*

*Search #2: limited to 2015-2021. No publication type filter applied. 23 records*

Title/abstract: "peer-based" OR "peer based"

*Search #3: limited to 2015-2021. No publication type filter applied. 20 records*

Title/abstract: buddy OR buddies OR befriend*

*Search #4: limited to 2015-2021. No publication type filter applied. 55 records*

Title/abstract: lay adviser* OR lay advisor* OR lay advocate* OR lay coach* OR lay consultant* OR lay counsel* OR lay educator* OR lay expert* OR lay facilitator* OR lay helper* OR lay instructor* OR lay leader* OR lay led OR lay listener* OR lay mentor* OR lay provider* OR lay specialist* OR lay support* OR lay trainer* OR lay trained OR lay tutor* OR lay worker*

*Search #5: limited to 2015-2021. No publication type filter applied. 23 records*

Title/abstract: volunteer adviser* OR volunteer advisor* OR volunteer advocate* OR volunteer coach* OR volunteer consultant* OR volunteer counsel* OR volunteer educator* OR volunteer expert* OR volunteer facilitator* OR volunteer helper* OR volunteer instructor* OR volunteer leader* OR volunteer led OR volunteer listener* OR volunteer mentor* OR volunteer provider* OR volunteer specialist* OR volunteer support* OR volunteer trainer* OR volunteer trained OR volunteer tutor* OR volunteer worker*

*Search #6: limited to 2015-2021. No publication type filter applied. 61 records*

Title/abstract: "social support intervention*" or "support group*"

*Search #7: limited to 2015-2021. No publication type filter applied. 66 records*

Title/abstract: "support network*" OR "mutual aid" OR "mutual support" or "shared experience"

*Search #8: limited to 2015-2021. No publication type filter applied*. 5 records

Title/abstract: “expert patient”

**Google Scholar**

Search completed: 26/01/2021 via Publish or Perish (Harzing). Limited to 2015-2021. Total records exported from PoP: 244.

Expert patient AND systematic review (title)

“Peer to peer” AND systematic review (title)

Befriending AND systematic review (title)

Buddy AND systematic review (title)

Buddies AND systematic review (title)

Lay adviser AND systematic r“Peer advisor” AND randomized OR randomised OR RCT eview (title)

Lay advisers AND systematic review (title)

Lay advisor AND systematic review (title)

Lay advisors AND systematic review (title)

Lay advocate AND systematic review (title)

Lay advocates AND systematic review (title)

Lay coach AND systematic review (title)

Lay coaches AND systematic review (title)

Lay coaching AND systematic review (title)

Lay facilitator AND systematic review (title)

Lay facilitators AND systematic review (title)

Lay facilitated AND systematic review (title)

Lay health care worker AND systematic review (title)

Lay healthcare worker AND systematic review (title)

Lay health care workers AND systematic review (title)

Lay healthcare workers AND systematic review (title)

Lay instructor AND systematic review (title)

Lay instructors AND systematic review (title)

Lay leader AND systematic review (title)

Lay leaders AND systematic review (title)

Lay mentor AND systematic review (title)

Lay mentors AND systematic review (title)

Lay support AND systematic review (title)

Lay trainer AND systematic review (title)

Lay trainers AND systematic review (title)

Lay trained AND systematic review (title)

Lay-led AND systematic review (title)

Mutual aid AND systematic review (title)

Mutual support AND systematic review (title)

Peer adviser AND systematic review (title)

Peer advisers AND systematic review (title)

Peer advisor AND systematic review (title)

Peer advisors AND systematic review (title)

Peer advocate AND systematic review (title)

Peer advocates AND systematic review (title)

Peer based AND systematic review (title)

Peer-based AND systematic review (title)

Peer coach AND systematic review (title)

Peer coaches AND systematic review (title)

Peer coaching AND systematic review (title)

Peer counsellor AND systematic review (title)

Peer counsellors AND systematic review (title)

Peer counselor AND systematic review (title)

Peer counselors AND systematic review (title)

Peer delivered AND systematic review (title)

Peer education AND systematic review (title)

Peer educator AND systematic review (title)

Peer educators AND systematic review (title)

Peer expert AND systematic review (title)

Peer facilitated AND systematic review (title)

Peer facilitator AND systematic review (title)

Peer facilitators AND systematic review (title)

Peer instructor AND systematic review (title)

Peer instructors AND systematic review (title)

Peer leader AND systematic review (title)

Peer leaders AND systematic review (title)

Peer led AND systematic review (title)

Peer-led AND systematic review (title)

Peer mediated AND systematic review (title)

Peer mentor AND systematic review (title)

Peer mentors AND systematic review (title)

Peer mentoring AND systematic review (title)

Peer navigator AND systematic review (title)

Peer navigators AND systematic review (title)

Peer support AND systematic review (title)

Peer supported AND systematic review (title)

Peer supporter AND systematic review (title)

Peer supporters AND systematic review (title)

Peer trainer AND systematic review (title)

Peer trainers AND systematic review (title)

Peer trained AND systematic review (title)

Peer tutor AND systematic review (title)

Peer tutors AND systematic review (title)

Peer worker AND systematic review (title)

Peer workers AND systematic review (title)

Social support intervention AND systematic review (title)

Social support interventions AND systematic review (title)

Volunteer counselor AND systematic review (title)

Volunteer counsellor AND systematic review (title)

Volunteer counselors AND systematic review (title)

Volunteer counsellors AND systematic review (title)

Volunteer led AND systematic review (title)

Volunteer-led AND systematic review (title)

**Open Grey**

<http://www.opengrey.eu/>

Search completed: 28/01/2021.

Results screened on OpenGrey.eu. 0 records selected for screening at title/abstract.

Expert patient AND systematic review

“Peer to peer” AND systematic review

Befriending AND systematic review

Buddy AND systematic review

Buddies AND systematic review

Lay adviser AND systematic review

Lay advisers AND systematic review

Lay advisor AND systematic review

Lay advisors AND systematic review

Lay advocate AND systematic review (title)

Lay advocates AND systematic review (title)

Lay coach AND systematic review (title)

Lay coaches AND systematic review (title)

Lay coaching AND systematic review (title)

Lay facilitator AND systematic review (title)

Lay facilitators AND systematic review (title)

Lay facilitated AND systematic review (title)

Lay health care worker AND systematic review (title)

Lay healthcare worker AND systematic review (title)

Lay health care workers AND systematic review (title)

Lay healthcare workers AND systematic review (title)

Lay instructor AND systematic review (title)

Lay instructors AND systematic review (title)

Lay leader AND systematic review (title)

Lay leaders AND systematic review (title)

Lay mentor AND systematic review (title)

Lay mentors AND systematic review (title)

Lay support AND systematic review (title)

Lay trainer AND systematic review (title)

Lay trainers AND systematic review (title)

Lay trained AND systematic review (title)

Lay-led AND systematic review (title)

Mutual aid AND systematic review (title)

Mutual support AND systematic review (title)

Peer adviser AND systematic review (title)

Peer advisers AND systematic review (title)

Peer advisor AND systematic review (title)

Peer advisors AND systematic review (title)

Peer advocate AND systematic review (title)

Peer advocates AND systematic review (title)

Peer based AND systematic review (title)

Peer-based AND systematic review (title)

Peer coach AND systematic review (title)

Peer coaches AND systematic review (title)

Peer coaching AND systematic review (title)

Peer counsellor AND systematic review (title)

Peer counsellors AND systematic review (title)

Peer counselor AND systematic review (title)

Peer counselors AND systematic review (title)

Peer delivered AND systematic review (title)

Peer education AND systematic review (title)

Peer educator AND systematic review (title)

Peer educators AND systematic review (title)

Peer expert AND systematic review (title)

Peer facilitated AND systematic review (title)

Peer facilitator AND systematic review (title)

Peer facilitators AND systematic review (title)

Peer instructor AND systematic review (title)

Peer instructors AND systematic review (title)

Peer leader AND systematic review (title)

Peer leaders AND systematic review (title)

Peer led AND systematic review (title)

Peer-led AND systematic review (title)

Peer mediated AND systematic review (title)

Peer mentor AND systematic review (title)

Peer mentors AND systematic review (title)

Peer mentoring AND systematic review (title)

Peer navigator AND systematic review (title)

Peer navigators AND systematic review (title)

Peer support AND systematic review (title)

Peer supported AND systematic review (title)

Peer supporter AND systematic review (title)

Peer supporters AND systematic review (title)

Peer trainer AND systematic review (title)

Peer trainers AND systematic review (title)

Peer trained AND systematic review (title)

Peer tutor AND systematic review (title)

Peer tutors AND systematic review (title)

Peer worker AND systematic review (title)

Peer workers AND systematic review (title)

Social support intervention AND systematic review (title)

Social support interventions AND systematic review (title)

Volunteer counselor AND systematic review (title)

Volunteer counsellor AND systematic review (title)

Volunteer counselors AND systematic review (title)

Volunteer counsellors AND systematic review (title)

Volunteer led AND systematic review

Volunteer-led AND systematic review

**BL Explore**

<http://explore.bl.uk>

Search completed: 28/01/2021.

Results sorted newest to oldest and screened in BL Explore. 105 records.

“Expert patient” AND systematic review (main title)

“Peer to peer” AND systematic review (main title)

Befriending AND systematic review (main title)

Buddy AND systematic review (main title)

Buddies AND systematic review (main title)

Lay adviser AND systematic review (main title)

Lay advisers AND systematic review (main title)

Lay advisor AND systematic review (main title)

Lay advisors AND systematic review (main title)

Lay advocate AND systematic review (main title)

Lay advocates AND systematic review (main title)

Lay coach AND systematic review (main title)

Lay coaches AND systematic review (main title)

Lay coaching AND systematic review (main title)

Lay facilitator AND systematic review (main title)

Lay facilitators AND systematic review (main title)

Lay facilitated AND systematic review (main title)

Lay health care worker AND systematic review (title)

Lay healthcare worker AND systematic review (title)

Lay health care workers AND systematic review (title)

Lay healthcare workers AND systematic review (title)

Lay instructor AND systematic review (title)

Lay instructors AND systematic review (title)

Lay leader AND systematic review (title)

Lay leaders AND systematic review (title)

Lay mentor AND systematic review (title)

Lay mentors AND systematic review (title)

Lay support AND systematic review (title)

Lay trainer AND systematic review (title)

Lay trainers AND systematic review (title)

Lay trained AND systematic review (title)

Lay led AND systematic review (title)

Lay-led AND systematic review (main title)

Mutual aid AND systematic review (title)

Mutual support AND systematic review (title)

Peer adviser AND systematic review (title)

Peer advisers AND systematic review (title)

Peer advisor AND systematic review (title)

Peer advisors AND systematic review (title)

Peer advocate AND systematic review (title)

Peer advocates AND systematic review (title)

Peer based AND systematic review (title)

Peer-based AND systematic review (title)

Peer coach AND systematic review (title)

Peer coaches AND systematic review (title)

Peer coaching AND systematic review (title)

Peer counsellor AND systematic review (title)

Peer counsellors AND systematic review (title)

Peer counselor AND systematic review (title)

Peer counselors AND systematic review (title)

Peer delivered AND systematic review (title)

Peer education AND systematic review (title)

Peer educator AND systematic review (title)

Peer educators AND systematic review (title)

Peer expert AND systematic review (title)

Peer facilitated AND systematic review (title)

Peer facilitator AND systematic review (title)

Peer facilitators AND systematic review (title)

Peer instructor AND systematic review (title)

Peer instructors AND systematic review (title)

Peer leader AND systematic review (title)

Peer leaders AND systematic review (title)

Peer led AND systematic review (title)

Peer-led AND systematic review (title)

Peer mediated AND systematic review (title)

Peer mentor AND systematic review (title)

Peer mentors AND systematic review (title)

Peer mentoring AND systematic review (title)

Peer navigator AND systematic review (title)

Peer navigators AND systematic review (title)

Peer support AND systematic review (title)

Peer supported AND systematic review (title)

Peer supporter AND systematic review (title)

Peer supporters AND systematic review (title)

Peer trainer AND systematic review (title)

Peer trainers AND systematic review (title)

Peer trained AND systematic review (title)

Peer tutor AND systematic review (title)

Peer tutors AND systematic review (title)

Peer worker AND systematic review (title)

Peer workers AND systematic review (title)

Social support intervention AND systematic review (title)

Social support interventions AND systematic review (title)

Volunteer counselor AND systematic review (title)

Volunteer counsellor AND systematic review (title)

Volunteer counselors AND systematic review (title)

Volunteer counsellors AND systematic review (title)

Volunteer led AND systematic review (title)

Volunteer-led AND systematic review (title)

**PROSPERO**

<https://www.crd.york.ac.uk/prospero/>

Search completed: 16/08/2021

#1 MeSH DESCRIPTOR Peer Group EXPLODE ALL TREES 80

#2 "mutual support" 15

#3 "consumer case management" 0

#4 "consumer-delivered" 2

#5 "consumer-provided" 0

#6 "consumer provided" 0

#7 "lay leader" 2

#8 "lay leaders" 3

#9 "lay-led" 12

#10 "peer administered" 0

#11 "peer adviser" 0

#12 "peer advisers" 0

#13 "peer advisor" 0

#14 "peer advisors" 1

#15 "peer advocate" 0

#16 "peer advocates" 0

#17 "peer-based" 27

#18 "peer coach" 3

#19 "peer coaches" 5

#20 "peer coaching" 6

#21 "peer co-facilitated" 0

#22 "peer co-led" 1

#23 "peer counselor" 2

#24 "peer counselors" 3

#25 "peer counsellor" 1

#26 "peer counsellors" 4

#27 "peer counseling" 10

#28 "peer counselling" 8

#29 "peer delivered" 17

#30 "peer facilitated" 6

#31 "peer facilitator" 6

#32 "peer facilitators" 4

#33 "peer helper" 0

#34 "peer helpers" 3

#35 "peer implemented" 0

#36 "peer intervention" 14

#37 "peer interventions" 9

#38 "peer leader" 9

#39 "peer leader" 9

#40 "peer leader" 9

#41 "peer leader" 9

#42 "peer leader" 9

#43 "peer leader" 9

#44 "peer leaders" 7

#45 "peer led" 69

#46 "peer mentor" 12

#47 "peer mentors" 15

#48 "peer mentoring" 23

#49 "peer mentorship" 2

#50 "peer navigator" 3

#51 "peer navigators" 3

#52 "peer navigation" 2

#53 "peer provider" 1

#54 "peer providers" 6

#55 "peer run" 5

#56 "peer specialist" 1

#57 "peer specialists" 0

#58 "peer support" 390

#59 "peer supporter" 6

#60 "peer supporters" 18

#61 "peer supported" 5

#62 "peer trainer" 0

#63 "peer trainers" 0

#64 "peer trained" 0

#65 "peer program" 0

#66 "peer programs" 0

#67 "peer worker" 6

#68 "peer workers" 6

#69 #1 OR #2 OR #3 OR #4 OR #5 OR #6 OR #7 OR #8 OR #9 OR #10 OR #11 OR #12 OR #13 OR #14 OR #15 OR #16 OR #17 OR #18 OR #19 OR #20 OR #21 OR #22 OR #23 OR #24 OR #25 OR #26 OR #27 OR #28 OR #29 OR #30 OR #31 OR #32 OR #33 OR #34 OR #35 OR #36 OR #37 OR #38 OR #39 OR #40 OR #41 OR #42 OR #43 OR #44 OR #45 OR #46 OR #47 OR #48 OR #49 OR #50 OR #51 OR #52 OR #53 OR #54 OR #55 OR #56 OR #57 OR #58 OR #59 OR #60 OR #61 OR #62 OR #63 OR #64 OR #65 OR #66 OR #67 OR #68 (588)

**Stage 2 search strategies
Searches for RCTs**

**Ovid MEDLINE (ALL 1946 to September 29, 2020)**Search completed: 15/03/2021

1 randomized controlled trial.pt. (524960)

2 controlled clinical trial.pt. (94095)

3 randomi?ed.ab. (613218)

4 placebo.ab. (216056)

5 exp Clinical Trials as Topic/ (353812)

6 randomly.ab. (353046)

7 trial.ti. (236085)

8 1 or 2 or 3 or 4 or 5 or 6 or 7 (1459955)

9 exp animals/ not humans.sh. (4799281)

10 8 not 9 (1351468)

11 *Peer Group/ (9557)

12 (Peer* adj support*).ab. (4633)

13 (Peer* adj3 support*).ti. (1421)

14 (peer* adj1 led).tw. (1159)

15 (peer* adj2 mentor*).tw. (924)

16 (peer* adj2 program*).tw. (1638)

17 (peer* adj3 group*).ti. (650)

18 (peer* adj group*).ab. (2660)

19 (peer* adj2 coach*).tw. (325)

20 (peer* adj2 counsel*).tw. (731)

21 (peer* adj2 deliver*).tw. (433)

22 (peer* adj2 educat*).tw. (2253)

23 (peer* adj2 expert*).ti. (30)

24 (peer* adj1 expert*).ab. (122)

25 (peer* adj2 facilitat*).tw. (547)

26 (peer* adj2 leader*).tw. (487)

27 (peer* adj2 navigat*).tw. (133)

28 (peer* adj2 provider*).tw. (233)

29 (peer* adj specialist*).tw. (122)

30 (peer* adj2 trained).tw. (454)

31 (peer* adj2 cofacilitat*).tw. (3)

32 (peer* adj2 co facilitat*).tw. (9)

33 "peer to peer".ti. (293)

34 (peer* adj1 led).tw. (1159)

35 (peer* adj3 run*).ti. (26)

36 (peer* adj1 run*).ab. (53)

37 (lay adj2 led).tw. (92)

38 "mutual support".tw. (671)

39 (expert adj patient*).tw. (270)

40 (peer* adj2 administer*).tw. (53)

41 (peer* adj2 adviser*).tw. (10)

42 (peer* adj2 advisor*).tw. (46)

43 (peer* adj2 advocate*).tw. (74)

44 (peer* adj2 consultant*).tw. (39)

45 (peer* adj2 helper*).tw. (37)

46 (peer* adj2 implement*).tw. (329)

47 (peer* adj2 instructor*).tw. (94)

48 (peer* adj2 intervention*).tw. (1370)

49 (peer* adj2 listener*).tw. (6)

50 (peer* adj2 mediated).tw. (271)

51 (peer* adj2 mentor*).tw. (924)

52 (peer* adj2 network*).tw. (914)

53 (peer* adj2 provider*).tw. (233)

54 (peer* adj specialist*).tw. (122)

55 (peer* adj2 trainer*).tw. (68)

56 (peer* adj2 trained).tw. (454)

57 (peer* adj2 tutor*).tw. (310)

58 (peer* adj2 volunteer*).tw. (216)

59 (peer* adj2 worker*).tw. (438)

60 "peer based".tw. (445)

61 (lay adj2 leader*).tw. (131)

62 "mutual aid".tw. (393)

63 "mutual help".ti,ab. (312)

64 "shared experience".tw. (387)

65 (survivor* adj2 deliver*).tw. (129)

66 (survivor* adj led).tw. (25)

67 (consumer* adj provide*).tw. (245)

68 (consumer* adj deliver*).tw. (10)

69 "consumer case management".tw. (8)

70 or/11-69 (24942)

71 10 and 70 (3190)

**EBSCOhost CINAHL Complete**

Search completed: 15/03/2021

S72 S55 AND S71 2,026

S71 S70 NOT S69 437,678

S70 S56 OR S57 OR S58 OR S59 OR S60 OR S61 OR S62 OR S63

S69 S67 NOT S68

S68 (MH "Human")

S67 S64 OR S65 OR S66

S66 TI animal model*

S65 (MH "Animal Studies")

S64 (MH "Animals+")

S63 PT randomized controlled trial

S62 TI trial

S61 AB random*

S60 TI randomised or randomized

S59 (MH "Random Assignment")

S58 (MH "Single-Blind Studies")

S57 (MH "Double-Blind Studies")

S56 (MH "Randomized Controlled Trials")

S55 S1 OR S2 OR S3 OR S4 OR S5 OR S6 OR S7 OR S8 OR S9 OR S10 OR S11 OR S12 OR S13 OR S14 OR S15 OR S16 OR S17 OR S18 OR S19 OR S20 OR S21 OR S22 OR S23 OR S24 OR S25 OR S26 OR S27 OR S28 OR S29 OR S30 OR S31 OR S32 OR S33 OR S34 OR S35 OR S36 OR S37 OR S38 OR S39 OR S40 OR S41 OR S42 OR S43 OR S44 OR S45 OR S46 OR S47 OR S48 OR S49 OR S50 OR S51 OR S52 OR S53 OR S54 17,641

S54 TI "consumer case management" OR AB "consumer case management"

S53 TI consumer* W0 deliver* OR AB consumer* W0 deliver*

S52 TI consumer* W0 provide* OR AB consumer* W0 provide*

S51 TI survivor* W0 led OR AB survivor* W0 led

S50 TI survivor* N1 deliver* OR AB survivor* N1 deliver*

S49 TI "shared experience" OR AB "shared experience"

S48 TI "mutual help" OR AB "mutual help"

S47 TI "mutual aid" OR AB "mutual aid"

S46 TI lay N1 leader* OR AB lay N1 leader*

S45 TI "peer based" OR AB "peer based"

S44 TI (peer* N1 (worker* or volunteer)) OR AB peer* N1 (worker* or volunteer))

S43 TI peer* N1 tutor* OR AB peer* N1 tutor*

S42 TI peer* N1 trainer* OR AB peer* N1 trainer*

S41 TI peer* N1 network* OR AB peer* N1 network*

S40 TI peer* N1 mentor* OR AB peer* N1 mentor*

S39 TI peer* N1 mediated OR AB peer* N1 mediated

S38 TI peer* N1 listener* OR AB peer* N1 listener*

S37 TI peer* N2 intervention* OR AB peer* N1 intervention*

S36 TI peer* N1 instructor* OR AB peer* N1 instructor*

S35 TI peer* N1 implement* OR AB peer* N1 implement*

S34 TI peer* N1 helper* OR AB peer* N1 helper*

S33 TI peer* N1 consultant* OR AB peer* N1 consultant*

S32 TI peer* N1 advocate* OR AB peer* N1 advocate*

S31 TI peer* N1 advisor* OR AB peer* N1 advisor*

S30 TI peer* N1 adviser* OR AB peer* N1 adviser*

S29 TI peer* N1 administer* OR AB peer* N1 administer*

S28 AB expert W0 patient* OR TI expert W0 patient*

S27 AB "mutual support" OR TI "mutual support"

S26 AB lay N1 led OR TI lay N1 led

S25 AB peer* N0 run*

S24 TI peer* N2 run*

S23 TI "peer to peer"

S22 AB (peer* N1 co facilitat*) OR TI (peer* N1 co facilitat*)

S21 AB (peer* N1 cofacilitat*) OR TI (peer* N1 cofacilitat*)

S20 AB (peer* N1 trained) OR TI (peer* N1 trained)

S19 AB (peer* W0 specialist*) OR TI (peer* W0 specialist*)

S18 AB (peer* N1 provider*) OR TI (peer* N1 provider*)

S17 AB (peer* N1 navigat*) OR TI (peer* N1 navigat*)

S16 AB (peer* N1 leader*) OR TI (peer* N1 leader*)

S15 AB (peer* N1 facilitat*) OR TI (peer* N1 facilitat*)

S14 AB (peer* N0 expert*)

S13 TI (peer* N1 expert*)

S12 TI (peer* N2 educat*) OR AB (peer* N1 educat*)

S11 TI (peer* N1 deliver*) OR AB (peer* N1 deliver*)

S10 TI (peer* N1 counsel*) OR AB (peer* N1 counsel*)

S9 TI (peer* N1 coach*) OR AB (peer* N1 coach*)

S8 AB (peer* W0 group*)

S7 TI (peer* N2 group*)

S6 TI (peer* N1 program*) OR AB (peer* N1 program*)

S5 TI (peer* N1 mentor*) OR AB (peer* N1 mentor*)

S4 TI (peer* N0 led) OR AB (peer* N0 led)

S3 TI peer* N3 support*

S2 AB peer* W0 support*

S1 (MM "Peer Group")

**Ovid APA PsycINFO (1806 to March Week 2 2021)**

Search completed: 16/03/2021

1 exp randomized controlled trials/ (884)

2 randomi?ed.ti,ab. (88601)

3 random*.ab. (206679)

4 trial.ti. (32316)

5 or/1-4 (217377)

6 exp animals/ not humans.sh. (356344)

7 5 not 6 (208120)

8 peer counseling/ (1149)

9 *peers/ (7661)

10 *peer relations/ (12463)

11 (Peer* adj support*).ab. (4447)

12 (Peer* adj3 support*).ti. (1249)

13 (peer* adj1 led).tw. (884)

14 (peer* adj2 mentor*).tw. (1168)

15 (peer* adj2 program*).tw. (1790)

16 (peer* adj3 group*).ti. (1358)

17 (peer* adj group*).ab. (5596)

18 (peer* adj2 coach*).tw. (511)

19 (peer* adj2 counsel*).tw. (1032)

20 (peer* adj2 deliver*).tw. (344)

21 (peer* adj2 educat*).tw. (1745)

22 (peer* adj2 expert*).ti. (33)

23 (peer* adj1 expert*).ab. (75)

24 (peer* adj2 facilitat*).tw. (607)

25 (peer* adj2 leader*).tw. (670)

26 (peer* adj2 navigat*).tw. (89)

27 (peer* adj2 provider*).tw. (197)

28 (peer* adj specialist*).tw. (138)

29 (peer* adj2 trained).tw. (327)

30 (peer* adj2 cofacilitat*).tw. (4)

31 (peer* adj2 co facilitat*).tw. (6)

32 "peer to peer".ti. (275)

33 (peer* adj1 led).tw. (884)

34 (peer* adj3 run*).ti. (25)

35 (peer* adj1 run*).ab. (87)

36 (lay adj2 led).tw. (50)

37 "mutual support".tw. (887)

38 (expert adj patient*).tw. (105)

39 (peer* adj2 administer*).tw. (75)

40 (peer* adj2 adviser*).tw. (18)

41 (peer* adj2 advisor*).tw. (62)

42 (peer* adj2 advocate*).tw. (86)

43 (peer* adj2 consultant*).tw. (32)

44 (peer* adj2 helper*).tw. (130)

45 (peer* adj2 implement*).tw. (361)

46 (peer* adj2 instructor*).tw. (244)

47 (peer* adj2 intervention*).tw. (1455)

48 (peer* adj2 listener*).tw. (20)

49 (peer* adj2 mediated).tw. (764)

50 (peer* adj2 mentor*).tw. (1168)

51 (peer* adj2 network*).tw. (1271)

52 (peer* adj2 provider*).tw. (197)

53 (peer* adj specialist*).tw. (138)

54 (peer* adj2 trainer*).tw. (66)

55 (peer* adj2 trained).tw. (327)

56 (peer* adj2 tutor*).tw. (1221)

57 (peer* adj2 volunteer*).tw. (143)

58 (peer* adj2 worker*).tw. (360)

59 "peer based".tw. (490)

60 (lay adj2 leader*).tw. (208)

61 "mutual aid".tw. (736)

62 "mutual help".ti,ab. (456)

63 "shared experience".tw. (757)

64 (survivor* adj2 deliver*).tw. (42)

65 (survivor* adj led).tw. (27)

66 (consumer* adj provide*).tw. (250)

67 (consumer* adj deliver*).tw. (12)

68 "consumer case management".tw. (7)

69 or/8-68 (39376)

70 7 and 69 (2214)

**Cochrane Central Register of Controlled Trials (CENTRAL)**Search completed: 17/03/2021

#1 ((Support* or Led or Mentor* or Program* or Group* or Coach* or Counsel* or Deliver* or Educat* or Expert* or Facilitat* or leader* or Navigat* or Provider* or Specialist* or Trained or Cofacilitat* or run* or administer* or adviser* or advisor* or advocate* or consultant* or helper or implement* or instructor* or intervention* or listener* or mediated or mentor* or network* or provider* or specialist* or trainer* or trained or tutor* or volunteer* or worker*) NEAR/1 (peer or peers)):ti,ab 3724

#2 "peer based":ti,ab 93

#3 lay NEAR/1 leader*:ti,ab 27

#4 "mutual support*":ti,ab 91

#5 expert NEAR/2 patient*:ti,ab 162

#6 mutual NEAR/2 (aid or help):ti,ab 57

#7 "shared experience":ti,ab 29

#8 survivor* NEAR/2 deliver*:ti,ab 21

#9 survivor* NEAR/2 led:ti,ab 19

#10 consumer* NEAR/2 provide*:ti,ab 85

#11 consumer* NEAR/2 deliver*:ti,ab 15

#12 "consumer case management":ti,ab 8

#13 #1 or #2 or #3 or #4 or #5 or #6 or #7 or #8 or #9 or #10 or #11 or #12 4239

#14 MeSH descriptor: [Peer Group] explode all trees 1430

#15 #13 or #14 4926

**Google Scholar**

Search completed: 9/08/2021 via Publish or Perish (Harzing).No date limit applied. 732 records exported from PoP.

“Mutual support” AND randomized OR randomised OR RCT

“consumer case management” AND randomized OR randomised OR RCT

“consumer delivered” AND randomized OR randomised OR RCT

“consumer provided” AND randomized OR randomised OR RCT

“lay-leader” AND randomized OR randomised OR RCT

“lay-leaders” AND randomized OR randomised OR RCT

“lay-led” AND randomized OR randomised OR RCT

“peer administered” AND randomized OR randomised OR RCT

“Peer adviser” AND randomized OR randomised OR RCT

“Peer advisers” AND randomized OR randomised OR RCT

“Peer advisor” AND randomized OR randomised OR RCT

“Peer advisors” AND randomized OR randomised OR RCT

“Peer advocate” AND randomized OR randomised OR RCT

“Peer advocates” AND randomized OR randomised OR RCT

“Peer-based” AND randomized OR randomised OR RCT

“Peer based” AND randomized OR randomised OR RCT – same as above

“Peer coach” AND randomized OR randomised OR RCT

“Peer coaches” AND randomized OR randomised OR RCT

“Peer coaching” AND randomized OR randomised OR RCT

“peer co-facilitated” AND randomized OR randomised OR RCT

“peer co-led” AND randomized OR randomised OR RCT

“Peer counselor” AND randomized OR randomised OR RCT

“Peer counselors” AND randomized OR randomised OR RCT

“Peer counsellor” AND randomized OR randomised OR RCT

“Peer counsellors” AND randomized OR randomised OR RCT

“Peer counselling” AND randomized OR randomised OR RCT

“Peer counselling” AND randomized OR randomised OR RCT

“Peer delivered” AND randomized OR randomised OR RCT

“Peer facilitated” AND randomized OR randomised OR RCT

“Peer facilitator” AND randomized OR randomised OR RCT

“Peer facilitators” AND randomized OR randomised OR RCT

“peer helper” AND randomized OR randomised OR RCT

“peer helper” AND randomized OR randomised OR RCT

“Peer implemented” AND randomized OR randomised OR RCT

“peer intervention” AND randomized OR randomised OR RCT

“Peer leader” AND randomized OR randomised OR RCT

“Peer leaders” AND randomized OR randomised OR RCT

“Peer led” AND randomized OR randomised OR RCT

“Peer mentor” AND randomized OR randomised OR RCT

“Peer mentors” AND randomized OR randomised OR RCT

“Peer mentoring” AND randomized OR randomised OR RCT

“Peer mentorship” AND randomized OR randomised OR RCT

“Peer navigator” AND randomized OR randomised OR RCT

“Peer navigators” AND randomized OR randomised OR RCT

“Peer navigation” AND randomized OR randomised OR RCT

“peer provider” AND randomized OR randomised OR RCT

“peer provider” AND randomized OR randomised OR RCT

“Peer run” AND randomized OR randomised OR RCT

“peer specialist” AND randomized OR randomised OR RCT

“peer specialists” AND randomized OR randomised OR RCT

“peer support” AND randomized OR randomised OR RCT

“peer support” AND randomized OR randomised OR RCT

“peer supporter” AND randomized OR randomised OR RCT

“peer supporters” AND randomized OR randomised OR RCT

“peer trainer” AND randomized OR randomised OR RCT

“peer trainers” AND randomized OR randomised OR RCT

“peer trained” AND randomized OR randomised OR RCT

“peer program” AND randomized OR randomised OR RCT

“peer programs” AND randomized OR randomised OR RCT

“peer worker” AND randomized OR randomised OR RCT

“peer workers” AND randomized OR randomised OR RCT

**Searches for ongoing trials**

**Cochrane Central Register of Controlled Trials (CENTRAL)**Search completed: 21/05/2021. No date limits applied

ID Search Hits
#1 peer:ti
#2 Peer NEAR/2 (support* or group*):ab
#3 Lay:ti
#4 (support* NEAR/2 group*):ti
#5 #1 or #2 or #3 or #4

**International Clinical Trials Registry Platform (ICTRP)**Search completed: 21/05/2021. No date limits applied

Using the advanced search:
Peer in title with synonyms and status: ALL
OR
Peer support in intervention

**ClinicalTrials.gov**
Search completed: 21/05/2021. No date limits applied

Peer in title
OR
Peer support in intervention
(Limited to adults or older adults and interventional studies)

**Searches for economic studies**

**Ovid MEDLINE (ALL 1946 to May 12, 2021)**Search completed: 31/05/2021

1 *Peer Group/ 9641

2 (Peer* adj support*).ab. 4754

3 (Peer* adj3 support*).ti. 1449

4 (peer* adj1 led).tw. 1186

5 (peer* adj2 mentor*).tw. 947

6 (peer* adj2 program*).tw. 1668

7 (peer* adj3 group*).ti. 659

8 (peer* adj group*).ab. 2687

9 (peer* adj2 coach*).tw. 336

10 (peer* adj2 counsel*).tw. 737

11 (peer* adj2 deliver*).tw. 445

12 (peer* adj2 educat*).tw. 2283

13 (peer* adj2 expert*).ti. 31

14 (peer* adj1 expert*).ab. 123

15 (peer* adj2 facilitat*).tw. 559

16 (peer* adj2 leader*).tw. 498

17 (peer* adj2 navigat*).tw. 135

18 (peer* adj2 provider*).tw. 240

19 (peer* adj specialist*).tw. 124

20 (peer* adj2 trained).tw. 462

21 (peer* adj2 cofacilitat*).tw. 3

22 (peer* adj2 co facilitat*).tw. 9

23 "peer to peer".ti. 299

24 (peer* adj1 led).tw. 1186

25 (peer* adj3 run*).ti. 26

26 (peer* adj1 run*).ab. 53

27 (lay adj2 led).tw. 95

28 "mutual support".tw. 686

29 (expert adj patient*).tw. 274

30 (peer* adj2 administer*).tw. 54

31 (peer* adj2 adviser*).tw. 10

32 (peer* adj2 advisor*).tw. 46

33 (peer* adj2 advocate*).tw. 75

34 (peer* adj2 consultant*).tw. 40

35 (peer* adj2 helper*).tw. 37

36 (peer* adj2 implement*).tw. 339

37 (peer* adj2 instructor*).tw. 95

38 (peer* adj2 intervention*).tw. 1404

39 (peer* adj2 listener*).tw. 6

40 (peer* adj2 mediated).tw. 273

41 (peer* adj2 mentor*).tw. 947

42 (peer* adj2 network*).tw. 931

43 (peer* adj2 provider*).tw. 240

44 (peer* adj specialist*).tw. 124

45 (peer* adj2 trainer*).tw. 69

46 (peer* adj2 trained).tw. 462

47 (peer* adj2 tutor*).tw. 314

48 (peer* adj2 volunteer*).tw. 218

49 (peer* adj2 worker*).tw. 445

50 "peer based".tw. 453

51 (lay adj2 leader*).tw. 133

52 "mutual aid".tw. 397

53 "mutual help".ti,ab. 319

54 "shared experience".tw. 397

55 (survivor* adj2 deliver*).tw. 133

56 (survivor* adj led).tw. 26

57 (consumer* adj provide*).tw. 246

58 (consumer* adj deliver*).tw. 10

59 "consumer case management".tw. 8

60 or/1-59 25347

61 *economics/ 10739

62 exp *"costs and cost analysis"/ 74182

63 cost-benefit analysis/ 84342

64 (economic adj2 model*).mp. 13712

65 (cost minimi* or cost-utilit* or health utilit* or economic evaluation* or economic review* or cost outcome or cost analys?s or economic analys?s or budget* impact analys?s).ti,ab,kf,kw. 35068

66 (cost-effective* or pharmacoeconomic* or pharmaco-economic* or cost-benefit or costs).ti,kf,kw. 76616

67 (life year or life years or qaly* or cost-benefit analys?s or cost-effectiveness analys?s).ab,kf,kw. 32546

68 (cost or economic*).ti,kf,kw. and (costs or cost-effectiveness or markov).ab. 61298

69 or/61-68 223511

70 60 and 69 372

**Ovid Embase (1974 to 2021 May 12)**

Search completed: 13/05/2021

1 *Peer Group/ 8267

2 peer counselling/ 626

3 (Peer* adj support*).ab. 6878

4 (Peer* adj3 support*).ti. 1922

5 (peer* adj1 led).tw. 1604

6 (peer* adj2 mentor*).tw. 1372

7 (peer* adj2 program*).tw. 2284

8 (peer* adj3 group*).ti. 793

9 (peer* adj group*).ab. 3661

10 (peer* adj2 coach*).tw. 477

11 (peer* adj2 counsel*).tw. 953

12 (peer* adj2 deliver*).tw. 573

13 (peer* adj2 educat*).tw. 3075

14 (peer* adj2 expert*).ti. 37

15 (peer* adj1 expert*).ab. 171

16 (peer* adj2 facilitat*).tw. 802

17 (peer* adj2 leader*).tw. 670

18 (peer* adj2 navigat*).tw. 234

19 (peer* adj2 provider*).tw. 293

20 (peer* adj specialist*).tw. 150

21 (peer* adj2 trained).tw. 622

22 (peer* adj2 cofacilitat*).tw. 5

23 (peer* adj2 co facilitat*).tw. 12

24 "peer to peer".ti. 410

25 (peer* adj1 led).tw. 1604

26 (peer* adj3 run*).ti. 31

27 (peer* adj1 run*).ab. 72

28 (lay adj2 led).tw. 126

29 "mutual support".tw. 893

30 (expert adj patient*).tw. 494

31 (peer* adj2 administer*).tw. 75

32 (peer* adj2 adviser*).tw. 12

33 (peer* adj2 advisor*).tw. 68

34 (peer* adj2 advocate*).tw. 92

35 (peer* adj2 consultant*).tw. 49

36 (peer* adj2 helper*).tw. 45

37 (peer* adj2 implement*).tw. 425

38 (peer* adj2 instructor*).tw. 112

39 (peer* adj2 intervention*).tw. 1729

40 (peer* adj2 listener*).tw. 4

41 (peer* adj2 mediated).tw. 286

42 (peer* adj2 mentor*).tw. 1372

43 (peer* adj2 network*).tw. 1182

44 (peer* adj2 provider*).tw. 293

45 (peer* adj specialist*).tw. 150

46 (peer* adj2 trainer*).tw. 89

47 (peer* adj2 trained).tw. 622

48 (peer* adj2 tutor*).tw. 394

49 (peer* adj2 volunteer*).tw. 329

50 (peer* adj2 worker*).tw. 573

51 "peer based".tw. 570

52 (lay adj2 leader*).tw. 156

53 "mutual aid".tw. 484

54 "mutual help".ti,ab. 424

55 "shared experience".tw. 531

56 (survivor* adj2 deliver*).tw. 229

57 (survivor* adj led).tw. 43

58 (consumer* adj provide*).tw. 283

59 (consumer* adj deliver*).tw. 14

60 "consumer case management".tw. 8

61 or/1-60 30755

62 *economics/ 27019

63 exp *economic evaluation/ 66494

64 cost-benefit analysis/ 88277

65 (economic adj2 model*).mp. 8254

66 (cost minimi* or cost-utilit* or health utilit* or economic evaluation* or economic review* or cost outcome or cost analys?s or economic analys?s or budget* impact analys?s).ti,ab,kw. 55184

67 (cost-effective* or pharmacoeconomic* or pharmaco-economic* or cost-benefit or costs).ti,kw. 115188

68 (life year or life years or qaly* or cost-benefit analys?s or cost-effectiveness analys?s).ab,kw. 51351

69 (cost or economic*).ti,kw. and (costs or cost-effectiveness or markov).ab. 98298

70 or/62-69 286964

71 61 and 70 402

**HTA database and NHS Economic Evaluation Database (NHS EED) via the Centre for Reviews and Dissemination**

<https://www.crd.york.ac.uk/CRDWeb/AboutPage.asp>

Search completed 13/05/2021. Bibliographic records were published on HTA and NHS EED until 31^st^ March 2015.

1 MeSH DESCRIPTOR Peer Group EXPLODE ALL TREES 83

2 (peer* NEAR1 support*) 83

3 (peer* NEAR3 support*):TI 36

4 (peer* NEAR1 led) 27

5 (peer* NEAR2 mentor*) 6

6 (peer* NEAR2 program*) 27

7 (peer* NEAR3 group*):TI 2

8 (peer* NEAR1 group*) 103

9 (peer* NEAR2 coach*) 2

10 (peer* NEAR2 counsel*) 32

11 (peer* NEAR2 deliver*) 15

12 (peer* NEAR2 educat*) 37

13 (peer* NEAR2 expert*):TI 0

14 (peer* NEAR1 expert*) 1

15 (peer* NEAR2 facilitat*) 3

16 (peer* NEAR2 leader*) 13

17 (peer* NEAR2 navigat*) 0

18 (peer* NEAR2 provider*) 1

19 (peer* NEAR1 specialist*) 1

20 (peer* NEAR2 trained) 0

21 (peer* NEAR2 cofacilitat*) 0

22 (peer* NEAR2 co facilitat*) 1

23 (peer to peer):TI 1

24 (peer* NEAR3 run*):TI 0

25 (peer* NEAR1 run*) 0

26 (lay NEAR2 led) 5

27 (mutual support) 9

28 (expert patient*) 8

29 (peer NEAR2 administer*) 0

30 (peer NEAR2 advisor*) 3

31 (peer NEAR2 adviser*) 1

32 (peer NEAR2 advocate*) 2

33 (peer NEAR2 consultant*) 0

34 (peer NEAR2 helper*) 0

35 (peer NEAR2 implement*) 0

36 (peer NEAR2 instructor*) 0

37 (peer NEAR2 intervention*) 61

38 (peer NEAR2 listener*) 0

39 (peer NEAR2 mediated) 3

40 (peer NEAR2 mentor*) 6

41 (peer NEAR2 network*) 1

42 (peer NEAR2 provider*) 1

43 (peer NEAR1 specialist*) 1

44 (peer NEAR2 trainer*) 1

45 (peer NEAR2 trained) 0

46 (peer NEAR2 tutor*) 1

47 (peer NEAR2 volunteer*) 2

48 (peer NEAR2 worker*) 5

49 (peer-based) 7

50 (peer based) 7

51 (mutual aid) 3

52 (mutual help) 3

53 (shared experience) 0

54 (survivor* NEAR2 deliver*) 0

55 (survivor* NEAR1 led) 0

56 (consumer* NEAR1 provide*) 8

57 (consumer* NEAR1 deliver*) 6

58 (consumer case management) 0

59 #1 OR #2 OR #3 OR #4 OR #5 OR #6 OR #7 OR #8 OR #9 OR #10 OR #11 OR #12 OR #13 OR #14 OR #15 OR #16 OR #17 OR #18 OR #19 OR #20 OR #21 OR #22 OR #23 OR #24 OR #25 OR #26 OR #27 OR #28 OR #29 OR #30 OR #31 OR #32 OR #33 OR #34 OR #35 OR #36 OR #37 OR #38 OR #39 OR #40 OR #41 OR #42 OR #43 OR #44 OR #45 OR #46 OR #47 OR #48 OR #49 OR #50 OR #51 OR #52 OR #53 OR #54 OR #55 OR #56 OR #57 OR #58 274

60 (* ) and ((Economic evaluation:ZDT and Bibliographic:ZPS) OR (Economic evaluation:ZDT and Abstract:ZPS)) IN NHSEED 17612

61 #59 AND #60 50

62 (* ) and (Project record:ZDT OR Full publication record:ZDT) IN HTA 17320

63 #59 AND #62

**INAHTA international HTA database**

<https://database.inahta.org/>

Search completed: 27/05/2021. 20 records exported.

("lay leaders") OR ("lay leader") OR ("lay led") OR (((lay AND led))[title]) OR ("consumer case management") OR ("consumer providers") OR ("consumer provider") OR ("consumer provided") OR (((consumer* AND provide*))[title]) OR ("consumer delivered") OR ((consumer* AND deliver*)[title]) OR ("shared experience") OR ("mutual help") OR ("mutual aid") OR ("peer-based") OR ("peer based") OR ("peer interventions") OR ("peer intervention") OR ((peer* and intervention*)[title]) OR ("peer-implemented") OR ("peer implemented") OR (((peer* AND implement*))[title]) OR ("peer helpers") OR ("peer helper") OR (((peer* AND helper*))[title]) OR ("peer consultants") OR ("peer consultant") OR ((peer* and consultant*)[title]) OR ("peer advocates") OR ("peer advocate") OR (((peer* AND advocate*))[title]) OR ("peer advisers") OR ("peer adviser") OR (((peer* AND adviser*))[title]) OR ("peer advisors") OR ("peer advisor") OR (((peer* AND advisor*))[title]) OR ("peer administered") OR (((peer* AND administer*))[title]) OR ("peer run") OR ("peer to peer") OR ("peer co-facilitated") OR ("peer cofacilitated") OR ("peer cofacilitators") OR ("peer cofacilitator") OR (((peer* and co-facilitat*))[title]) OR (((peer* and cofacilitat*))[title]) OR ("peer training") OR ("peer trained") OR ("peer trainers") OR ("peer trainer") OR (((peer* and train*))[title]) OR ("peer providers") OR ("peer provider") OR ("peer provided") OR ((peer* AND provide*)[title]) OR (("peer navigation")[title]) OR (("peer navigators")[title]) OR (("peer navigator")[title]) OR (((peer* AND navigat*))[title]) OR ("peer leaders") OR ("peer leader") OR (((peer* AND leader*))[title]) OR ("peer facilitators") OR ("peer facilitator") OR ("peer facilitated") OR ((peer* AND facilitat*)[title]) OR ("peer educators") OR ("peer educator") OR ("peer educated") OR ("peer education") OR (((peer* AND educat*)[title]) OR ("peer delivery") OR ("peer delivered") OR ((peer* AND deliver*)[title]) OR ("Peer counselors") OR ("Peer counsellors") OR ("Peer counselor") OR ("Peer counsellor") OR ("Peer counseling") OR ("Peer counselling") OR (((peer* AND counsel*))[title]) OR ("peer coaching") OR ("peer coached") OR ("peer coaches") OR ("peer coach") OR (((peer* AND coach*))[title]) OR ("peer mentoring") OR ("peer mentored") OR ("peer mentors") OR ("peer mentor") OR (((peer* AND mentor*))[title]) OR ("peer led") OR ((peer* AND led)[title]) OR ("peer support") OR ((peer* AND support*)[title]) OR ("peer group") OR ("Peer Group"[mh])

**Search Summary Table Stage 1**

**
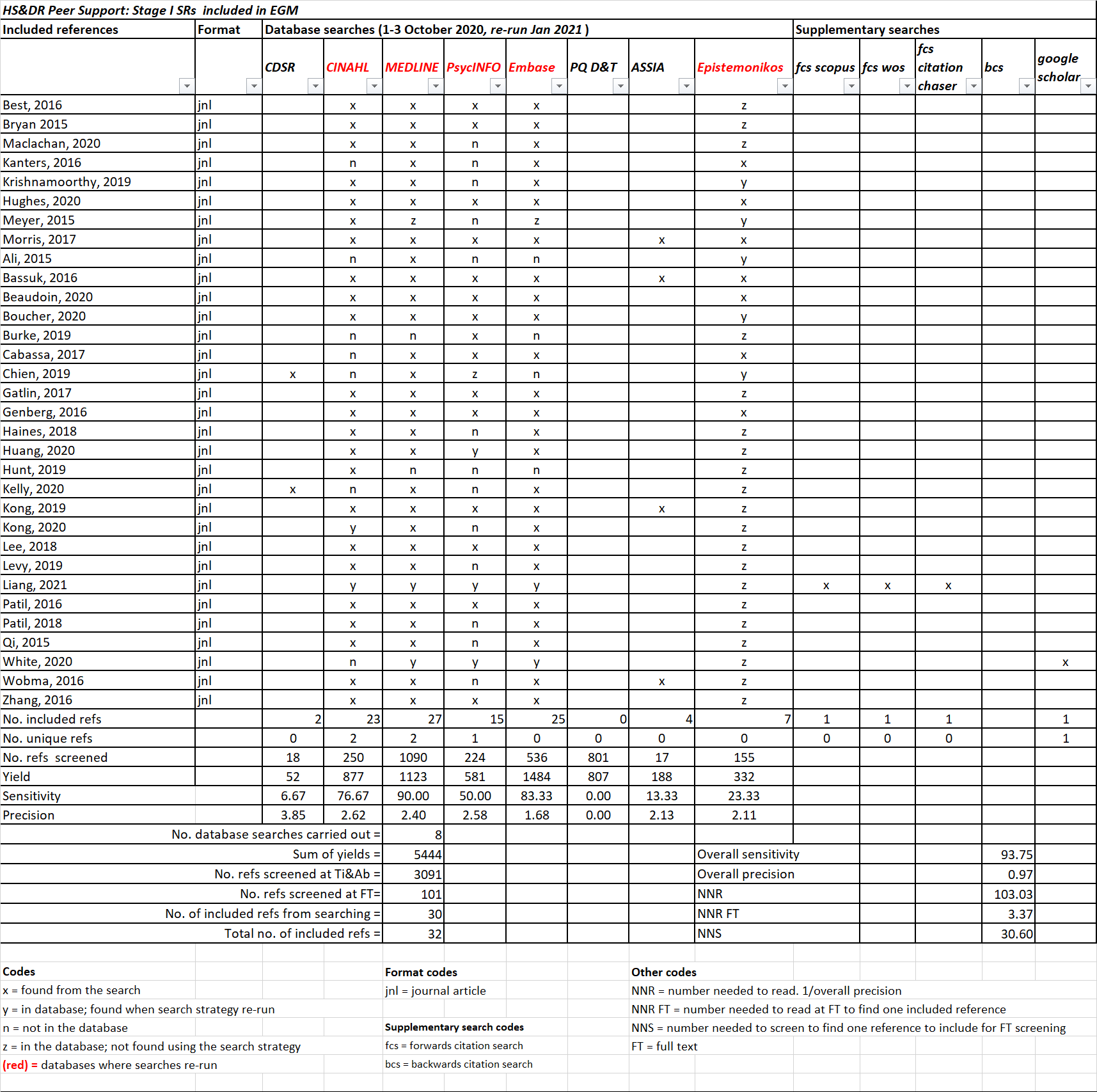
**

**Search Summary Table Stage 2
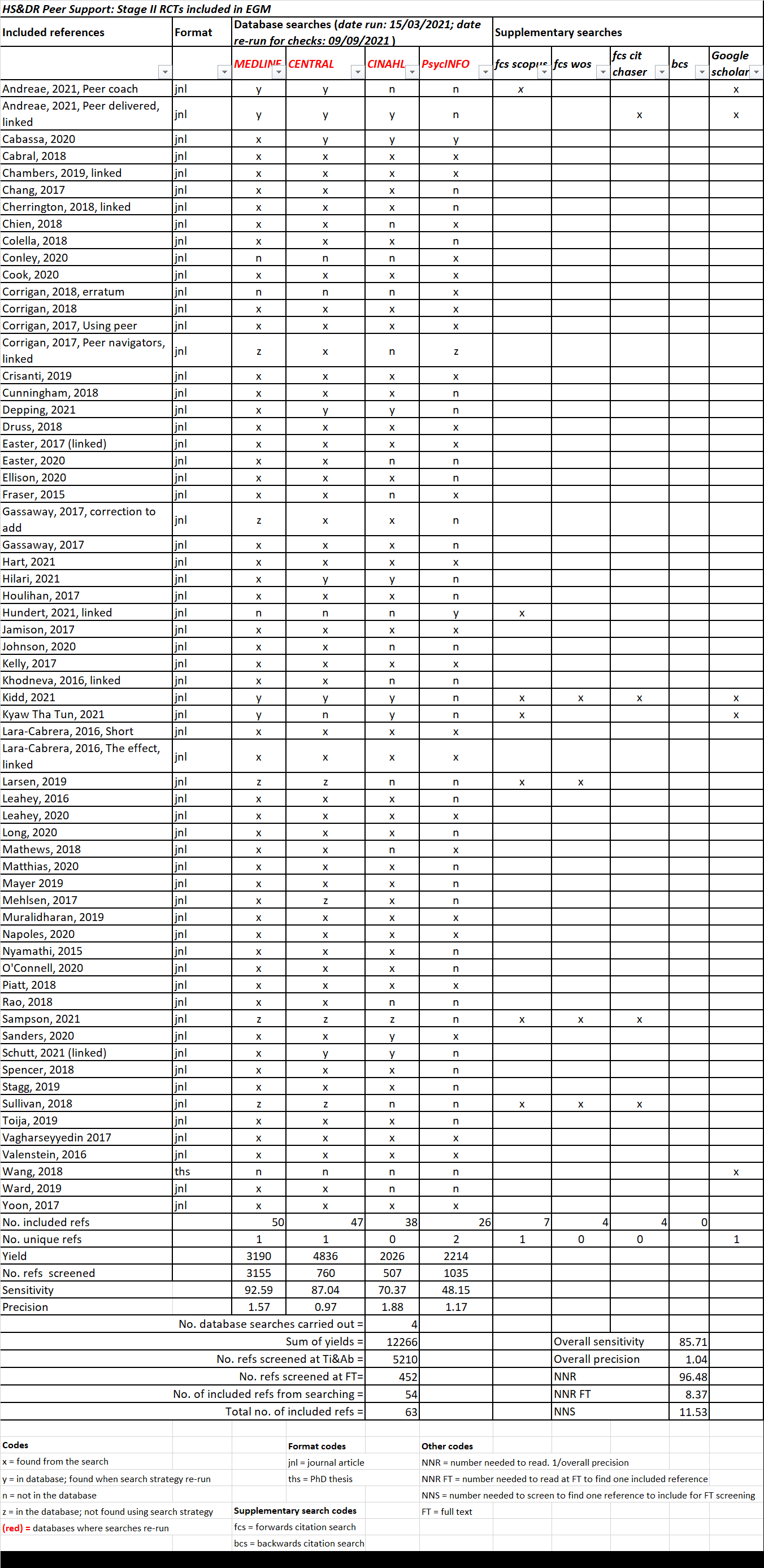
**

## 4 Data extraction forms

*indicates categories extracted only for SRs, **indicates categories extracted only for EEs; data on other categories were extracted for all studies

| **Question** | **Options** |
| --- | --- |
| Population characteristics by health/social care need | Mental health difficulties (acute); mental health difficulties (chronic); physical health difficulties (acute); physical health difficulties (chronic); addiction difficulties; parent, carers; vulnerable |
| Population age | Young people (18-25); adults (26-64); older adults (65+); not clearly defined |
| Research methods of included studies* | Mixed; qualitative; quantitative (RCT); quantitative (non RCT) |
| Quantitative synthesis methods* | Meta-analysis; narrative; other |
| Use of modelling** | Yes; no |
| Type of method** | Cost-effectiveness analysis; cost-utility analysis; cost-benefit analysis; cost-consequence analysis; comparative costing study; other (e.g. social return on investment) |
| Location | UK; Europe; USA or Canada; Australia or New Zealand; other/not specified |
| *Intervention* |  |
| Description of peer support initiative | Education, coaching, mentoring; case management, health service liaison; practical support for health behaviors; psychological, emotional, wellbeing support; self-care, self-management; social, community; not clearly defined |
| Support structure for peers | Trained; paid or contract; receive ongoing support; not specified |
| Structure of meetings | One-to-one; in groups; not specified |
| Method of contact | In person; telephone; online; not specified |
| Facilitation | Peers facilitate, not clear whether or how professionals involved; co-facilitated by peers and professionals; led by peers, working with professionals; led by professionals, working with peers; not clearly defined |
| Duration of intervention | One-off or ad-hoc; up to 3 months; over 3, up to 6 months; over 6 months, up to 12 months; more than 12 months; not specified |
| Location of intervention | Own home; medical setting; community or social location; not specified |
| Main focus of intervention | Focus on peer support alone; focus not on peer support alone |
| Assessment timeframe | At intervention end; up to 3 months; over 3, up to 6 months; over 6 months, up to 12 months; over 12 months, up to 24 months; over 24 months, up to 5 years; over 5 years, up to 10 years; lifetime; not specified |
| *Outcomes* |  |
| Health-related indicators | Physical health; mental health |
| Self-regulation | Self-management; health behaviors; addiction recovery |
| Supporting self-regulation (patient activation) | Self-efficacy; knowledge and understanding |
| Wellbeing, social connectedness | Wellbeing and quality of life; social support and relationships |
| Cost-effectiveness, service use | Cost-effectiveness; service use; employment |
| Experience of peer support | Experience/amount of support; peer outcomes |

## 5 Study quality assessment tools

**AMSTAR 2**

**critical item

|  | **Question** | **Option** |
| --- | --- | --- |
| 1. | Did the research questions and inclusion criteria for the review include the components of PICO? | Yes/no |
| 2.** | Did the report of the review contain an explicit statement that the review methods were established prior to the conduct of the review and did the report justify any significant deviations from the protocol? | Yes; partial yes; no |
| 3. | Did the review authors explain their selection of the study designs for inclusion in the review? | Yes/no |
| 4.** | Did the review authors use a comprehensive literature search strategy? | Yes; partial yes; no |
| 5. | Did the review authors perform study selection in duplicate? | Yes/no |
| 6. | Did the review authors perform data extraction in duplicate? | Yes/no |
| 7.** | Did the review authors provide a list of excluded studies and justify the exclusions? | Yes; partial yes; no |
| 8. | Did the review authors describe the included studies in adequate detail? | Yes; partial yes; no |
| 9.** | Did the review authors use a satisfactory technique for assessing the risk of bias (RoB) in individual studies that were included in the review? | Yes; partial yes; no  Includes only NRSI/ RCTs |
| 10. | Did the review authors report on the sources of funding for the studies included in the review? | Yes/no |
| 11.** | If meta-analysis was performed did the review authors use appropriate methods for statistical combination of results? | Yes/no  No meta-analysis conducted |
| 12. | If meta-analysis was performed, did the review authors assess the potential impact of RoB in individual studies on the results of the meta-analysis or other evidence synthesis? | Yes/no  No meta-analysis conducted |
| 13.** | Did the review authors account for RoB in individual studies when interpreting/ discussing the results of the review? | Yes/no |
| 14. | Did the review authors provide a satisfactory explanation for, and discussion of, any heterogeneity observed in the results of the review? | Yes/no |
| 15.** | If they performed quantitative synthesis did the review authors carry out an adequate investigation of publication bias (small study bias) and discuss its likely impact on the results of the review? | Yes/no  No meta-analysis conducted |
| 16. | Did the review authors report any potential sources of conflict of interest, including any funding they received for conducting the review? | Yes/no |

**Rating overall confidence in the results of the review**

**High** No or one non-critical weakness: the SR provides an accurate and comprehensive summary of the results of the available studies that address the question of interest

**Moderate** More than one non-critical weakness**: the SR has more than one weakness but no critical flaws. It may provide an accurate summary of the results of the available studies that were included in the review

**Low** One critical flaw with or without non-critical weaknesses: the review has a critical flaw and may not provide an accurate and comprehensive summary of the available studies that address the question of interest

**Critically low** More than one critical flaw with or without non-critical weaknesses: the review has more than one critical flaw and should not be relied on to provide an accurate and comprehensive summary of the available studies

**Multiple non-critical weaknesses may diminish confidence in the review and may mean it is appropriate to move the overall appraisal from moderate to low confidence.

**ROB**

*indicates key domains

| **Domain** | **Description** |
| --- | --- |
| *Selection bias* | |
| Random sequence generation* | Described the method used to generate the allocation sequence in sufficient detail to allow an assessment of whether it should produce comparable groups |
| Allocation concealment | Described the method used to conceal the allocation sequence in sufficient detail to determine whether intervention allocations could have been foreseen before or during enrollment |
| *Performance bias* | |
| Blinding of participants and personnel | Described all measures used, if any, to blind study participants and personnel from knowledge of which intervention a participant received. Provided information relating to whether the intended blinding was effective |
| *Detection bias* | |
| Blinding of outcome assessment | Described all measures used, if any, to blind outcome assessors from knowledge of which intervention a participant received. Provided information relating to whether the intended blinding was effective |
| *Attrition bias* | |
| Incomplete outcome data* | Described the completeness of outcome data for each main outcome, including attrition and exclusions from the analysis, the numbers in each intervention group (compared with total randomized participants), and reasons for attrition/exclusions |
| *Reporting bias* | |
| Selective reporting* | Assessment of the possibility of selective outcome reporting. |
| *Other bias* | Any important concerns about bias not addressed in the other domains in the tool (these are generally pre-specified). |

Answers for each question are used to assess the risk of bias – either high, low, or unclear – for each domain. Key domains are then used to calculate an overall risk of bias for the trial:

**Low risk** The study is judged to be at low risk of bias for all domains.

**Unclear** At least one domain is judged to be unclear for this result, but none are considered to be at high risk of bias.

**High risk** *Either* one of more domains is judged to be at high risk of bias *or* the study is considered to be unclear for multiple domains in a way that substantially lowers confidence in the result.

Review authors decide which domains are key in the context of the intervention of interest, and aims of the research (Higgins & Green, 2011). Key domains for this EGM are indicated with an asterisk in the table above.

**CHEC**

|  | **Question** | **Option** |
| --- | --- | --- |
| 1. | Is the study population clearly described? | Yes/no |
| 2. | Are competing alternatives clearly described? | Yes/no |
| 3. | Is a well-defined research question posed in answerable form? | Yes/no |
| 4. | Is the economic study design appropriate to the stated objective? | Yes/no |
| 5. | Is the chosen time horizon appropriate to include relevant costs and consequences? | Yes/no |
| 6. | Is the actual perspective chosen appropriate? | Yes/no |
| 7. | Are all important and relevant costs for each alternative identified? | Yes/no |
| 8. | Are all costs measured appropriately in physical units? | Yes/no |
| 9. | Are costs valued appropriately? | Yes/no |
| 10. | Are all important and relevant outcomes for each alternative identified? | Yes/no |
| 11. | Are all outcomes measured appropriately? | Yes/no |
| 12. | Are outcomes valued appropriately? | Yes/no |
| 13. | Is an incremental analysis of costs and outcomes of alternatives performed? | Yes/no |
| 14. | Are all future costs and outcomes discounted appropriately? | Yes/no |
| 15. | Are all important variables, whose values are uncertain, appropriately subjected to sensitivity analysis? | Yes/no |
| 16. | Do the conclusions follow from the data reported? | Yes/no |
| 17. | Does the study discuss the generalizability of the results to other settings and patient/client groups? | Yes/no |
| 18. | Does the article indicate that there is no potential conflict of interest of study researcher(s) and funder(s)? | Yes/no |
| 19. | Are ethical and distributional issues discussed appropriately? | Yes/no |

An overall assessment of quality was derived for each study by calculating the percentage of applicable questions which were answered ‘yes’ (Ahumada-Canale et al., 2019; Wijnen et al., 2017). The categories were:

**High quality** Studies scoring over 75%.

**Medium quality** Studies scoring between 50 and 74%.

**Low quality** Studies scoring below 50%.

## 6 List of pre-2015 studies

Barlow, J. H., Turner, A. P., & Gilchrist, M. (2009). A randomised controlled trial of lay-led self-management for myocardial infarction patients who have completed cardiac rehabilitation. *European Journal of Cardiovascular Nursing,* **8**(4), 293-301. https://dx.doi.org/10.1016/j.ejcnurse.2009.02.002

Carroll, D. L., & Rankin, S. H. (2006). Comparing interventions in older unpartnered adults after myocardial infarction. *European Journal of Cardiovascular Nursing,* **5**(1), 83-89.

Chaisson, R. E., Barnes, G. L., Hackman, J., Watkinson, L., Kimbrough, L., Metha, S., Cavalcante, S., & Moore, R. D. (2001). A randomized, controlled trial of interventions to improve adherence to isoniazid therapy to prevent tuberculosis in injection drug users. *American Journal of Medicine,* **110**(8), 610-615.

Chambers, S. K., Schover, L., Halford, K., Clutton, S., Ferguson, M., Gordon, L., Gardiner, R. A., Occhipinti, S., & Dunn, J. (2008). ProsCan for Couples: randomised controlled trial of a couples-based sexuality intervention for men with localised prostate cancer who receive radical prostatectomy. *BMC Cancer,* **8**, 226. https://dx.doi.org/10.1186/1471-2407-8-226

Chien, W. T., Chan, S., Morrissey, J., & Thompson, D. (2005). Effectiveness of a mutual support group for families of patients with schizophrenia. *Journal of Advanced Nursing,* **51**(6), 595-608.

Chien, W. T., & Chan, S. W. (2004). One-year follow-up of a multiple-family-group intervention for Chinese families of patients with schizophrenia. *Psychiatric Services,* **55**(11), 1276-1284.

Chien, W. T., & Chan, S. W. (2013). The effectiveness of mutual support group intervention for Chinese families of people with schizophrenia: a randomised controlled trial with 24-month follow-up. *International Journal of Nursing Studies,* **50**(10), 1326-1340. https://dx.doi.org/10.1016/j.ijnurstu.2013.01.004

Chien, W. T., Chan, S. W., & Thompson, D. R. (2006). Effects of a mutual support group for families of Chinese people with schizophrenia: 18-month follow-up. *British Journal of Psychiatry,* **189**, 41-49.

Chien, W. T., Norman, I., & Thompson, D. R. (2004). A randomized controlled trial of a mutual support group for family caregivers of patients with schizophrenia. *International Journal of Nursing Studies,* **41**(6), 637-649.

Chien, W. T., & Thompson, D. R. (2013). An RCT with three-year follow-up of peer support groups for Chinese families of persons with schizophrenia. *Psychiatric Services,* **64**(10), 997-1005. https://dx.doi.org/10.1176/appi.ps.201200243

Chien, W. T., Thompson, D. R., & Norman, I. (2008). Evaluation of a peer-led mutual support group for Chinese families of people with schizophrenia. *American Journal of Community Psychology,* **42**(1-2), 122-134. https://dx.doi.org/10.1007/s10464-008-9178-8

Colella, T. J. F. (2009). *The effect of a professionally-guided telephone peer support intervention on early recovery outcomes in men following coronary artery bypass graft surgery.* University of Calgary (Canada),

Cook, J. A., Copeland, M. E., Floyd, C. B., Jonikas, J. A., Hamilton, M. M., Razzano, L., Carter, T. M., Hudson, W. B., Grey, D. D., & Boyd, S. (2012). A randomized controlled trial of effects of Wellness Recovery Action Planning on depression, anxiety, and recovery. *Psychiatric Services,* **63**(6), 541-547. https://dx.doi.org/10.1176/appi.ps.201100125

Cook, J. A., Jonikas, J. A., Hamilton, M. M., Goldrick, V., Steigman, P. J., Grey, D. D., Burke, L., Carter, T. M., Razzano, L. A., & Copeland, M. E. (2013). Impact of Wellness Recovery Action Planning on service utilization and need in a randomized controlled trial. *Psychiatric Rehabilitation Journal,* **36**(4), 250-257. https://dx.doi.org/10.1037/prj0000028

Dale, J., Caramlau, I., Docherty, A., Sturt, J., & Hearnshaw, H. (2007). Telecare motivational interviewing for diabetes patient education and support: a randomised controlled trial based in primary care comparing nurse and peer supporter delivery. *Trials,* **8**, 18.

Davidson, L., Shahar, G., Stayner, D. A., Chinman, M. J., Rakfeldt, J., & Tebes, J. K. (2004). Supported socialization for people with psychiatric disabilities: Lessons from a randomized controlled trial. *Journal of Community Psychology,* **32**(4), 453-477. https://dx.doi.org/10.1002/jcop.20013

Dixon, L. B., Lucksted, A., Medoff, D. R., Burland, J., Stewart, B., Lehman, A. F., Fang, L. J., Sturm, V., Brown, C., & Murray-Swank, A. (2011). Outcomes of a randomized study of a peer-taught family-to-family education program for mental illness. *Psychiatric Services,* **62**(6), 591-597. https://dx.doi.org/10.1176/ps.62.6.pss6206_0591

Dukhovny, D., Dennis, C. L., Hodnett, E., Weston, J., Stewart, D. E., Mao, W., & Zupancic, J. A. (2013). Prospective economic evaluation of a peer support intervention for prevention of postpartum depression among high-risk women in Ontario, Canada. *American Journal of Perinatology,* **30**(8), 631-642. https://dx.doi.org/10.1055/s-0032-1331029

Fogarty, L. A., Heilig, C. M., Armstrong, K., Cabral, R., Galavotti, C., Gielen, A. C., & Green, B. M. (2001). Long-term effectiveness of a peer-based intervention to promote condom and contraceptive use among HIV-positive and at-risk women. *Public Health Reports,* **116**(Suppl 1), 103-119.

Frost, R. O., Ruby, D., & Shuer, L. J. (2012). The Buried in Treasures Workshop: waitlist control trial of facilitated support groups for hoarding. *Behaviour Research and Therapy,* **50**(11), 661-667. https://dx.doi.org/10.1016/j.brat.2012.08.004

Fung, W., & Chien, W. (2002). The effectiveness of a mutual support group for family caregivers of a relative with dementia. *Archives of Psychiatric Nursing,* **16**(3), 134‐144. https://dx.doi.org/10.1053/apnu.2002.32951

Gielen, A. C., Fogarty, L. A., Armstrong, K., Green, B. M., Cabral, R., Milstein, B., Galavotti, C., & Heilig, C. M. (2001). Promoting condom use with main partners: a behavioral intervention trial for women. *AIDS and Behavior,* **5**(3), 193-204.

Gillespie, P., O'Shea, E., Paul, G., O'Dowd, T., & Smith, S. M. (2012). Cost effectiveness of peer support for type 2 diabetes. *International Journal of Technology Assessment in Health Care,* **28**(1), 3-11. https://dx.doi.org/10.1017/S0266462311000663

Hassouneh, D., Nguyen, T., Chen, Z., & McNeff, E. (2013). Healing pathways: a program for women with physical disabilities and depression. *Rehabilitation Research & Practice,* **2013**, 649875. https://dx.doi.org/10.1155/2013/649875

Hirsch-Moverman, Y. (2011). TB or not TB: Treatment of latent tuberculosis infection in Harlem, New York. *Dissertation Abstracts International: Section B: The Sciences and Engineering,* **72**(6-B), 3321.

Hirsch-Moverman, Y., Colson, P. W., Bethel, J., Franks, J., & El-Sadr, W. M. (2013). Can a peer-based intervention impact adherence to the treatment of latent tuberculous infection? *International Journal of Tuberculosis & Lung Disease,* **17**(9), 1178-1185. https://dx.doi.org/10.5588/ijtld.12.0823

Jerant, A., Moore-Hill, M., & Franks, P. (2009). Home-based, peer-led chronic illness self-management training: findings from a 1-year randomized controlled trial. *Annals of Family Medicine,* **7**(4), 319-327. https://dx.doi.org/10.1370/afm.996

Kronish, I. M., Goldfinger, J. Z., Negron, R., Fei, K., Tuhrim, S., Arniella, G., & Horowitz, C. R. (2014). Effect of peer education on stroke prevention: the prevent recurrence of all inner-city strokes through education randomized controlled trial. *Stroke,* **45**(11), 3330-3336. https://dx.doi.org/10.1161/STROKEAHA.114.006623

Landers, G., & Zhou, M. (2014). The impact of Medicaid peer support utilization on cost. *Medicare & Medicaid Research Review,* **4**(1). https://dx.doi.org/10.5600/mmrr.004.01.a04

Linas, B. P., Barter, D. M., Leff, J. A., Assoumou, S. A., Salomon, J. A., Weinstein, M. C., Kim, A. Y., & Schackman, B. R. (2014). The hepatitis C cascade of care: identifying priorities to improve clinical outcomes. *PLoS One,* **9**(5), e97317. https://dx.doi.org/10.1371/journal.pone.0097317

Lorig, K., Feigenbaum, P., Regan, C., Ung, E., Chastain, R. L., & Holman, H. R. (1986). A comparison of lay-taught and professional-taught arthritis self-management courses. *Journal of Rheumatology,* **13**(4), 763-767.

McKirnan, D. J., Tolou-Shams, M., & Courtenay-Quirk, C. (2010). The Treatment Advocacy Program: a randomized controlled trial of a peer-led safer sex intervention for HIV-infected men who have sex with men. *Journal of Consulting & Clinical Psychology,* **78**(6), 952-963. https://dx.doi.org/10.1037/a0020759

Merelle, S., Sorbi, M., van Doornen, L., & Passchier, J. (2008). Migraine patients as trainers of their fellow patients in non-pharmacological preventive attack management: Short-term effects of a randomized controlled trial. *Cephalalgia,* **28**(2), 127-138.

Partridge, M. R., Caress, A. L., Brown, C., Hennings, J., Luker, K., Woodcock, A., & Campbell, M. (2008). Can lay people deliver asthma self-management education as effectively as primary care based practice nurses? *Thorax,* **63**(9), 778-783. https://dx.doi.org/10.1136/thx.2007.084251

Perlick, D. A., Nelson, A. H., Mattias, K., Selzer, J., Kalvin, C., Wilber, C. H., Huntington, B., Holman, C. S., & Corrigan, P. W. (2011). In our own voice-family companion: reducing self-stigma of family members of persons with serious mental illness. *Psychiatric Services,* **62**(12), 1456-1462. https://dx.doi.org/10.1176/appi.ps.001222011

Perry, E., Swartz, J., Brown, S., Smith, D., Kelly, G., & Swartz, R. (2005). Peer mentoring: a culturally sensitive approach to end-of-life planning for long-term dialysis patients. *American Journal of Kidney Diseases,* **46**(1), 111-119.

Pillemer, K., & Suitor, J. J. (2002). Peer support for Alzheimer's caregivers: is it enough to make a difference? *Research on Aging,* **24**(2), 171-192. https://dx.doi.org/10.1177/0164027502242001

Pratt, C. W., Lu, W., Swarbrick, M., & Murphy, A. (2011). Selective provision of illness management and recovery modules. *American Journal of Psychiatric Rehabilitation,* **14**(4), 245‐258. https://dx.doi.org/10.1080/15487768.2011.622133

Riegel, B., & Carlson, B. (2004). Is individual peer support a promising intervention for persons with heart failure? *Journal of Cardiovascular Nursing,* **19**(3), 174-183.

Roberts, N. J., Boyd, K. A., Briggs, A. H., Caress, A. L., & Partridge, M. R. (2012). Nurse led versus lay educators support for those with asthma in primary care: a costing study. *BMC Pulmonary Medicine,* **12**, 52. https://dx.doi.org/10.1186/1471-2466-12-52

Rohr, F., Munier, A., Sullivan, D., Bailey, I., Gennaccaro, M., Levy, H., Brereton, H., Gleason, S., Goss, B., Lesperance, E., Moseley, K., Singh, R., Tonyes, L., Vespa, H., & Waisbren, S. (2004). The Resource Mothers Study of Maternal Phenylketonuria: preliminary findings. *Journal of Inherited Metabolic Disease,* **27**(2), 145-155.

Rothrock, J. F., Parada, V. A., Sims, C., Key, K., Walters, N. S., & Zweifler, R. M. (2006). The impact of intensive patient education on clinical outcome in a clinic-based migraine population. *Headache,* **46**(5), 726-731.

Savelkoul, M., & de Witte, L. P. (2004). Mutual support groups in rheumatic diseases: Effects and participants' perceptions. *Arthritis & Rheumatism,* **51**(4), 605-608.

Savelkoul, M., de Witte, L. P., Candel, M. J., van der Tempel, H., & van den Borne, B. (2001). Effects of a coping intervention on patients with rheumatic diseases: results of a randomized controlled trial. *Arthritis & Rheumatism,* **45**(1), 69-76.

Schwartz, C. E. (1999). Teaching coping skills enhances quality of life more than peer support: results of a randomized trial with multiple sclerosis patients. *Health Psychology,* **18**(3), 211-220.

Strong, L. L., Von Korff, M., Saunders, K., & Moore, J. E. (2006). Cost-effectiveness of two self-care interventions to reduce disability associated with back pain. *Spine,* **31**(15), 1639-1645.

Sullivan, C., Leon, J. B., Sayre, S. S., Marbury, M., Ivers, M., Pencak, J. A., Bodziak, K. A., Hricik, D. E., Morrison, E. J., Albert, J. M., Navaneethan, S. D., Reyes, C. M., & Sehgal, A. R. (2012). Impact of navigators on completion of steps in the kidney transplant process: a randomized, controlled trial. *Clinical Journal of the American Society of Nephrology,* **7**(10), 1639-1645. https://dx.doi.org/10.2215/CJN.11731111

Taylor, R. R., Jason, L. A., Shiraishi, Y., Schoeny, M. E., & Keller, J. (2006). Conservation of resources theory, perceived stress, and chronic fatigue syndrome: outcomes of a consumer-driven rehabilitation program. *Rehabilitation Psychology,* **51**(2), 157-165. https://dx.doi.org/10.1037/0090-5550.51.2.157

Toseland, R. W., Rossiter, C. M., & Labrecque, M. S. (1989). The effectiveness of three group intervention strategies to support family caregivers. *American Journal of Orthopsychiatry,* **59**(3), 420‐429.

Tulsky, J. P., Pilote, L., Hahn, J. A., Zolopa, A. J., Burke, M., Chesney, M., & Moss, A. R. (2000). Adherence to isoniazid prophylaxis in the homeless: a randomized controlled trial. *Archives of Internal Medicine,* **160**(5), 697-702.

Turner, B. J., Weiner, M., Berry, S. D., Lillie, K., Fosnocht, K., & Hollenbeak, C. S. (2008). Overcoming poor attendance to first scheduled colonoscopy: a randomized trial of peer coach or brochure support. *Journal of General Internal Medicine,* **23**(1), 58-63.

van Gestel-Timmermans, J. A. W. M., Brouwers, E. P. M., van Assen, M. A. L. M., & van Nieuwenhuizen, C. (2011). *Chapter 4: Effects of a peer-run course on the recovery of people with major psychiatric problems: a randomised controlled trial. In: Recovery is up to you: evaluation of a peer-run course.* Tilburg University, Tilburg, The Netherlands.

Von Korff, M., Moore, J. E., Lorig, K., Cherkin, D. C., Saunders, K., Gonzalez, V. M., Laurent, D., Rutter, C., & Comite, F. (1998). A randomized trial of a lay person-led self-management group intervention for back pain patients in primary care. *Spine,* **23**(23), 2608-2615.

White, V. M., Young, M. A., Farrelly, A., Meiser, B., Jefford, M., Williamson, E., Ieropoli, S., Duffy, J., & Winship, I. (2014). Randomized controlled trial of a telephone-based peer-support program for women carrying a BRCA1 or BRCA2 mutation: impact on psychological distress. *Journal of Clinical Oncology,* **32**(36), 4073-4080. https://dx.doi.org/10.1200/JCO.2013.54.1607

Yates, B. T., Mannix, D., Freed, M. C., Campbell, J., Johnsen, M., Jones, K., & Blyler, C. R. (2011). Consumer-Operated Service Programs: monetary and donated costs and cost-effectiveness. *Psychiatric Rehabilitation Journal,* **35**(2), 91-99. https://dx.doi.org/10.2975/35.2.2011.91.99

## 7 List of impact evaluations already included in SRs

Aben, L., Heijenbrok-Kal, M. H., Ponds, R. W., Busschbach, J. J., & Ribbers, G. M. (2014). Long-lasting effects of a new memory self-efficacy training for stroke patients: a randomized controlled trial. *Neurorehabilitation and Neural Repair,* **28**(3), 199-206. https://dx.doi.org/10.1177/1545968313478487

Aben, L., Heijenbrok-Kal, M. H., van Loon, E. M. P., Groet, E., Ponds, R. W. H. M., Busschbach, J. J. V., & Ribbers, G. M. (2012). Training memory self-efficacy in the chronic stage after stroke: A randomized controlled trial. *Neurorehabilitation and Neural Repair,* **27**(2), 110-117. https://dx.doi.org/10.1177/1545968312455222

Altice, F. L., Maru, D. S. R., Bruce, R. D., Springer, S. A., & Friedland, G. H. (2007). Superiority of directly administered antiretroviral therapy over self-administered therapy among HIV-infected drug users: A prospective, randomized, controlled trial. *Clinical Infectious Diseases,* **45**(6), 770-778. https://dx.doi.org/10.1086/521166

Andersson, G., Bergstrom, J., Hollandare, F., Carlbring, P., Kaldo, V., & Ekselius, L. (2005). Internet-based self-help for depression: randomised controlled trial. *British Journal of Psychiatry,* **187**, 456-461. https://dx.doi.org/10.1192/bjp.187.5.456

Ayala, G. X., Ibarra, L., Cherrington, A. L., Parada, H., Horton, L., Ji, M., & Elder, J. P. (2015). Puentes hacia una mejor vida (Bridges to a Better Life): Outcome of a diabetes control peer support intervention. *Annals of Family Medicine,* **13**(Suppl 1), S9-17. https://dx.doi.org/10.1370/afm.1807

Backhaus, S., Ibarra, S., Parrott, D., & Malec, J. (2016). Comparison of a cognitive-behavioral coping skills group to a peer support group in a brain injury population. *Archives of Physical Medicine and Rehabilitation,* **97**(2), 281-291. https://dx.doi.org/10.1016/j.apmr.2015.10.097

Baghianimoghadam, M. H., Hadavandkhani, M., Mohammadi, M., Fallahzade, H., & Baghianimoghadam, B. (2012). Current education versus peer-education on walking in type 2 diabetic patients based on Health Belief Model: a randomized control trial study. *Romanian Journal of Internal Medicine,* **50**(2), 165-172.

Baksi, A. K., Al-Mrayat, M., Hogan, D., Whittingstall, E., Wilson, P., & Wex, J. (2008). Peer advisers compared with specialist health professionals in delivering a training programme on self-management to people with diabetes: a randomized controlled trial. *Diabetic Medicine,* **25**(9), 1076-1082. https://dx.doi.org/10.1111/j.1464-5491.2008.02542.x

Barbic, S., Krupa, T., & Armstrong, I. (2009). A randomized controlled trial of the effectiveness of a modified recovery workbook program: Preliminary findings. *Psychiatric Services,* **60**(4), 491-497. https://dx.doi.org/10.1176/ps.2009.60.4.491

Barlow, J., Turner, A., Edwards, R., & Gilchrist, M. (2009). A randomised controlled trial of lay-led self-management for people with multiple sclerosis. *Patient Education and Counseling,* **77**(1), 81-89. https://dx.doi.org/10.1016/j.pec.2009.02.009

Barlow, J. H., Turner, A. P., & Wright, C. C. (2000). A randomized controlled study of the Arthritis Self-Management Programme in the UK. *Health Education Research,* **15**(6), 665-680. https://dx.doi.org/10.1093/her/15.6.665

Berg, K. M., Litwin, A., Li, X., Heo, M., & Arnsten, J. H. (2011). Directly observed antiretroviral therapy improves adherence and viral load in drug users attending methadone maintenance clinics: A randomized controlled trial. *Drug and Alcohol Dependence,* **113**(2-3), 192-199. https://dx.doi.org/10.1016/j.drugalcdep.2010.07.025

Bernstein, J., Bernstein, E., Tassiopoulos, K., Heeren, T., Levenson, S., & Hingson, R. (2005). Brief motivational intervention at a clinic visit reduces cocaine and heroin use. *Drug and Alcohol Dependence,* **77**(1), 49-59. https://dx.doi.org/10.1016/j.drugalcdep.2004.07.006

Berrien, V. M., Salazar, J. C., Reynolds, E., & McKay, K. (2004). Adherence to antiretroviral therapy in HIV-infected pediatric patients improves with home-based intensive nursing intervention. *AIDS Patient Care and STDs,* **18**(6), 355-363. https://dx.doi.org/10.1089/1087291041444078

Best, K. L., Miller, W. C., Huston, G., Routhier, F., & Eng, J. J. (2016). Pilot study of a peer-led wheelchair training program to improve self-efficacy using a manual wheelchair: A randomized controlled trial. *Archives of Physical Medicine and Rehabilitation,* **97**(1), 37-44. https://dx.doi.org/10.1016/j.apmr.2015.08.425

Blondell, R. D., Frydrych, L. M., Jaanimagi, U., Ashrafioun, L., Homish, G. G., Foschio, E. M., & Bashaw, H. L. (2011). A randomized trial of two behavioral interventions to improve outcomes following inpatient detoxification for alcohol dependence. *Journal of Addictive Diseases,* **30**(2), 136-148. https://dx.doi.org/10.1080/10550887.2011.554777

Boevink, W., Kroon, H., van Vugt, M., Delespaul, P., & van Os, J. (2016). A user-developed, user run recovery programme for people with severe mental illness: A randomised control trial. *Psychosis-Psychological Social and Integrative Approaches,* **8**(4), 287-300. https://dx.doi.org/10.1080/17522439.2016.1172335

Bogenschutz, M. P., Rice, S. L., Tonigan, J. S., Vogel, H. S., Nowinski, J., Hume, D., & Arenella, P. B. (2014). 12-step facilitation for the dually diagnosed: a randomized clinical trial. *Journal of Substance Abuse Treatment,* **46**(4), 403-411. https://dx.doi.org/10.1016/j.jsat.2013.12.009

Bowen, S., Witkiewitz, K., Clifasefi, S. L., Grow, J., Chawla, N., Hsu, S. H., Carroll, H. A., Harrop, E., Collins, S. E., Lustyk, M. K., & Larimer, M. E. (2014). Relative efficacy of mindfulness-based relapse prevention, standard relapse prevention, and treatment as usual for substance use disorders: a randomized clinical trial. *JAMA Psychiatry,* **71**(5), 547-556. https://dx.doi.org/10.1001/jamapsychiatry.2013.4546

Brashers, D. E., Basinger, E. D., Rintamaki, L. S., Caughlin, J. P., & Para, M. (2017). Taking control: The efficacy and durability of a peer-led uncertainty management intervention for people recently diagnosed with HIV. *Health Communication,* **32**(1), 11-21. https://dx.doi.org/10.1080/10410236.2015.1089469

Bright, J. I., Baker, K. D., & Neimeyer, R. A. (1999). Professional and paraprofessional group treatments for depression: a comparison of cognitive-behavioral and mutual support interventions. *Journal of Consulting and Clinical Psychology,* **67**(4), 491-501. https://dx.doi.org/10.1037/0022-006X.67.4.491

Brown, T. G., Seraganian, P., Tremblay, J., & Annis, H. (2002). Process and outcome changes with relapse prevention versus 12-Step aftercare programs for substance abusers. *Addiction,* **97**(6), 677-689. https://dx.doi.org/10.1046/j.1360-0443.2002.00101.x

Bultz, B. D., Speca, M., Brasher, P. M., Geggie, P. H., & Page, S. A. (2000). A randomized controlled trial of a brief psychoeducational support group for partners of early stage breast cancer patients. *Psychooncology,* **9**(4), 303-313. https://dx.doi.org/10.1002/1099-1611(200007/08)9:4<303::aid-pon462>3.0.co;2-m

Buman, M. P., Giacobbi, P. R., Dzierzewski, J. M., Morgan, A. A., McCrae, C. S., Roberts, B. L., & Marsiske, M. (2011). Peer volunteers improve long-term maintenance of physical activity with older adults: A randomized controlled trial. *Journal of Physical Activity & Health,* **8**, S257-S266. https://dx.doi.org/10.1123/jpah.8.s2.s257

Cade, J. E., Kirk, S. F. L., Nelson, P., Hollins, L., Deakin, T., Greenwood, D. C., & Harvey, E. L. (2009). Can peer educators influence healthy eating in people with diabetes? Results of a randomized controlled trial. *Diabetic Medicine,* **26**(10), 1048-1054. https://doi.org/10.1111/j.1464-5491.2009.02808.x

Cadilhac, D. A., Hoffmann, S., Kilkenny, M., Lindley, R., Lalor, E., Osborne, R. H., & Batterbsy, M. (2011). A phase II multicentered, single-blind, randomized, controlled trial of the stroke self-management program. *Stroke,* **42**(6), 1673-1679. https://dx.doi.org/10.1161/strokeaha.110.601997

Campbell, L. C., Keefe, F. J., Scipio, C., McKee, D. C., Edwards, C. L., Herman, S. H., Johnson, L. E., Colvin, O. M., McBride, C. M., & Donatucci, C. (2007). Facilitating research participation and improving quality of life for African American prostate cancer survivors and their intimate partners. A pilot study of telephone-based coping skills training. *Cancer,* **109**(2 Suppl), 414-424. https://dx.doi.org/10.1002/cncr.22355

Campbell, M. K., James, A., Hudson, M. A., Carr, C., Jackson, E., Oates, V., Demissie, S., Farrell, D., & Tessaro, I. (2004). Improving multiple behaviors for colorectal cancer prevention among African American church members. *Health Psychology,* **23**(5), 492-502. https://dx.doi.org/10.1037/0278-6133.23.5.492

Castelein, S. (2008). The effectiveness of support groups for people suffering from psychosis: a randomised controlled trial. Retrieved from www.isrctn.com/ISRCTN02457313

Castelein, S., Bruggeman, R., Van Busschbach, J. T., Van Der Gaag, M., Stant, A. D., Knegtering, H., & Wiersma, D. (2008). 46 – The effectiveness of minimally guided peer support groups for people suffering from psychosis: A randomized controlled trial. *Schizophrenia Research,* **98**, 52-53. https://doi.org/10.1016/j.schres.2007.12.113

Castelein, S., Bruggeman, R., van Busschbach, J. T., van der Gaag, M., Stant, A. D., Knegtering, H., & Wiersma, D. (2008). The effectiveness of peer support groups in psychosis: a randomized controlled trial. *Acta Psychiatrica Scandinavica,* **118**(1), 64-72. https://dx.doi.org/10.1111/j.1600-0447.2008.01216.x

Chambers, S. K., Occhipinti, S., Schover, L., Nielsen, L., Zajdlewicz, L., Clutton, S., Halford, K., Gardiner, R. A., & Dunn, J. (2015). A randomised controlled trial of a couples-based sexuality intervention for men with localised prostate cancer and their female partners. *Psychooncology,* **24**(7), 748-756. https://dx.doi.org/10.1002/pon.3726

Chan, J. C., Sui, Y., Oldenburg, B., Zhang, Y., Chung, H. H., Goggins, W., Au, S., Brown, N., Ozaki, R., Wong, R. Y., Ko, G. T., & Fisher, E. (2014). Effects of telephone-based peer support in patients with type 2 diabetes mellitus receiving integrated care: a randomized clinical trial. *JAMA Internal Medicine,* **174**(6), 972-981. https://dx.doi.org/10.1001/jamainternmed.2014.655

Chang, L. W., Kagaayi, J., Nakigozi, G., Ssempijja, V., Packer, A. H., Serwadda, D., Quinn, T. C., Gray, R. H., Bollinger, R. C., & Reynolds, S. J. (2010). Effect of peer health workers on AIDS care in Rakai, Uganda: a cluster-randomized trial. *PLoS One,* **5**(6), e10923. https://dx.doi.org/10.1371/journal.pone.0010923

Chen, C.-H., Tseng, Y.-F., Chou, F.-H., & Wang, S.-Y. (2000). Effects of support group intervention in postnatally distressed women: A controlled study in Taiwan. *Journal of Psychosomatic Research,* **49**(6), 395-399. https://doi.org/10.1016/S0022-3999(00)00180-X

Chinman, M., Oberman, R. S., Hanusa, B. H., Cohen, A. N., Salyers, M. P., Twamley, E. W., & Young, A. S. (2015). A cluster randomized trial of adding peer specialists to intensive case management teams in the Veterans Health Administration. *Journal of Behavioral Health Services Research,* **42**(1), 109-121. https://dx.doi.org/10.1007/s11414-013-9343-1

Clarke, G. N., Herinckx, H. A., Kinney, R. F., Paulson, R. I., Cutler, D. L., Lewis, K., & Oxman, E. (2000). Psychiatric hospitalizations, arrests, emergency room visits, and homelessness of clients with serious and persistent mental illness: findings from a randomized trial of two ACT programs vs. usual care. *Mental Health Services Research,* **2**(3), 155-164. https://dx.doi.org/10.1023/a:1010141826867

Cook, J. A., Copeland, M. E., Jonikas, J. A., Hamilton, M. M., Razzano, L. A., Grey, D. D., Floyd, C. B., Hudson, W. B., Macfarlane, R. T., Carter, T. M., & Boyd, S. (2012). Results of a randomized controlled trial of mental illness self-management using Wellness Recovery Action Planning. *Schizophrenia Bulletin,* **38**(4), 881-891. https://dx.doi.org/10.1093/schbul/sbr012

Cook, J. A., Steigman, P., Pickett, S., Diehl, S., Fox, A., Shipley, P., MacFarlane, R., Grey, D. D., & Burke-Miller, J. K. (2012). Randomized controlled trial of peer-led recovery education using Building Recovery of Individual Dreams and Goals through Education and Support (BRIDGES). *Schizophrenia Research,* **136**(1-3), 36-42. https://dx.doi.org/10.1016/j.schres.2011.10.016

Craig, T., Doherty, I., Jamieson-Craig, R., Boocock, A., & Attafua, G. (2004). The consumer-employee as a member of a Mental Health Assertive Outreach Team. I. Clinical and social outcomes. *Journal of Mental Health,* **13**(1), 59-69. https://dx.doi.org/10.1080/09638230410001654567

Crane-Okada, R., Freeman, E., Kiger, H., Ross, M., Elashoff, D., Deacon, L., & Giuliano, A. E. (2012). Senior peer counseling by telephone for psychosocial support after breast cancer surgery: effects at six months. *Oncology Nursing Forum,* **39**(1), 78-89. https://dx.doi.org/10.1188/12.Onf.78-89

Dale, J., Caramlau, I., Sturt, J., Friede, T., & Walker, R. (2009). Telephone peer-delivered intervention for diabetes motivation and support: The telecare exploratory RCT. *Patient Education and Counseling,* **75**(1), 91-98. https://dx.doi.org/10.1016/j.pec.2008.09.014

Dang, T. T. N., Deoisres, W., Keeratiyutawong, P., & Baumann, L. (2013). Effectiveness of a diabetes self management support intervention in Vietnamese adults with type 2 diabetes. *Journal of Science, Technology and Humanities,* **11**, 13-23.

Davis, W. T., Campbell, L., Tax, J., & Lieber, C. S. (2002). A trial of "standard" outpatient alcoholism treatment vs. a minimal treatment control. *Journal of Substance Abuse Treatment,* **23**(1), 9-19. https://dx.doi.org/10.1016/s0740-5472(02)00227-1

Debussche, X., Besancon, S., Balcou-Debussche, M., Ferdynus, C., Delisle, H., Huiart, L., & Sidibe, A. T. (2018). Structured peer-led diabetes self-management and support in a low-income country: The ST2EP randomised controlled trial in Mali. *PLoS One,* **13**(1), e0191262. https://dx.doi.org/10.1371/journal.pone.0191262

Deng, K., Ren, Y., Luo, Z., Du, K., Zhang, X., & Zhang, Q. (2016). Peer support training improved the glycemic control, insulin management, and diabetic behaviors of patients with type 2 diabetes in rural communities of central China: A randomized controlled trial. *Medical Science Monitor,* **22**, 267-275. https://dx.doi.org/10.12659/msm.895593

Dennis, C. L. (2003). The effect of peer support on postpartum depression: a pilot randomized controlled trial. *Canadian Journal of Psychiatry,* **48**(2), 115-124. https://dx.doi.org/10.1177/070674370304800209

Dennis, C. L., Hodnett, E., Kenton, L., Weston, J., Zupancic, J., Stewart, D. E., & Kiss, A. (2009). Effect of peer support on prevention of postnatal depression among high risk women: multisite randomised controlled trial. *BMJ,* **338**, a3064. https://dx.doi.org/10.1136/bmj.a3064

Druss, B. G., Von Esenwein, S. A., Compton, M. T., Rask, K. J., Zhao, L., & Parker, R. M. (2010). A randomized trial of medical care management for community mental health settings: The primary care access, referral, and evaluation (PCARE) study. *American Journal of Psychiatry,* **167**(2), 151-159. https://dx.doi.org/10.1176/appi.ajp.2009.09050691

Druss, B. G., Zhao, L., von Esenwein, S. A., Bona, J. R., Fricks, L., Jenkins-Tucker, S., Sterling, E., DiClemente, R., & Lorig, K. (2010). The Health and Recovery Peer (HARP) Program: A peer-led intervention to improve medical self-management for persons with serious mental illness. *Schizophrenia Research,* **118**(1-3), 264-270. https://dx.doi.org/10.1016/j.schres.2010.01.026

Duggan, C., Carosso, E., Mariscal, N., Islas, I., Ibarra, G., Holte, S., Copeland, W., Linde, S., & Thompson, B. (2014). Diabetes prevention in Hispanics: Report from a randomized controlled trial. *Preventing Chronic Disease,* **11**(2), E28. https://dx.doi.org/10.5888/pcd11.130119

Edwards, H., Courtney, M., Finlayson, K., Shuter, P., & Lindsay, E. (2009). A randomised controlled trial of a community nursing intervention: improved quality of life and healing for clients with chronic leg ulcers. *Journal of Clinical Nursing,* **18**(11), 1541-1549. https://dx.doi.org/10.1111/j.1365-2702.2008.02648.x

Eisen, S. V., Schultz, M. R., Mueller, L. N., Degenhart, C., Clark, J. A., Resnick, S. G., Christiansen, C. L., Armstrong, M., Bottonari, K. A., Rosenheck, R. A., & Sadow, D. (2012). Outcome of a randomized study of a mental health peer education and support group in the VA. *Psychiatric Services,* **63**(12), 1243-1246. https://dx.doi.org/10.1176/appi.ps.201100348

Ellis, L. A., Campbell, A. J., & Sethi, S. (2011). Comparative randomized trial of an online cognitive-behavioral therapy program and an online support group for depression and anxiety. *Journal of CyberTherapy and Rehabilitation,* **4**(4), 461-467.

Enriquez, M., Cheng, A. L., Banderas, J., Farnan, R., Chertoff, K., Hayes, D., Ortego, G., Moreno, J., Peterson, J., & McKinsey, D. (2015). A peer-led HIV medication adherence intervention targeting adults linked to medical care but without a suppressed viral load. *Journal of the International Association of Providers of AIDS Care,* **14**(5), 441-448. https://dx.doi.org/10.1177/2325957414558301

Fu, D. B., Hua, F., McGowan, P., Shen, Y. E., Zhu, L. H., Yang, H. Q., Mao, J. Q., Zhu, S. T., Ding, Y. M., & Wei, Z. H. (2003). Implementation and quantitative evaluation of chronic disease self-management programme in Shanghai, China: randomized controlled trial. *Bulletin of the World Health Organization,* **81**(3), 174-182.

Fuhr, D. C., Weobong, B., Lazarus, A., Vanobberghen, F., Weiss, H. A., Singla, D. R., Tabana, H., Afonso, E., De Sa, A., D'Souza, E., Joshi, A., Korgaonkar, P., Krishna, R., Price, L. N., Rahman, A., & Patel, V. (2019). Delivering the Thinking Healthy Programme for perinatal depression through peers: an individually randomised controlled trial in India. *Lancet Psychiatry,* **6**(2), 115-127. https://dx.doi.org/10.1016/s2215-0366(18)30466-8

Gagliardino, J. J., Arrechea, V., Assad, D., Gagliardino, G. G., Gonzalez, L., Lucero, S., Rizzuti, L., Zufriategui, Z., & Clark, C., Jr. (2013). Type 2 diabetes patients educated by other patients perform at least as well as patients trained by professionals. *Diabetes Metabolism Research and Reviews,* **29**(2), 152-160. https://dx.doi.org/10.1002/dmrr.2368

Garrett, N., Hageman, C. M., Sibley, S. D., Davern, M., Berger, M., Brunzell, C., Malecha, K., & Richards, S. W. (2005). The effectiveness of an interactive small group diabetes intervention in improving knowledge, feeling of control, and behavior. *Health Promotion Practice,* **6**(3), 320-328. https://dx.doi.org/10.1177/1524839903260846

Giese-Davis, J., Bliss-Isberg, C., Wittenberg, L., White, J., Star, P., Zhong, L., Cordova, M. J., Houston, D., & Spiegel, D. (2016). Peer-counseling for women newly diagnosed with breast cancer: A randomized community/research collaboration trial. *Cancer,* **122**(15), 2408-2417. https://dx.doi.org/10.1002/cncr.30036

Gifford, A. L., Laurent, D. D., Gonzales, V. M., Chesney, M. A., & Lorig, K. R. (1998). Pilot randomized trial of education to improve self-management skills of men with symptomatic HIV/AIDS. *Journal of Acquired Immune Deficiency Syndromes & Human Retrovirology,* **18**(2), 136-144. https://dx.doi.org/10.1097/00042560-199806010-00005

Giordano, T. P., Cully, J., Amico, K. R., Davila, J. A., Kallen, M. A., Hartman, C., Wear, J., Buscher, A., & Stanley, M. (2016). A randomized trial to test a peer mentor intervention to improve outcomes in persons hospitalized with HIV infection. *Clinical Infectious Diseases,* **63**(5), 678-686. https://dx.doi.org/10.1093/cid/ciw322

Gjerdingen, D. K., McGovern, P., Pratt, R., Johnson, L., & Crow, S. (2013). Postpartum doula and peer telephone support for postpartum depression: a pilot randomized controlled trial. *Journal of Primary Care & Community Health,* **4**(1), 36-43. https://dx.doi.org/10.1177/2150131912451598

Goeppinger, J., Armstrong, B., Schwartz, T., Ensley, D., & Brady, T. J. (2007). Self-management education for persons with arthritis: Managing comorbidity and eliminating health disparities. *Arthritis & Rheumatism-Arthritis Care & Research,* **57**(6), 1081-1088. https://dx.doi.org/10.1002/art.22896

Goggin, K., Gerkovich, M. M., Williams, K. B., Banderas, J. W., Catley, D., Berkley-Patton, J., Wagner, G. J., Stanford, J., Neville, S., Kumar, V. K., Bamberger, D. M., & Clough, L. A. (2013). A randomized controlled trial examining the efficacy of motivational counseling with observed therapy for antiretroviral therapy adherence. *AIDS and Behavior,* **17**(6), 1992-2001. https://dx.doi.org/10.1007/s10461-013-0467-3

Goldberg, R. W., Dickerson, F., Lucksted, A., Brown, C. H., Weber, E., Tenhula, W. N., Kreyenbuhl, J., & Dixon, L. B. (2013). Living Well: An intervention to improve self-management of medical illness for individuals with serious mental illness. *Psychiatric Services,* **64**(1), 51-57. https://dx.doi.org/10.1176/appi.ps.201200034

Gotay, C. C., Moinpour, C. M., Unger, J. M., Jiang, C. S., Coleman, D., Martino, S., Parker, B. J., Bearden, J. D., Dakhil, S., Gross, H. M., Lippman, S., & Albain, K. S. (2007). Impact of a peer-delivered telephone intervention for women experiencing a breast cancer recurrence. *Journal of Clinical Oncology,* **25**(15), 2093-2099. https://dx.doi.org/10.1200/JCO.2006.07.4674

Griswold, K. S., Pastore, P. A., Homish, G. G., & Henke, A. (2010). Access to primary care: Are mental health peers effective in helping patients after a psychiatric emergency? *Primary Psychiatry,* **17**(6), 42-45.

Gross, R., Tierney, C., Andrade, A., Lalama, C., Rosenkranz, S., Eshleman, S. H., Flanigan, T., Santana, J., Salomon, N., Reisler, R., Wiggins, I., Hogg, E., Flexner, C., & Mildvan, D. (2009). Modified directly observed antiretroviral therapy compared with self-administered therapy in treatment-naïve HIV-1-infected patients: A randomized trial. *Archives of Internal Medicine,* **169**(13), 1224-1232. https://dx.doi.org/10.1001/archinternmed.2009.172

Gross, R., Zheng, L., Rosa, A. L., Sun, X., Rosenkranz, S. L., Cardoso, S. W., Ssali, F., Camp, R., Godfrey, C., Cohn, S. E., Robbins, G. K., Chisada, A., Wallis, C. L., Reynolds, N. R., Lu, D., Safren, S. A., Hosey, L., Severe, P., Collier, A. C., & The, A. t. (2015). Partner-based adherence intervention for second-line antiretroviral therapy (ACTG A5234): A multinational randomised trial. *Lancet HIV,* **2**(1), e12-e19. https://dx.doi.org/10.1016/S2352-3018(14)00007-1

Haidari, A., Moeini, M., & Khosravi, A. (2016). Effect of the peer supportive program on blood pressure changes in patients affected with hypertension: A randomized controlled trial. *International Journal of Medical Research & Health Sciences,* **5**(7), 98-102.

Han, Y., Shi, J., Liu, Y., Li, S., Yang, W., & Wang, Y. (2014). Influence of peer education on quality of life of type 2 diabetes patients in Dagang Oilfield. *Med. Soc.,* **27**, 37-39.

Hanks, R. A., Rapport, L. J., Wertheimer, J., & Koviak, C. (2012). Randomized controlled trial of peer mentoring for individuals with traumatic brain injury and their significant others. *Archives of Physical Medicine and Rehabilitation,* **93**(8), 1297-1304. https://dx.doi.org/10.1016/j.apmr.2012.04.027

Harrington, R., Taylor, G., Hollinghurst, S., Reed, M., Kay, H., & Wood, V. A. (2010). A community-based exercise and education scheme for stroke survivors: a randomized controlled trial and economic evaluation. *Clinical Rehabilitation,* **24**(1), 3-15. https://dx.doi.org/10.1177/0269215509347437

Heisler, M., Vijan, S., Makki, F., & Piette, J. D. (2010). Diabetes control with reciprocal peer support versus nurse care management: A randomized trial. *Annals of Internal Medicine,* **153**(8), 507-515. https://dx.doi.org/10.7326/0003-4819-153-8-201010190-00007

Heller, K., Thompson, M. G., Trueba, P. E., Hogg, J. R., & Vlachos-Weber, I. (1991). Peer support telephone dyads for elderly women: was this the wrong intervention? *American Journal of Community Psychology,* **19**(1), 53-74. https://dx.doi.org/10.1007/BF00942253

Herman, S. E., Frank, K. A., Mowbray, C. T., Ribisl, K. M., Davidson, W. S., 2nd, BootsMiller, B., Jordan, L., Greenfield, A. L., Loveland, D., & Luke, D. A. (2000). Longitudinal effects of integrated treatment on alcohol use for persons with serious mental illness and substance use disorders. *Journal of Behavioral Health Services Research,* **27**(3), 286-302. https://dx.doi.org/10.1007/bf02291740

Hill, W., Weinert, C., & Cudney, S. (2006). Influence of a computer intervention on the psychological status of chronically ill rural women: preliminary results. *Nursing Research,* **55**(1), 34-42. https://dx.doi.org/0.1097/00006199-200601000-00005

Holder, H. D., Cisler, R. A., Longabaugh, R., Stout, R. L., Treno, A. J., & Zweben, A. (2000). Alcoholism treatment and medical care costs from Project MATCH. *Addiction,* **95**(7), 999-1013. https://dx.doi.org/10.1046/j.1360-0443.2000.9579993.x

Hopman-Rock, M., & Westhoff, M. H. (2000). The effects of a health educational and exercise program for older adults with osteoarthritis for the hip or knee. *Journal of Rheumatology,* **27**(8), 1947-1954.

Hunkeler, E. M., Meresman, J. F., Hargreaves, W. A., Fireman, B., Berman, W. H., Kirsch, A. J., Groebe, J., Hurt, S. W., Braden, P., Getzell, M., Feigenbaum, P. A., Peng, T., & Salzer, M. (2000). Efficacy of nurse telehealth care and peer support in augmenting treatment of depression in primary care. *Archives of Family Medicine,* **9**(8), 700-708. https://dx.doi.org/10.1001/archfami.9.8.700

Johansson, T., Keller, S., Winkler, H., Ostermann, T., Weitgasser, R., & Sonnichsen, A. C. (2016). Effectiveness of a Peer Support Programme versus Usual Care in Disease Management of Diabetes Mellitus Type 2 regarding Improvement of Metabolic Control: A Cluster-Randomised Controlled Trial. *Journal of Diabetes Research,* **2016**, 3248547. https://dx.doi.org/10.1155/2016/3248547

Johnson, S., Lamb, D., Marston, L., Osborn, D., Mason, O., Henderson, C., Ambler, G., Milton, A., Davidson, M., Christoforou, M., Sullivan, S., Hunter, R., Hindle, D., Paterson, B., Leverton, M., Piotrowski, J., Forsyth, R., Mosse, L., Goater, N., Kelly, K., Lean, M., Pilling, S., Morant, N., & Lloyd-Evans, B. (2018). Peer-supported self-management for people discharged from a mental health crisis team: a randomised controlled trial. *Lancet,* **392**(10145), 409-418. https://dx.doi.org/10.1016/s0140-6736(18)31470-3

Jonikas, J. A., Grey, D. D., Copeland, M. E., Razzano, L. A., Hamilton, M. M., Floyd, C. B., Hudson, W. B., & Cook, J. A. (2013). Improving propensity for patient self-advocacy through Wellness Recovery Action Planning: Results of a randomized controlled trial. *Community Mental Health Journal,* **49**(3), 260-269. https://dx.doi.org/10.1007/s10597-011-9475-9

Ju, C., Shi, R., Yao, L., Ye, X., Jia, M., Han, J., Yang, T., Lu, Q., Jin, H., Cai, X., Yuan, S., Xie, B., Yu, X., Coufal, M. M., Fisher, E. B., & Sun, Z. (2018). Effect of peer support on diabetes distress: a cluster randomized controlled trial. *Diabetic Medicine,* **35**(6), 770-775. https://dx.doi.org/10.1111/dme.13625

Kahler, C. W., Read, J. P., Ramsey, S. E., Stuart, G. L., McCrady, B. S., & Brown, R. A. (2004). Motivational enhancement for 12-step involvement among patients undergoing alcohol detoxification. *Journal of Consulting and Clinical Psychology,* **72**(4), 736-741. https://dx.doi.org/10.1037/0022-006x.72.4.736

Kelly, E., Fulginiti, A., Pahwa, R., Tallen, L., Duan, L., & Brekke, J. S. (2014). A pilot test of a peer navigator intervention for improving the health of individuals with serious mental illness. *Community Mental Health Journal,* **50**(4), 435-446. https://dx.doi.org/10.1007/s10597-013-9616-4

Kelly, J. F., Kaminer, Y., Kahler, C. W., Hoeppner, B., Yeterian, J., Cristello, J. V., & Timko, C. (2017). A pilot randomized clinical trial testing integrated 12-Step facilitation (iTSF) treatment for adolescent substance use disorder. *Addiction,* **112**(12), 2155-2166. https://dx.doi.org/10.1111/add.13920

Kennedy, A., Reeves, D., Bower, P., Lee, V., Middleton, E., Richardson, G., Gardner, C., Gately, C., & Rogers, A. (2007). The effectiveness and cost effectiveness of a national lay-led self care support programme for patients with long-term conditions: a pragmatic randomised controlled trial. *Journal of Epidemiology and Community Health,* **61**(3), 254-261. https://dx.doi.org/10.1136/jech.2006.053538

Keyserling, T. C., Samuel-Hodge, C. D., Ammerman, A. S., Ainsworth, B. E., Henriquez-Roldan, C. F., Elasy, T. A., Skelly, A. H., Johnston, L. F., & Bangdiwala, S. I. (2002). A randomized trial of an intervention to improve self-care behaviors of African-American women with type 2 diabetes - Impact on physical activity. *Diabetes Care,* **25**(9), 1576-1583. https://dx.doi.org/10.2337/diacare.25.9.1576

Kiweewa, F. M., Wabwire, D., Nakibuuka, J., Mubiru, M., Bagenda, D., Musoke, P., Fowler, M. G., & Antelman, G. (2013). Noninferiority of a task-shifting HIV care and treatment model using peer counselors and nurses among Ugandan women initiated on ART: evidence from a randomized trial. *JAIDS-Journal of Acquired Immune Deficiency Syndrome,* **63**(4), e125-132. https://dx.doi.org/10.1097/QAI.0b013e3182987ce6

Klein, A. R., Cnaan, R. A., & Whitecraft, J. (1998). Significance of peer social support with dually diagnosed clients: Findings from a pilot study. *Research on Social Work Practice,* **8**(5), 529-551. https://dx.doi.org/10.1177/104973159800800503

Kovar, P. A., Allegrante, J. P., Mackenzie, C. R., Peterson, M. G. E., Gutin, B., & Charlson, M. E. (1992). Supervised fitness walking in patients with osteoarthritis of the knee - a randomized, controlled trial. *Annals of Internal Medicine,* **116**(7), 529-534. https://dx.doi.org/10.7326/0003-4819-116-7-529

Lee, R., Lee, K. S., Oh, E. G., & Kim, S. H. (2013). A randomized trial of dyadic peer support intervention for newly diagnosed breast cancer patients in Korea. *Cancer Nursing,* **36**(3), E15-E22. https://dx.doi.org/10.1097/NCC.0b013e3182642d7c

Letourneau, N., Stewart, M., Dennis, C. L., Hegadoren, K., Duffett-Leger, L., & Watson, B. (2011). Effect of home-based peer support on maternal-infant interactions among women with postpartum depression: A randomized, controlled trial. *International Journal of Mental Health Nursing,* **20**(5), 345-357. https://dx.doi.org/10.1111/j.1447-0349.2010.00736.x

Litt, M. D., Kadden, R. M., Kabela-Cormier, E., & Petry, N. (2007). Changing network support for drinking: initial findings from the network support project. *Journal of Consulting and Clinical Psychology,* **75**(4), 542-555. https://dx.doi.org/10.1037/0022-006x.75.4.542

Litt, M. D., Kadden, R. M., Kabela-Cormier, E., & Petry, N. M. (2009). Changing network support for drinking: network support project 2-year follow-up. *Journal of Consulting and Clinical Psychology,* **77**(2), 229-242. https://dx.doi.org/10.1037/a0015252

Litt, M. D., Kadden, R. M., Tennen, H., & Kabela-Cormier, E. (2016). Network Support II: Randomized controlled trial of Network Support treatment and cognitive behavioral therapy for alcohol use disorder. *Drug and Alcohol Dependence,* **165**, 203-212. https://dx.doi.org/10.1016/j.drugalcdep.2016.06.010

Liu, Y., Han, Y., Shi, J., Li, R., Li, S., Jin, N., Gu, Y., & Guo, H. (2015). Effect of peer education on self-management and psychological status in type 2 diabetes patients with emotional disorders. *Journal of Diabetes Investigation,* **6**(4), 479-486. https://dx.doi.org/10.1111/jdi.12311

Long, J. A., Jahnle, E. C., Richardson, D. M., Loewenstein, G., & Volpp, K. G. (2012). Peer mentoring and financial incentives to improve glucose control in african american veterans. *Annals of Internal Medicine,* **156**(6), 416-424. https://dx.doi.org/10.7326/0003-4819-156-6-201203200-00004

Longabaugh, R., Wirtz, P. W., Zweben, A., & Stout, R. L. (1998). Network support for drinking, Alcoholics Anonymous and long-term matching effects. *Addiction,* **93**(9), 1313-1333. https://dx.doi.org/10.1046/j.1360-0443.1998.93913133.x

Lorig, K., Gonzalez, V. M., Laurent, D. D., Morgan, L., & Laris, B. A. (1998). Arthritis self-management program variations: Three studies. *Arthritis Care & Research,* **11**(6), 448-454. https://dx.doi.org/10.1002/art.1790110604

Lorig, K., Gonzalez, V. M., & Ritter, P. (1999). Community-based Spanish language arthritis education program - A randomized trial. *Medical Care,* **37**(9), 957-963. https://dx.doi.org/10.1097/00005650-199909000-00011

Lorig, K., Ritter, P. L., Villa, F., & Piette, J. D. (2008). Spanish diabetes self-management with and without automated telephone reinforcement - Two randomized trials. *Diabetes Care,* **31**(3), 408-414. https://dx.doi.org/10.2337/dc07-1313

Lorig, K., Ritter, P. L., Villa, F. J., & Armas, J. (2009). Community-based peer-led diabetes self-management a randomized trial. *Diabetes Educator,* **35**(4), 641-651. https://dx.doi.org/10.1177/0145721709335006

Lorig, K. R., Ritter, P. L., & Gonzalez, V. M. (2003). Hispanic chronic disease self-management - A randomized community-based outcome trial. *Nursing Research,* **52**(6), 361-369. https://dx.doi.org/10.1097/00006199-200311000-00003

Lorig, K. R., Ritter, P. L., & Jacquez, A. (2005). Outcomes of border health Spanish/English chronic disease self-management programs. *Diabetes Educator,* **31**(3), 401-409. https://dx.doi.org/10.1177/0145721705276574

Lorig, K. R., Sobel, D. S., Stewart, A. L., Brown, B. W., Jr., Bandura, A., Ritter, P., Gonzalez, V. M., Laurent, D. D., & Holman, H. R. (1999). Evidence suggesting that a chronic disease self-management program can improve health status while reducing hospitalization: a randomized trial. *Medical Care,* **37**(1), 5-14. https://dx.doi.org/10.1097/00005650-199901000-00003

Low, K. G., Charanasomboon, S., Lesser, J., Reinhalter, K., Martin, R., Jones, H., Winzelberg, A., Abascal, L., & Taylor, C. B. (2006). Effectiveness of a computer-based interactive eating disorders prevention program at long-term follow-up. *Eating Disorders,* **14**(1), 17-30. https://dx.doi.org/10.1080/10640260500403816

Lucas, G. M., Mullen, B. A., Galai, N., Moore, R. D., Cook, K., McCaul, M. E., Glass, S., Oursler, K. K., & Rand, C. (2013). Directly administered antiretroviral therapy for HIV-infected individuals in opioid treatment programs: Results from a randomized clinical trial. *PLoS One,* **8**(7), e68286. https://dx.doi.org/10.1371/journal.pone.0068286

Ludman, E. J., Simon, G. E., Grothaus, L. C., Luce, C., Markley, D. K., & Schaefer, J. (2007). A pilot study of telephone care management and structured disease self-management groups for chronic depression. *Psychiatric Services,* **58**(8), 1065-1072. https://dx.doi.org/10.1176/ps.2007.58.8.1065

Lujan, J., Ostwald, S. K., & Ortiz, M. (2007). Promotora diabetes intervention for Mexican Americans. *Diabetes Educator,* **33**(4), 660-670. https://dx.doi.org/10.1177/0145721707304080

Luo, Q., Xing, G., & Yang, C. (2014). Effect of peer health education on anxiety and depression in patients with type 2 diabetes mellitus. *Journal of Psychiatry,* **27**, 53-55.

Lydecker, K. P., Tate, S. R., Cummins, K. M., McQuaid, J., Granholm, E., & Brown, S. A. (2010). Clinical outcomes of an integrated treatment for depression and substance use disorders. *Psychology of Addictive Behaviors,* **24**(3), 453-465. https://dx.doi.org/10.1037/a0019943

Macalino, G. E., Hogan, J. W., Mitty, J. A., Bazerman, L. B., DeLong, A. K., Loewenthal, H., Caliendo, A. M., & Flanigan, T. P. (2007). A randomized clinical trial of community-based directly observed therapy as an adherence intervention for HAART among substance users. *AIDS,* **21**(11), 1473-1477. https://dx.doi.org/10.1097/QAD.0b013e32811ebf68

Mahlke, C. I., Priebe, S., Heumann, K., Daubmann, A., Wegscheider, K., & Bock, T. (2017). Effectiveness of one-to-one peer support for patients with severe mental illness - a randomised controlled trial. *European Psychiatry,* **42**, 103-110. https://dx.doi.org/10.1016/j.eurpsy.2016.12.007

Manning, V., Best, D., Faulkner, N., Titherington, E., Morinan, A., Keaney, F., Gossop, M., & Strang, J. (2012). Does active referral by a doctor or 12-Step peer improve 12-Step meeting attendance? Results from a pilot randomised control trial. *Drug and Alcohol Dependence,* **126**(1-2), 131-137. https://dx.doi.org/10.1016/j.drugalcdep.2012.05.004

Marmar, C. R., Horowitz, M. J., Weiss, D. S., Wilner, N. R., & Kaltreider, N. B. (1988). A controlled trial of brief psychotherapy and mutual-help group treatment of conjugal bereavement. *American Journal of Psychiatry,* **145**(2), 203-209. https://dx.doi.org/10.1176/ajp.145.2.203

McCrady, B. S., Epstein, E. E., & Hirsch, L. S. (1996). Issues in the implementation of a randomized clinical trial that includes Alcoholics Anonymous: studying AA-related behaviors during treatment. *Journal of Studies on Alcohol,* **57**(6), 604-612. https://dx.doi.org/10.15288/jsa.1996.57.604

McCrady, B. S., Epstein, E. E., & Hirsch, L. S. (1999). Maintaining change after conjoint behavioral alcohol treatment for men: outcomes at 6 months. *Addiction,* **94**(9), 1381-1396. https://dx.doi.org/10.1046/j.1360-0443.1999.949138110.x

McCrady, B. S., Epstein, E. E., & Kahler, C. W. (2004). Alcoholics anonymous and relapse prevention as maintenance strategies after conjoint behavioral alcohol treatment for men: 18-month outcomes. *Journal of Consulting and Clinical Psychology,* **72**(5), 870-878. https://dx.doi.org/10.1037/0022-006x.72.5.870

McGowan, P. (2015). The relative effectiveness of self-management programs for type 2 diabetes. *Canadian Journal of Diabetes,* **39**(5), 411-419. https://dx.doi.org/10.1016/j.jcjd.2015.04.005

McKay, H. G., Glasgow, R. E., Feil, E. G., Boles, S. M., & Barrera, M. (2002). Internet-based diabetes self-management and support: Initial outcomes from the diabetes network project. *Rehabilitation Psychology,* **47**(1), 31-48. https://dx.doi.org/10.1037/0090-5550.47.1.31

Merewood, A., Chamberlain, L. B., Cook, J. T., Philipp, B. L., Malone, K., & Bauchner, H. (2006). The effect of peer counselors on breastfeeding rates in the neonatal intensive care unit: results of a randomized controlled trial. *Archives of Pediatrics & Adolescent Medicine,* **160**(7), 681-685. https://dx.doi.org/10.1001/archpedi.160.7.681

Merlin, J. S., Westfall, A. O., Long, D., Davies, S., Saag, M., Demonte, W., Young, S., Kerns, R. D., Bair, M. J., Kertesz, S., Turan, J. M., Kilgore, M., Clay, O. J., Starrels, J., Pekmezi, D., & Johnson, M. O. (2018). A randomized pilot trial of a novel behavioral intervention for chronic pain tailored to individuals with hiv. *AIDS and Behavior,* **22**(8), 2733-2742. https://dx.doi.org/10.1007/s10461-018-2028-2

Millard, T., Agius, P. A., McDonald, K., Slavin, S., Girdler, S., & Elliott, J. H. (2016). The Positive Outlook Study: A randomised controlled trial evaluating online self-management for HIV positive gay men. *AIDS and Behavior,* **20**(9), 1907-1918. https://dx.doi.org/10.1007/s10461-016-1301-5

Mugusi, F., Mugusi, S., Bakari, M., Hejdemann, B., Josiah, R., Janabi, M., Aboud, S., Aris, E., Swai, H., Mhalu, F., Biberfeld, G., Pallangyo, K., & Sandstrom, E. (2009). Enhancing adherence to antiretroviral therapy at the HIV clinic in resource constrained countries; The Tanzanian experience. *Tropical Medicine and International Health,* **14**(10), 1226-1232. https://dx.doi.org/10.1111/j.1365-3156.2009.02359.x

Murrock, C. J., Higgins, P. A., & Killion, C. (2009). Dance and peer support to improve diabetes outcomes in African American women. *Diabetes Educator,* **35**(6), 995-1003. https://dx.doi.org/10.1177/0145721709343322

Nachega, J. B., Chaisson, R. E., Goliath, R., Efron, A., Chaudhary, M. A., Ram, M., Morroni, C., Schoeman, H., Knowlton, A. R., & Maartens, G. (2010). Randomized controlled trial of trained patient-nominated treatment supporters providing partial directly observed antiretroviral therapy. *AIDS,* **24**(9), 1273-1280. https://dx.doi.org/10.1097/QAD.0b013e328339e20e

Nápoles, A. M., Ortíz, C., Santoyo-Olsson, J., Stewart, A. L., Gregorich, S., Lee, H. E., Durón, Y., McGuire, P., & Luce, J. (2015). Nuevo Amanecer: results of a randomized controlled trial of a community-based, peer-delivered stress management intervention to improve quality of life in Latinas with breast cancer. *American Journal of Public Health,* **105 Suppl 3**(Suppl 3), e55-63. https://dx.doi.org/10.2105/ajph.2015.302598

Niela-Vilén, H., Axelin, A., Melender, H. L., Löyttyniemi, E., & Salanterä, S. (2016). Breastfeeding preterm infants - a randomized controlled trial of the effectiveness of an Internet-based peer-support group. *Journal of Advanced Nursing,* **72**(10), 2495-2507. https://dx.doi.org/10.1111/jan.12993

O’Connell, M. J., Flanagan, E., Delphin, M., & Davidson, L. (2014). Enhancing outcomes for persons with co-occurring disorders through skills training and peer recovery supports. Unpublished manuscript. In.

O'Connell, M. J., Sledge, W. H., Staeheli, M., Sells, D., Costa, M., Wieland, M., & Davidson, L. (2018). Outcomes of a peer mentor intervention for persons with recurrent psychiatric hospitalization. *Psychiatric Services,* **69**(7), 760-767. https://dx.doi.org/10.1176/appi.ps.201600478

Onrust, S., Willemse, G., van den Bout, J., & Cuijpers, P. (2010). Effects of a visiting service for older widowed individuals: a randomized clinical trial. *Death Studies,* **34**(9), 777-803. https://dx.doi.org/10.1080/07481181003761252

Parent, N., & Fortin, F. (2000). A randomized, controlled trial of vicarious experience through peer support for male first-time cardiac surgery patients: impact on anxiety, self-efficacy expectation, and self-reported activity. *Heart & Lung,* **29**(6), 389-400. https://dx.doi.org/10.1067/mhl.2000.110626

Parker, R., Jelsma, J., & Stein, D. J. (2016). Managing pain in women living with HIV/AIDS: A randomized controlled trial testing the effect of a six-week peer-led exercise and education intervention. *Journal of Nervous and Mental Disease,* **204**(9), 665-672. https://dx.doi.org/10.1097/nmd.0000000000000506

Paz-Pacheco, E., Sandoval, M. A., Ardena, G. J., Paterno, E., Juban, N., Lantion-Ang, F. L., Jimeno, C., Patal, P., & Bongon, J. (2017). Effectiveness of a community-based diabetes self-management education (DSME) program in a rural agricultural setting. *Primary Health Care Research & Development,* **18**(1), 35-49. https://dx.doi.org/10.1017/S1463423616000335

Pearson, C. R., Micek, M. A., Simoni, J. M., Hoff, P. D., Matediana, E., Martin, D. P., & Gloyd, S. S. (2007). Randomized control trial of peer-delivered, modified directly observed therapy for HAART in Mozambique. *Journal of Acquired Immune Deficiency Syndromes,* **46**(2), 238-244. https://dx.doi.org/10.1097/QAI.0b013e318153f7ba

Peimani, M., Monjazebi, F., Ghodssi-Ghassemabadi, R., & Nasli-Esfahani, E. (2018). A peer support intervention in improving glycemic control in patients with type 2 diabetes. *Patient Education and Counseling,* **101**(3), 460-466. https://dx.doi.org/10.1016/j.pec.2017.10.007

Pérez-Escamilla, R., Damio, G., Chhabra, J., Fernandez, M. L., Segura-Pérez, S., Vega-López, S., Kollannor-Samuel, G., Calle, M., Shebl, F. M., & D'Agostino, D. (2015). Impact of a community health workers-led structured program on blood glucose control among Latinos with type 2 diabetes: The DIALBEST Trial. *Diabetes Care,* **38**(2), 197-205. https://dx.doi.org/10.2337/dc14-0327

Philis-Tsimikas, A., Fortmann, A., Lleva-Ocana, L., Walker, C., & Gallo, L. C. (2011). Peer-led diabetes education programs in high-risk Mexican Americans improve glycemic control compared with standard approaches: a Project Dulce promotora randomized trial. *Diabetes Care,* **34**(9), 1926-1931. https://dx.doi.org/10.2337/dc10-2081

Pickett, S. A., Diehl, S. M., Steigman, P. J., Prater, J. D., Fox, A., Shipley, P., Grey, D. D., & Cook, J. A. (2012). Consumer empowerment and self-advocacy outcomes in a randomized study of peer-led education. *Community Mental Health Journal,* **48**(4), 420-430. https://dx.doi.org/10.1007/s10597-012-9507-0

Pinto, B., Stein, K., & Dunsiger, S. (2015). Peer mentorship to promote physical activity among cancer survivors: effects on quality of life. *Psychooncology,* **24**(10), 1295-1302. https://dx.doi.org/10.1002/pon.3884

Pinto, B. M., Stein, K., & Dunsiger, S. (2015). Peers promoting physical activity among breast cancer survivors: A randomized controlled trial. *Health Psychology,* **34**(5), 463-472. https://dx.doi.org/10.1037/hea0000120

Porter, L. S., Keefe, F. J., Baucom, D. H., Hurwitz, H., Moser, B., Patterson, E., & Kim, H. J. (2009). Partner-assisted emotional disclosure for patients with gastrointestinal cancer: results from a randomized controlled trial. *Cancer,* **115**(18 Suppl), 4326-4338. https://dx.doi.org/10.1002/cncr.24578

Prezio, E. A., Cheng, D., Balasubramanian, B. A., Shuval, K., Kendzor, D. E., & Culica, D. (2013). Community Diabetes Education (CoDE) for uninsured Mexican Americans: a randomized controlled trial of a culturally tailored diabetes education and management program led by a community health worker. *Diabetes Research and Clinical Practice,* **100**(1), 19-28. https://dx.doi.org/10.1016/j.diabres.2013.01.027

Project MATCH. (1997). Matching alcoholism treatments to client heterogeneity: project MATCH post-treatment drinking outcomes. *Journal of Studies on Alcohol,* **58**, 7‐29.

Project MATCH. (1998). Matching alcoholism treatments to client heterogeneity: project MATCH three-year drinking outcomes. *Alcoholism, Clinical and Experimental research,* **22**(6), 1300‐1311. https://dx.doi.org/10.1111/j.1530-0277.1998.tb03912.x

Project MATCH. (1998). Matching alcoholism treatments to client heterogeneity: treatment main effects and matching effects on drinking during treatment. Project MATCH Research Group. *Journal of Studies on Alcohol,* **59**(6), 631‐639. https://dx.doi.org/10.15288/jsa.1998.59.631

Protheroe, J., Rathod, T., Bartlam, B., Rowlands, G., Richardson, G., & Reeves, D. (2016). The feasibility of health trainer improved patient self-management in patients with low health literacy and poorly controlled diabetes: A pilot randomised controlled trial. *Journal of Diabetes Research,* **2016**, 6903245. https://dx.doi.org/10.1155/2016/6903245

Proudfoot, J., Parker, G., Manicavasagar, V., Hadzi-Pavlovic, D., Whitton, A., Nicholas, J., Smith, M., & Burckhardt, R. (2012). Effects of adjunctive peer support on perceptions of illness control and understanding in an online psychoeducation program for bipolar disorder: a randomised controlled trial. *Journal of Affective Disorders,* **142**(1-3), 98-105. https://dx.doi.org/10.1016/j.jad.2012.04.007

Purcell, D. W., Latka, M. H., Metsch, L. R., Latkin, C. A., Gomez, C. A., Mizuno, Y., Arnsten, J. H., Wilkinson, J. D., Knight, K. R., Knowlton, A. R., Santibanez, S., Tobin, K. E., Rose, C. D., Valverde, E. E., Gourevitch, M. N., Eldred, L., Borkowf, C. B., & Team, I. S. (2007). Results from a randomized controlled trial of a peer-mentoring intervention to reduce HIV transmission and increase access to care and adherence to HIV medications among HIV-seropositive injection drug users. *JAIDS-Journal of Acquired Immune Deficiency Syndrome,* **46**, S35-S47. https://dx.doi.org/10.1097/QAI.0b013e31815767c4

Qian, Y. (2015). Peer support for the quality of life in patients with schizophrenia. *Health for Everyone,* **23**, 7.

Rashidi, K., Safavi, M., Yahyavi, H., & Farahani, H. (2017). The impact of peers’ support on the hemoglobin A1C and fasting blood sugar level of patients with type 2 diabetes. *Indian Journal of Medical Specialities,* **8**(1), 7-12. https://dx.doi.org/10.1016/j.injms.2016.10.001

Remien, R. H., Stirratt, M. J., Dolezal, C., Dognin, J. S., Wagner, G. J., Carballo-Dieguez, A., El-Bassel, N., & Jung, T. M. (2005). Couple-focused support to improve HIV medication adherence: A randomized controlled trial. *AIDS,* **19**(8), 807-814. https://dx.doi.org/10.1097/01.aids.0000168975.44219.45

Reynolds, W., Lauder, W., Sharkey, S., Maciver, S., Veitch, T., & Cameron, D. (2004). The effects of a transitional discharge model for psychiatric patients. *Journal of Psychiatric and Mental Health Nursing,* **11**(1), 82-88. https://dx.doi.org/10.1111/j.1365-2850.2004.00692.x

Richter, L., Rotheram-Borus, M. J., Van Heerden, A., Stein, A., Tomlinson, M., Harwood, J. M., Rochat, T., Van Rooyen, H., Comulada, W. S., & Tang, Z. (2014). Pregnant women living with HIV (WLH) supported at clinics by peer WLH: a cluster randomized controlled trial. *AIDS and Behavior,* **18**(4), 706-715. https://dx.doi.org/10.1007/s10461-014-0694-2

Riddell, M. A., Dunbar, J. A., Absetz, P., Wolfe, R., Li, H., Brand, M., Aziz, Z., Oldenburg, B., & Australasian Peers for Progress Diabetes Project, I. (2016). Cardiovascular risk outcome and program evaluation of a cluster randomised controlled trial of a community-based, lay peer led program for people with diabetes. *BMC Public Health,* **16**(1), 864. https://dx.doi.org/10.1186/s12889-016-3538-3

Rivera, J. J., Sullivan, A. M., & Valenti, S. S. (2007). Adding consumer-providers to intensive case management: does it improve outcome? *Psychiatric Services,* **58**(6), 802-809. https://dx.doi.org/10.1176/ps.2007.58.6.802

Robinson-Whelen, S., Hughes, R. B., Taylor, H. B., Colvard, M., Mastel-Smith, B., & Nosek, M. A. (2006). Improving the health and health behaviors of women aging with physical disabilities: A peer-led health promotion program. *Womens Health Issues,* **16**(6), 334-345. https://dx.doi.org/10.1016/j.whi.2006.05.002

Robinson-Whelen, S., Hughes, R. B., Taylor, H. B., Hall, J. W., & Rehm, L. P. (2007). Depression self-management physical program for rural women with disabilities. *Rehabilitation Psychology,* **52**(3), 254-262. https://dx.doi.org/10.1037/0090-5550.52.3.254

Rogers, E. S., Maru, M., Johnson, G., Cohee, J., Hinkel, J., & Hashemi, L. (2016). A randomized trial of individual peer support for adults with psychiatric disabilities undergoing civil commitment. *Psychiatric Rehabilitation Journal,* **39**(3), 248-255. https://dx.doi.org/10.1037/prj0000208

Rogers, E. S., Teague, G. B., Lichenstein, C., Campbell, J., Lyass, A., Chen, R., & Banks, S. (2007). Effects of participation in consumer-operated service programs on both personal and organizationally mediated empowerment: Results of multisite study. *Journal of Rehabilitation Research and Development,* **44**(6), 785-799. https://dx.doi.org/10.1682/jrrd.2006.10.0125

Rosal, M. C., Ockene, I. S., Restrepo, A., White, M. J., Borg, A., Olendzki, B., Scavron, J., Candib, L., Welch, G., & Reed, G. (2011). Randomized trial of a literacy-sensitive, culturally tailored diabetes self-management intervention for low-income latinos latinos en control. *Diabetes Care,* **34**(4), 838-844. https://dx.doi.org/10.2337/dc10-1981

Rotheram-Borus, M. J., Richter, L. M., van Heerden, A., van Rooyen, H., Tomlinson, M., Harwood, J. M., Comulada, W. S., & Stein, A. (2014). A cluster randomized controlled trial evaluating the efficacy of peer mentors to support South African women living with HIV and their infants. *PLoS One,* **9**(1), e84867-e84867. https://dx.doi.org/10.1371/journal.pone.0084867

Rothschild, S. K., Martin, M. A., Swider, S. M., Lynas, C. M. T., Janssen, I., Avery, E. F., & Powell, L. H. (2014). Mexican american trial of community health workers: A randomized controlled trial of a community health worker intervention for mexican americans with type 2 diabetes mellitus. *American Journal of Public Health,* **104**(8), 1540-1548. https://dx.doi.org/10.2105/AJPH.2013.301439

Rowe, M., Bellamy, C., Baranoski, M., Wieland, M., Connell, M. J. O., Benedict, P., Davidson, L., Buchanan, J., & Sells, D. (2007). A peer-support, group intervention to reduce substance use and criminality among persons with severe mental illness. *Psychiatric Services,* **58**(7), 955-961. https://dx.doi.org/10.1176/appi.ps.58.7.955

Ruggiero, L., Moadsiri, A., Butler, P., Oros, S. M., Berbaum, M. L., Whitman, S., & Cintron, D. (2010). Supporting diabetes self-care in underserved populations: A randomized pilot study using medical assistant coaches. *Diabetes Educator,* **36**(1), 127-131. https://dx.doi.org/10.1177/0145721709355487

Ruiz, I., Olry, A., Lopez, M. A., Prada, J. L., & Causse, M. (2010). Prospective, randomized, two-arm controlled study to evaluate two interventions to improve adherence to antiretroviral therapy in Spain. *Enfermedades Infecciosas Y Microbiologia Clinica,* **28**(7), 409-415. https://dx.doi.org/10.1016/j.eimc.2009.03.018

Rüsch, N., Abbruzzese, E., Hagedorn, E., Hartenhauer, D., Kaufmann, I., Curschellas, J., Ventling, S., Zuaboni, G., Bridler, R., Olschewski, M., Kawohl, W., Rössler, W., Kleim, B., & Corrigan, P. W. (2014). Efficacy of Coming Out Proud to reduce stigma's impact among people with mental illness: pilot randomised controlled trial. *British Journal of Psychiatry,* **204**(5), 391-397. https://dx.doi.org/10.1192/bjp.bp.113.135772

Russinova, Z., Rogers, E. S., Gagne, C., Bloch, P., Drake, K. M., & Mueser, K. T. (2014). A randomized controlled trial of a peer-run antistigma photovoice intervention. *Psychiatric Services,* **65**(2), 242-246. https://dx.doi.org/10.1176/appi.ps.201200572

Safford, M. M., Andreae, S., Cherrington, A. L., Martin, M. Y., Halanych, J., Lewis, M., Patel, A., Johnson, E., Clark, D., Gamboa, C., & Richman, J. S. (2015). Peer coaches to improve diabetes outcomes in rural Alabama: A cluster randomized trial. *Annals of Family Medicine,* **13**, S18-S26. https://dx.doi.org/10.1370/afm.1798

Salzer, M. S., Rogers, J., Salandra, N., O'Callaghan, C., Fulton, F., Balletta, A. A., Pizziketti, K., & Brusilovskiy, E. (2016). Effectiveness of peer-delivered Center for Independent Living supports for individuals with psychiatric disabilities: A randomized, controlled trial. *Psychiatric Rehabilitation Journal,* **39**(3), 239-247. https://dx.doi.org/10.1037/prj0000220

Samarel, N., Fawcett, J., & Tulman, L. (1997). Effect of support groups with coaching on adaptation to early stage breast cancer. *Research in Nursing & Health,* **20**(1), 15-26. https://dx.doi.org/10.1002/(sici)1098-240x(199702)20:1<15::aid-nur3>3.0.co;2-x

Samuel-Hodge, C. D., Keyserling, T. C., Park, S., Johnston, L. F., Gizlice, Z., & Bangdiwala, S. I. (2009). A randomized trial of a church-based diabetes self-management program for African Americans with type 2 diabetes. *Diabetes Educator,* **35**(3), 439-454. https://dx.doi.org/10.1177/0145721709333270

Sazlina, S. G., Browning, C. J., & Yasin, S. (2015). Effectiveness of personalized feedback alone or combined with peer support to improve physical activity in sedentary older Malays with type 2 diabetes: a randomized controlled trial. *Frontiers in Public Health,* **3**, 11. https://dx.doi.org/10.3389/fpubh.2015.00178

Schover, L. R., Jenkins, R., Sui, D., Adams, J. H., Marion, M. S., & Jackson, K. E. (2006). Randomized trial of peer counseling on reproductive health in African American breast cancer survivors. *Journal of Clinical Oncology,* **24**(10), 1620-1626. https://dx.doi.org/10.1200/jco.2005.04.7159

Schover, L. R., Rhodes, M. M., Baum, G., Adams, J. H., Jenkins, R., Lewis, P., & Jackson, K. E. (2011). Sisters Peer Counseling in Reproductive Issues after Treatment (SPIRIT): A peer counseling program to improve reproductive health among African American breast cancer survivors. *Cancer,* **117**(21), 4983-4992. https://dx.doi.org/10.1002/cncr.26139

Seeley, J. R., Manitsas, T., & Gau, J. M. (2017). Feasibility study of a peer-facilitated low intensity cognitive-behavioral intervention for mild to moderate depression and anxiety in older adults. *Aging & Mental Health,* **21**(9), 968-974. https://dx.doi.org/10.1080/13607863.2016.1186152

Segal, S. P., Silverman, C. J., & Temkin, T. L. (2010). Self-help and community mental health agency outcomes: A recovery-focused randomized controlled trial. *Psychiatric Services,* **61**(9), 905-910. https://dx.doi.org/10.1176/ps.2010.61.9.905

Segal, S. P., Silverman, C. J., & Temkin, T. L. (2011). Outcomes from consumer-operated and community mental health services: A randomized controlled trial. *Psychiatric Services,* **62**(8), 915-921. https://dx.doi.org/10.1176/ps.62.8.pss6208_0915

Selke, H. M., Kimaiyo, S., Sidle, J. E., Vedanthan, R., Tierney, W. M., Shen, C., Denski, C. D., Katschke, A. R., & Wools-Kaloustian, K. (2010). Task-shifting of antiretroviral delivery from health care workers to persons living with HIV/AIDS: clinical outcomes of a community-based program in Kenya. *JAIDS-Journal of Acquired Immune Deficiency Syndrome,* **55**(4), 483-490. https://dx.doi.org/10.1097/QAI.0b013e3181eb5edb

Sells, D., Black, R., Davidson, L., & Rowe, M. (2008). Beyond generic support: incidence and impact of invalidation in peer services for clients with severe mental illness. *Psychiatric Services,* **59**(11), 1322-1327. https://dx.doi.org/10.1176/ps.2008.59.11.1322

Sells, D., Davidson, L., Jewell, C., Falzer, P., & Rowe, M. (2006). The treatment relationship in peer-based and regular case management for clients with severe mental illness. *Psychiatric Services,* **57**(8), 1179-1184. https://dx.doi.org/10.1176/ps.2006.57.8.1179

Shaya, F. T., Chirikov, V. V., Howard, D., Foster, C., Costas, J., Snitker, S., Frimpter, J., & Kucharski, K. (2014). Effect of social networks intervention in type 2 diabetes: a partial randomised study. *Journal of Epidemiology Community Health,* **68**(4), 326-332. https://dx.doi.org/10.1136/jech-2013-203274

Si, L., Duan, P., & Liu, J. (2016). Influence of peer support education with WeChat group on blood glucose and self-management ability of patients with diabetes mellitus. *Chinese Nursing Research,* **30**(9), 3382-3384.

Siminerio, L., Ruppert, K. M., & Gabbay, R. A. (2013). Who can provide diabetes self-management support in primary care? Findings from a randomized controlled trial. *Diabetes Educator,* **39**(5), 705-713. https://dx.doi.org/10.1177/0145721713492570

Simmons, D., Prevost, A. T., Bunn, C., Holman, D., Parker, R. A., Cohn, S., Donald, S., Paddison, C. A., Ward, C., Robins, P., & Graffy, J. (2015). Impact of community based peer support in type 2 diabetes: a cluster randomised controlled trial of individual and/or group approaches. *PLoS One,* **10**(3), e0120277. https://dx.doi.org/10.1371/journal.pone.0120277

Simon, G. E., Ludman, E. J., Goodale, L. C., Dykstra, D. M., Stone, E., Cutsogeorge, D., Operskalski, B., Savarino, J., & Pabiniak, C. (2011). An online recovery plan program: can peer coaching increase participation? *Psychiatric Services,* **62**(6), 666-669. https://dx.doi.org/10.1176/ps.62.6.pss6206_0666

Simoni, J. M., Huh, D., Frick, P. A., Pearson, C. R., Andrasik, M. P., Dunbar, P. J., & Hooton, T. M. (2009). Peer support and pager messaging to promote antiretroviral modifying therapy in seattle: A randomized controlled trial. *JAIDS-Journal of Acquired Immune Deficiency Syndrome,* **52**(4), 465-473. https://dx.doi.org/10.1097/QAI.0b013e3181b9300c

Simoni, J. M., Pantalone, D. W., Plummer, M. D., & Huang, B. (2007). A randomized controlled trial of a peer support intervention targeting antiretroviral medication adherence and depressive symptomatology in HIV-positive men and women. *Health Psychology,* **26**(4), 488-495. https://dx.doi.org/10.1037/0278-6133.26.4.488

Simpson, A., Flood, C., Rowe, J., Quigley, J., Henry, S., Hall, C., Evans, R., Sherman, P., & Bowers, L. (2014). Results of a pilot randomised controlled trial to measure the clinical and cost effectiveness of peer support in increasing hope and quality of life in mental health patients discharged from hospital in the UK. *BMC Psychiatry,* **14**, 30. https://dx.doi.org/10.1186/1471-244x-14-30

Sledge, W. H., Lawless, M., Sells, D., Wieland, M., O'Connell, M. J., & Davidson, L. (2011). Effectiveness of peer support in reducing readmissions of persons with multiple psychiatric hospitalizations. *Psychiatric Services,* **62**(5), 541-544. https://dx.doi.org/10.1176/ps.62.5.pss6205_0541

Smith, S. M., Paul, G., Kelly, A., Whitford, D. L., O'Shea, E., & O'Dowd, T. (2011). Peer support for patients with type 2 diabetes: Cluster randomised controlled trial. *BMJ,* **342**(7795), 482. https://dx.doi.org/10.1136/bmj.d715

Solomon, P., & Draine, J. (1995). The efficacy of a consumer case management team: 2-year outcomes of a randomized trial. *Journal of Mental Health Administration,* **22**(2), 135-146. https://dx.doi.org/10.1007/bf02518754

Solomon, P., & Draine, J. (1995). One-year outcomes of a randomized trial of consumer case management. *Evaluation and Program Planning,* **18**(2), 117-127. https://doi.org/10.1016/0149-7189(95)00003-T

Solomon, P., & Draine, J. (1996). Service delivery differences between consumer and nonconsumer case managers in mental health. *Research on Social Work Practice,* **6**(2), 193-207. https://dx.doi.org/10.1177/104973159600600204

Solomon, P., Draine, J., & Delaney, M. A. (1995). The working alliance and consumer case management. *Journal of Mental Health Administration,* **22**(2), 126-134. https://dx.doi.org/10.1007/bf02518753

Spencer, M. S., Rosland, A.-M., Kieffer, E. C., Sinco, B. R., Valerio, M., Palmisano, G., Anderson, M., Guzman, J. R., & Heisler, M. (2011). Effectiveness of a community health worker intervention among African American and Latino adults with type 2 diabetes: A randomized controlled trial. *American Journal of Public Health,* **101**(12), 2253-2260. https://dx.doi.org/10.2105/ajph.2010.300106

Sreedevi, A., Unnikrishnan, A. G., Karimassery, S. R., & Deepak, K. S. (2017). The effect of yoga and peer support interventions on the quality of life of women with diabetes: Results of a randomized controlled trial. *Indian Journal of Endocrinology and Metabolism,* **21**(4), 524-530. https://dx.doi.org/10.4103/ijem.IJEM_28_17

Stamatakis, C. (2015). *The efficacy of peer support in stroke rehabilitation.* (DClinPsy). Cardiff University, Cardiff.

Stant, A. D., Castelein, S., Bruggeman, R., van Busschbach, J. T., van der Gaag, M., Knegtering, H., & Wiersma, D. (2011). Economic aspects of peer support groups for psychosis. *Community Mental Health Journal,* **47**(1), 99-105. https://dx.doi.org/10.1007/s10597-009-9193-8

Struchen, M. A., Davis, L. C., Bogaards, J. A., Hudler-Hull, T., Clark, A. N., Mazzei, D. M., Sander, A. M., & Caroselli, J. S. (2011). Making connections after brain injury: development and evaluation of a social peer-mentoring program for persons with traumatic brain injury. *Journal of Head Trauma Rehabilitation,* **26**(1), 4-19. https://dx.doi.org/10.1097/HTR.0b013e3182048e98

Sullivan, T., Allegrante, J. P., Peterson, M. G. E., Kovar, P. A., & MacKenzie, C. R. (1998). One-year followup of patients with osteoarthritis of the knee who participated in a program of supervised fitness walking and supportive patient education. *Arthritis Care & Research,* **11**(4), 228-233. https://dx.doi.org/10.1002/art.1790110403

Swerissen, H., Belfrage, J., Weeks, A., Jordan, L., Walker, C., Furler, J., McAvoy, B., Carter, M., & Peterson, C. (2006). A randomised control trial of a self-management program for people with a chronic illness from Vietnamese, Chinese, Italian and Greek backgrounds. *Patient Education and Counseling,* **64**(1-3), 360-368. https://dx.doi.org/10.1016/j.pec.2006.04.003

Taiwo, B. O., Idoko, J. A., Welty, L. J., Otoh, I., Job, G., Iyaji, P. G., Agbaji, O., Agaba, P. A., & Murphy, R. L. (2010). Assessing the viorologic and adherence benefits of patient-selected HIV treatment partners in a resource-limited setting. *Journal of Acquired Immune Deficiency Syndromes,* **54**(1), 85-92. https://dx.doi.org/10.1097/01.qai.0000371678.25873.1c

Tang, T. S., Funnell, M., Sinco, B., Piatt, G., Palmisano, G., Spencer, M. S., Kieffer, E. C., & Heisler, M. (2014). Comparative effectiveness of peer leaders and community health workers in diabetes selfmanagement support: Results of a randomized controlled trial. *Diabetes Care,* **37**(6), 1525-1534. https://dx.doi.org/10.2337/dc13-2161

Tang, T. S., Funnell, M. M., Sinco, B., Spencer, M. S., & Heisler, M. (2015). Peer-Led, Empowerment-Based Approach to Self-Management Efforts in Diabetes (PLEASED): A randomized controlled trial in an African American community. *Annals of Family Medicine,* **13**(Suppl 1), S27-35. https://dx.doi.org/10.1370/afm.1819

Thom, D. H., Ghorob, A., Hessler, D., De Vore, D., Chen, E., & Bodenheimer, T. A. (2013). Impact of peer health coaching on glycemic control in low-income patients with diabetes: a randomized controlled trial. *Annals of Family Medicine,* **11**(2), 137-144. https://dx.doi.org/10.1370/afm.1443

Tian, H. (2013). Study on the influence of peer education on adverse mood of high-risk pregnant. *Chinese Journal of Integrated Traditional and Western Medicine,* **29**, 3287-3288.

Timko, C., & DeBenedetti, A. (2007). A randomized controlled trial of intensive referral to 12-step self-help groups: One-year outcomes. *Drug and Alcohol Dependence,* **90**(2), 270-279. https://doi.org/10.1016/j.drugalcdep.2007.04.007

Timko, C., Debenedetti, A., & Billow, R. (2006). Intensive referral to 12-Step self-help groups and 6-month substance use disorder outcomes. *Addiction,* **101**(5), 678-688. https://dx.doi.org/10.1111/j.1360-0443.2006.01391.x

Tracy, K., Burton, M., Nich, C., & Rounsaville, B. (2011). Utilizing peer mentorship to engage high recidivism substance-abusing patients in treatment. *American Journal of Drug and Alcohol Abuse,* **37**(6), 525-531. https://dx.doi.org/10.3109/00952990.2011.600385

Travis, J., Roeder, K., Walters, H., Piette, J., Heisler, M., Ganoczy, D., Valenstein, M., & Pfeiffer, P. (2010). Telephone-based mutual peer support for depression: a pilot study. *Chronic Illness,* **6**(3), 183-191. https://dx.doi.org/10.1177/1742395310369570

Turner, B. J., Hollenbeak, C. S., Liang, Y., Pandit, K., Joseph, S., & Weiner, M. G. (2012). A randomized trial of peer coach and office staff support to reduce coronary heart disease risk in African-Americans with uncontrolled hypertension. *Journal of General Internal Medicine,* **27**(10), 1258-1264. https://dx.doi.org/10.1007/s11606-012-2095-4

Two Feathers, J., Kieffer, E. C., Palmisano, G., Anderson, M., Sinco, B., Janz, N., Heisler, M., Spencer, M., Guzman, R., Thompson, J., Wisdom, K., & James, S. A. (2005). Racial and Ethnic Approaches to Community Health (REACH) Detroit partnership: improving diabetes-related outcomes among African American and Latino adults. *American Journal of Public Health,* **95**(9), 1552-1560. https://dx.doi.org/10.2105/ajph.2005.066134

van der Wulp, I., de Leeuw, J. R. J., Gorter, K. J., & Rutten, G. (2012). Effectiveness of peer-led self-management coaching for patients recently diagnosed with Type 2 diabetes mellitus in primary care: a randomized controlled trial. *Diabetic Medicine,* **29**(10), e390-e397. https://dx.doi.org/10.1111/j.1464-5491.2012.03629.x

van Gestel-Timmermans, H., Brouwers, E. P., van Assen, M. A., & van Nieuwenhuizen, C. (2012). Effects of a peer-run course on recovery from serious mental illness: a randomized controlled trial. *Psychiatric Services,* **63**(1), 54-60. https://dx.doi.org/10.1176/appi.ps.201000450

van Gestel-Timmermans, J., Brouwers, E. P. M., & van Nieuwenhuizen, C. (2010). Recovery Is Up to You, a peer-run course. *Psychiatric Services,* **61**(9), 944-945. https://dx.doi.org/10.1176/ps.2010.61.9.944a

Vederhus, J. K., Timko, C., Kristensen, O., Hjemdahl, B., & Clausen, T. (2014). Motivational intervention to enhance post-detoxification 12-Step group affiliation: a randomized controlled trial. *Addiction,* **109**(5), 766-773. https://dx.doi.org/10.1111/add.12471

Vincent, D., Pasvogel, A., & Barrera, L. (2007). A feasibility study of a culturally tailored diabetes intervention for Mexican Americans. *Biological Research for Nursing,* **9**(2), 130-141. https://dx.doi.org/10.1177/1099800407304980

Walitzer, K. S., Deffenbacher, J. L., & Shyhalla, K. (2015). Alcohol-adapted anger management treatment: A randomized controlled trial of an innovative therapy for alcohol dependence. *Journal of Substance Abuse Treatment,* **59**, 83-93. https://dx.doi.org/10.1016/j.jsat.2015.08.003

Walitzer, K. S., Dermen, K. H., & Barrick, C. (2009). Facilitating involvement in Alcoholics Anonymous during out-patient treatment: a randomized clinical trial. *Addiction,* **104**(3), 391-401. https://dx.doi.org/10.1111/j.1360-0443.2008.02467.x

Wang, H., Zhou, J., Huang, L., Li, X., Fennie, K. P., & Williams, A. B. (2010). Effects of nurse-delivered home visits combined with telephone calls on medication adherence and quality of life in HIV-infected heroin users in Hunan of China. *Journal of Clinical Nursing,* **19**(3-4), 380-388. https://dx.doi.org/10.1111/j.1365-2702.2009.03048.x

Webel, A. R. (2010). Testing a peer-based symptom management intervention for women living with HIV/AIDS. *Aids Care-Psychological and Socio-Medical Aspects of Aids/Hiv,* **22**(9), 1029-1040. https://dx.doi.org/10.1080/09540120903214389

Weber, B. A., Roberts, B. L., Resnick, M., Deimling, G., Zauszniewski, J. A., Musil, C., & Yarandi, H. N. (2004). The effect of dyadic intervention on self-efficacy, social support, and depression for men with prostate cancer. *Psychooncology,* **13**(1), 47-60. https://dx.doi.org/10.1002/pon.718

Weber, B. A., Roberts, B. L., Yarandi, H., Mills, T. L., Chumbler, N. R., & Algood, C. (2007). Dyadic support and quality-of-life after radical prostatectomy. *Journal of Men's Health and Gender,* **4**(2), 156-164. https://dx.doi.org/10.1016/j.jmhg.2007.03.004

Weber, B. A., Roberts, B. L., Yarandi, H., Mills, T. L., Chumbler, N. R., & Wajsman, Z. (2007). The impact of dyadic social support on self-efficacy and depression after radical prostatectomy. *Journal of Aging and Health,* **19**(4), 630-645. https://dx.doi.org/10.1177/0898264307300979

Wei, H., & Han, Y. (2017). Study on the influence of peer education on the biochemical index, self-management ability and psychological status of type 2 diabetes patients. *China Medical Herald,* **14**, 62-65.

Whittle, J., Schapira, M. M., Fletcher, K. E., Hayes, A., Morzinski, J., Laud, P., Eastwood, D., Ertl, K., Patterson, L., & Mosack, K. E. (2014). A randomized trial of peer-delivered self-management support for hypertension. *American Journal of Hypertension,* **27**(11), 1416-1423. https://dx.doi.org/10.1093/ajh/hpu058

Williams, A. B., Fennie, K. P., Bova, C. A., Burgess, J. D., Danvers, K. A., & Dieckhaus, K. D. (2006). Home visits to improve adherence to highly active antiretroviral therapy: A randomized controlled trial. *Journal of Acquired Immune Deficiency Syndromes,* **42**(3), 314-321. https://dx.doi.org/10.1097/01.qai.0000221681.60187.88

Williams, A. B., Wang, H., Li, X., Chen, J., Li, L., & Fennie, K. (2014). Efficacy of an evidence-based ARV adherence intervention in China. *AIDS Patient Care and STDs,* **28**(8), 411-417. https://dx.doi.org/10.1089/apc.2014.0070

Williams, A. M., Bloomfield, L., Milthorpe, E., Aspinall, D., Filocamo, K., Wellsmore, T., Manolios, N., Jayasinghe, U. W., & Harris, M. F. (2013). Effectiveness of moving on: an Australian designed generic self-management program for people with a chronic illness. *BMC Health Services Research,* **13**. https://dx.doi.org/10.1186/1472-6963-13-90

Wohl, A. R., Garland, W. H., Valencia, R., Squires, K., Witt, M. D., Kovacs, A., Larsen, R., Hader, S., Anthony, M. N., & Weidle, P. J. (2006). A randomized trial of directly administered antiretroviral therapy and adherence case management intervention. *Clinical Infectious Diseases,* **42**(11), 1619-1627. https://dx.doi.org/10.1086/503906

Wolitski, R. J., Gomez, C. A., Parsons, J. T., & Grp, S. S. (2005). Effects of a peer-led behavioral intervention to reduce HIV transmission and promote serostatus disclosure among HIV-seropositive gay and bisexual men. *AIDS,* **19**, S99-S109. https://dx.doi.org/10.1097/01.aids.0000167356.94664.59

Woodruff, S. I., Conway, T. L., Edwards, C. C., Elliott, S. P., & Crittenden, J. (2007). Evaluation of an Internet virtual world chat room for adolescent smoking cessation. *Addictive Behaviors,* **32**(9), 1769-1786. https://dx.doi.org/10.1016/j.addbeh.2006.12.008

Wrobleski, T., Walker, G., Jarus-Hakak, A., & Suto, M. J. (2015). Peer support as a catalyst for recovery: a mixed-methods study. *Canadian Journal of Occupational Therapy,* **82**(1), 64-73. https://dx.doi.org/10.1177/0008417414551784

Wu, C. J., Chang, A. M., Courtney, M., & Kostner, K. (2012). Peer supporters for cardiac patients with diabetes: a randomized controlled trial. *International Nursing Review,* **59**(3), 345-352. https://dx.doi.org/10.1111/j.1466-7657.2012.00998.x

Wu, S. F. V., Lee, M. C., Liang, S. Y., Lu, Y. Y., Wang, T. J., & Tung, H. H. (2011). Effectiveness of a self-efficacy program for persons with diabetes: A randomized controlled trial. *Nursing and Health Sciences,* **13**(3), 335-343. https://dx.doi.org/10.1111/j.1442-2018.2011.00625.x

Yamaguchi, S., Taneda, A., Matsunaga, A., Sasaki, N., Mizuno, M., Sawada, Y., Sakata, M., Fukui, S., Hisanaga, F., Bernick, P., & Ito, J. (2017). Efficacy of a peer-led, recovery-oriented shared decision-making system: A pilot randomized controlled trial. *Psychiatric Services,* **68**(12), 1307-1311. https://dx.doi.org/10.1176/appi.ps.201600544

Yun, Y. H., Kim, Y. A., Lee, M. K., Sim, J. A., Nam, B. H., Kim, S., Lee, E. S., Noh, D. Y., Lim, J. Y., Kim, S., Kim, S. Y., Cho, C. H., Jung, K. H., Chun, M., Lee, S. N., Park, K. H., & Park, S. (2017). A randomized controlled trial of physical activity, dietary habit, and distress management with the Leadership and Coaching for Health (LEACH) program for disease-free cancer survivors. *BMC Cancer,* **17**(1), 298. https://dx.doi.org/10.1186/s12885-017-3290-9

Zhang, H., Luiu, Y., & Shan, C. (2016). Effects of peer support on the prevention of postpartum depression. *Nursing Practice and Research,* **14**(58-60).

## 8 Link to online interactive EGM

[What is the volume, diversity & nature of recent, robust evidence for the use of peer support in health & social care? An evidence & gap map of recent randomised controlled trial (RCT) & systematic review (SR) evidence](https://eppi.ioe.ac.uk/cms/Portals/35/Maps/ExeterNIHR/PeerSupport)

## 9 Assessment of study quality

**AMSTAR 2**

*indicates key domains, used to determine the overall study rating

| + | Yes | ? | Partial Yes | - | No | **NA** | Not applicable |
| --- | --- | --- | --- | --- | --- | --- | --- |

| **Author** | **1** | **2*** | **3** | **4*** | **5** | **6** | **7*** | **8** | **9a*** | **9b*** | **10** | **11a*** | **11b*** | **12** | **13*** | **14** | **15*** | **16** | **Overall rating** |
| --- | --- | --- | --- | --- | --- | --- | --- | --- | --- | --- | --- | --- | --- | --- | --- | --- | --- | --- | --- |
| Ali (2015) | - | - | - | ? | + | + | ? | + | + | + | - | **NA** | **NA** | **NA** | + | + | **NA** | + | Low |
| Bassuk (2016) | + | + | + | ? | + | + | ? | + | + | + | - | **NA** | **NA** | **NA** | - | + | **NA** | - | Low |
| Beaudoin (2020) | + | - | - | ? | + | + | - | ? | + | + | - | **NA** | **NA** | **NA** | + | + | **NA** | + | Critically Low |
| Best (2016) | + | ? | - | ? | + | + | - | ? | + | **NA** | - | + | ? | + | + | + | + | - | Low |
| Boucher (2020) | + | + | + | ? | + | + | ? | + | ? | ? | + | **NA** | **NA** | **NA** | + | + | **NA** | + | High |
| Bryan (2015) | + | - | - | ? | - | + | ? | ? | + | + | - | + | **NA** | + | - | + | + | - | Critically Low |
| Burke (2019) | + | - | + | + | + | - | ? | ? | + | + | - | + | **NA** | + | + | + | + | - | Low |
| Cabassa (2017) | + | - | - | ? | + | + | ? | ? | ? | ? | - | **NA** | **NA** | **NA** | + | + | **NA** | + | Low |
| Chien (2019) | + | + | + | + | + | + | + | + | + | **NA** | + | + | **NA** | + | + | + | + | + | High |
| Gatlin (2017) | + | - | + | ? | - | - | - | ? | ? | **NA** | - | **NA** | **NA** | **NA** | + | - | **NA** | - | Critically Low |
| Genberg (2016) | + | - | + | ? | + | - | ? | ? | ? | + | - | **NA** | **NA** | **NA** | + | + | **NA** | - | Low |
| Haines (2018) | + | + | - | ? | + | + | - | + | + | + | - | **NA** | **NA** | **NA** | + | + | **NA** | + | Low |
| Huang (2020) | + | - | - | + | - | + | ? | + | + | **NA** | - | + | ? | + | + | + | + | + | Low |
| Hughes (2020) | - | - | + | ? | - | - | - | + | ? | + | - | **NA** | **NA** | **NA** | + | + | **NA** | + | Critically Low |
| Hunt (2019) | + | + | + | ? | + | + | ? | + | + | + | - | **NA** | **NA** | **NA** | + | + | **NA** | + | High |
| Kanters (2016) | + | ? | - | ? | + | + | ? | ? | + | **NA** | - | + | **NA** | - | - | + | - | + | Critically Low |
| Kelly (2020) | + | + | + | ? | + | + | + | + | + | + | + | + | + | + | + | + | + | + | High |
| Kong (2019) | + | + | - | ? | - | + | ? | + | + | **NA** | - | + | **NA** | + | + | + | + | + | Moderate |
| Kong (2020) | + | + | - | ? | + | + | ? | ? | + | **NA** | - | + | **NA** | + | + | + | + | + | Moderate |
| Krishnamoorthy (2019) | - | - | + | ? | + | - | ? | - | + | **NA** | - | + | **NA** | - | - | + | + | + | Critically Low |
| Lee (2018) | - | - | + | ? | + | - | - | ? | ? | ? | - | + | + | + | + | + | + | - | Critically Low |
| Levy (2019) | + | + | - | ? | + | - | ? | - | + | **NA** | - | **NA** | **NA** | **NA** | + | + | **NA** | + | Moderate |
| Liang (2021) | + | - | + | ? | + | + | ? | ? | + | **NA** | - | + | **NA** | + | + | - | + | + | Low |
| Maclachlan (2020) | + | + | - | ? | + | + | ? | ? | ? | + | - | **NA** | **NA** | **NA** | + | + | **NA** | + | Moderate |
| Meyer (2015) | + | - | + | ? | - | + | - | ? | - | - | - | **NA** | **NA** | **NA** | + | - | **NA** | - | Critically Low |
| Morris (2017) | + | + | - | + | + | + | ? | + | + | + | - | **NA** | **NA** | **NA** | + | + | **NA** | + | Moderate |
| Patil (2016) | + | ? | + | + | + | + | ? | + | + | **NA** | - | + | **NA** | + | + | + | + | + | High |
| Patil (2018) | + | - | + | ? | + | + | ? | ? | ? | **NA** | - | + | **NA** | - | + | + | + | + | Low |
| Qi (2015) | + | - | + | ? | + | + | ? | + | + | **NA** | - | + | **NA** | + | + | + | + | + | Low |
| White (2020) | + | + | - | + | + | - | ? | + | + | **NA** | - | + | **NA** | - | + | + | - | + | Low |
| Wobma (2016) | + | ? | - | ? | + | - | ? | ? | + | **NA** | - | **NA** | **NA** | **NA** | - | + | **NA** | - | Low |
| Zhang (2016) | - | - | - | + | + | + | ? | - | + | **NA** | - | + | **NA** | + | + | + | + | + | Moderate |

**ROB**

*indicates key domains, used to determine the overall study rating

| + | High | ? | Unclear | - | Low |
| --- | --- | --- | --- | --- | --- |

| **Author** | **Random sequence generation*** | **Allocation concealment** | **Blinding of participants and personnel** | **Blinding of outcome assessment** | **Incomplete outcome data*** | **Selective reporting*** | **Summary assessment** |
| --- | --- | --- | --- | --- | --- | --- | --- |
| Andreae (2021) | **+** | **+** | **-** | **-** | **+** | **?** | **?** |
| Cabassa (2020) | **?** | **?** | **-** | **?** | **+** | **+** | **?** |
| Cabral (2018) | **+** | **-** | **-** | **-** | **?** | **+** | **?** |
| Chang (2017) | **+** | **+** | **-** | **+** | **+** | **+** | **+** |
| Chien (2018) | **+** | **+** | **-** | **-** | **+** | **+** | **+** |
| Colella (2018) | **+** | **+** | **+** | **-** | **-** | **+** | **-** |
| Conley (2020) | **-** | **-** | **-** | **-** | **+** | **+** | **+** |
| Cook (2020) | **+** | **+** | **-** | **-** | **+** | **+** | **+** |
| Corrigan (2017) | **?** | **?** | **-** | **-** | **?** | **?** | **?** |
| Corrigan (2018) | **-** | **-** | **-** | **-** | **-** | **+** | **-** |
| Crisanti (2019) | **?** | **?** | **?** | **-** | **?** | **+** | **?** |
| Cunningham (2018) | **+** | **+** | **-** | **-** | **+** | **+** | **+** |
| Depping (2021) | **+** | **+** | **-** | **-** | **+** | **+** | **+** |
| Druss (2018) | **?** | **?** | **-** | **-** | **+** | **+** | **?** |
| Easter (2020) | **+** | **?** | **-** | **-** | **?** | **+** | **?** |
| Ellison (2020) | **-** | **-** | **-** | **-** | **-** | **+** | **-** |
| Fraser (2015) | **+** | **?** | **-** | **-** | **+** | **+** | **+** |
| Gassaway (2017) | **+** | **+** | **-** | **?** | **?** | **+** | **?** |
| Hart (2021) | **?** | **?** | **-** | **-** | **?** | **+** | **?** |
| Hilari (2021) | **+** | **+** | **-** | **-** | **+** | **+** | **+** |
| Houlihan (2017) | **+** | **+** | **-** | **-** | **+** | **+** | **+** |
| Jamison (2017) | **?** | **-** | **-** | **-** | **+** | **+** | **?** |
| Johnson (2020) | **+** | **+** | **-** | **-** | **+** | **+** | **+** |
| Kelly (2017) | **+** | **+** | **-** | **-** | **+** | **+** | **+** |
| Kidd (2021) | **+** | **+** | **-** | **-** | **?** | **+** | **?** |
| Kyaw (2021) | **+** | **?** | **-** | **-** | **?** | **+** | **?** |
| Lara-Cabrera (2016) | **+** | **+** | **-** | **-** | **+** | **+** | **+** |
| Larsen (2019) | **+** | **+** | **-** | **-** | **+** | **+** | **+** |
| Leahey (2016) | **+** | **+** | **-** | **+** | **+** | **+** | **+** |
| Leahey (2020) | **+** | **+** | **-** | **+** | **+** | **+** | **+** |
| Long (2020) | **+** | **+** | **-** | **+** | **+** | **+** | **+** |
| Mathews (2018) | **+** | **+** | **-** | **-** | **-** | **+** | **-** |
| Matthias (2020) | **+** | **?** | **-** | **-** | **+** | **+** | **+** |
| Mayer (2019) | **-** | **-** | **-** | **+** | **?** | **+** | **-** |
| Mehlsen (2017) | **+** | **+** | **-** | **-** | **+** | **+** | **+** |
| Muralidharan (2019) | **?** | **?** | **-** | **-** | **-** | **+** | **-** |
| Napoles (2020) | **+** | **+** | **-** | **-** | **+** | **+** | **+** |
| Nyamathi (2015) | **+** | **?** | **-** | **+** | **?** | **?** | **?** |
| O'Connell (2020) | **?** | **?** | **-** | **-** | **-** | **+** | **-** |
| Piatt (2018) | **-** | **-** | **-** | **-** | **?** | **-** | **-** |
| Rao (2018) | **+** | **?** | **-** | **-** | **-** | **+** | **-** |
| Sampson (2021) | **+** | **+** | **-** | **+** | **+** | **+** | **+** |
| Sanders (2020) | **+** | **+** | **-** | **-** | **-** | **+** | **-** |
| Spencer (2018) | **+** | **+** | **-** | **-** | **+** | **?** | **?** |
| Stagg (2019) | **+** | **+** | **-** | **+** | **+** | **+** | **+** |
| Sullivan (2018) | **+** | **+** | **?** | **+** | **+** | **+** | **+** |
| Toija (2019) | **-** | **-** | **-** | **-** | **+** | **+** | **-** |
| Vagharseyyedin (2017) | **+** | **+** | **-** | **-** | **?** | **+** | **?** |
| Valenstein (2016) | **+** | **+** | **-** | **-** | **?** | **+** | **?** |
| Wang (2018) | **+** | **+** | **-** | **-** | **+** | **+** | **+** |
| Ward (2019) | **+** | **+** | **-** | **+** | **+** | **+** | **+** |
| Yoon (2017) | **-** | **+** | **-** | **+** | **?** | **+** | **-** |

**CHEC**

| + | Yes | - | No | **NA** | Not applicable |
| --- | --- | --- | --- | --- | --- |

| **Author** | **1** | 2 | 3 | 4 | 5 | 6 | 7 | 8 | 9 | 10 | 11 | 12 | 13 | 14 | 15 | 16 | 17 | 18 | 19 | **Overall rating** |
| --- | --- | --- | --- | --- | --- | --- | --- | --- | --- | --- | --- | --- | --- | --- | --- | --- | --- | --- | --- | --- |
| Campbell 2015 | **+** | **+** | **+** | **+** | **+** | **+** | **+** | **+** | **+** | **+** | **+** | **-** | **+** | **NA** | **-** | **+** | **-** | **+** | **-** | High |
| Hodgkin 2019 | **+** | **+** | **+** | **-** | **NA** | **-** | **-** | **-** | **+** | **NA** | **NA** | **NA** | **NA** | **NA** | **-** | **+** | **-** | **+** | **-** | Low |
| Patel 2017 | **-** | **-** | **+** | **+** | **+** | **-** | **+** | **+** | **+** | **+** | **+** | **-** | **-** | **+** | **+** | **+** | **-** | **+** | **-** | Medium |
| Williams 2019 | **+** | **-** | **+** | **+** | **-** | **-** | **-** | **-** | **-** | **+** | **+** | **NA** | **+** | **NA** | **-** | **-** | **-** | **+** | **-** | Low |
| Wingate 2017 | **+** | **-** | **+** | **-** | **+** | **+** | **+** | **+** | **+** | **NA** | **NA** | **NA** | **NA** | **NA** | **-** | **+** | **-** | **+** | **-** | Medium |
| Ye 2021 | **+** | **+** | **+** | **+** | **+** | **-** | **+** | **-** | **-** | **+** | **+** | **+** | **+** | **+** | **+** | **+** | **-** | **+** | **-** | Medium |
| Yu 2021 | **+** | **+** | **+** | **-** | **+** | **NA** | **-** | **+** | **+** | **NA** | **NA** | **NA** | **NA** | **NA** | **-** | **+** | **-** | **+** | **-** | Medium |
