# Supplementary material for: What is the volume, diversity and nature of recent, robust evidence for the use of peer support in health and social care? An evidence and gap map
Source: Campbell Syst Rev. 2022 Jul 26;18(3):e1264. doi: 10.1002/cl2.1264 (PMC9316011; doi:10.1002/cl2.1264)
Supplement: Supplementary file 2 — Supporting information. [file CL2-18-e1264-s002.docx]

**Abbreviations and acronyms**

**AMSTAR 2** assessing the methodological quality of systematic reviews

**CC** comparative costing

**CEA** cost-effectiveness analysis

**CHEC** consensus health economic criteria list

**CM** case management

**CUA** cost-utility analysis

**EE** economic evaluation

**EGM** evidence and gap map

**HB** health behaviour

**HCV** hepatitis C virus

**HIV** human immunodeficiency virus

**HS** health service

**HSDR** Health Services and Delivery Research

**MH** mental health

**NIHR** National Institute for Health Research

**PH** physical health

**PICO** population, interventions, comparison type, and outcomes

**PPI** public patient involvement

**PRISMA** preferred reporting items for systematic reviews and meta-analyses

**RCT** randomised controlled trial

**ROB** Cochrane risk of bias tool

**SR** systematic review
