# Supplementary material for: What is the volume, diversity and nature of recent, robust evidence for the use of peer support in health and social care? An evidence and gap map
Source: Campbell Syst Rev. 2022 Jul 26;18(3):e1264. doi: 10.1002/cl2.1264 (PMC9316011; doi:10.1002/cl2.1264)
Supplement: Supplementary file 3 — Supporting information. [file CL2-18-e1264-s003.docx]

**Studies included in the EGM**

Ali, K., Farrer, L., Gulliver, A., & Griffiths, K. M. (2015). Online Q7 peer‐to‐peer support for young people with mental health problems: A systematic review. *JMIR Mental Health*, *2*(2), e19. https://doi.org/10.2196/mental.4418

Bassuk, E. L., Hanson, J., Greene, R. N., Richard, M., & Laudet, A. (2016). Peer‐delivered recovery support services for addictions in the United States: A systematic review. *Journal of Substance* *Abuse Treatment*, *63*, 1–9. https://doi.org/10.1016/j.jsat.2016.01.003

Beaudoin, M., Best, K. L., & Routhier, F. (2020). Influence of peer‐based rehabilitation interventions for improving mobility and participation among adults with mobility disabilities: A systematic review. *Disability and Rehabilitation*, *42*(13), 1785–1796. <https://doi.org/10.1080/09638288.2018.1537380>

Best, K. L., Miller, W. C., Eng, J. J., & Routhier, F. (2016). Systematic review and meta‐analysis of peer‐led self‐management programs for increasing physical activity. *International Journal of Behavioral* *Medicine*, *23*(5), 527–538. <https://doi.org/10.1007/s12529-016-9540-4>

Boucher, L. M., Liddy, C., Mihan, A., & Kendall, C. (2020). Peer‐led selfmanagement interventions and adherence to antiretroviral therapy among people living with HIV: A systematic review. *AIDS and* *Behavior*, *24*(4), 998–1022. <https://doi.org/10.1007/s10461-019-02690-7>

Bryan, A. E. B., & Arkowitz, H. (2015). Meta‐analysis of the effects of peer‐administered psychosocial interventions on symptoms of

depression. *American Journal of Community Psychology*, *55*(3‐4), 455–471. <https://doi.org/10.1007/s10464-015-9718-y>

Burke, E., Pyle, M., Machin, K., Varese, F., & Morrison, A. P. (2019). The effects of peer support on empowerment, self‐efficacy, and internalized stigma: A narrative synthesis and meta‐analysis. *Stigma and Health*, *4*(3), 337–356. https://doi.org/10.1037/sah0000148

Cabassa, L. J., Camacho, D., Velez‐Grau, C. M., & Stefancic, A. (2017). Peer‐based health interventions for people with serious mental

illness: A systematic literature review. *Journal of Psychiatric Research*, *84*, 80–89. <https://doi.org/10.1016/j.jpsychires.2016.09.021>

Chien, W. T., Clifton, A. V., Zhao, S., & Lui, S. (2019). Peer support for people with schizophrenia or other serious mental illness. *The* *Cochrane Database of Systematic Reviews*, *4*, CD010880. <https://doi.org/10.1002/14651858.CD010880.pub2>

Gatlin, T. K., Serafica, R., & Johnson, M. (2017). Systematic review of peer education intervention programmes among individuals with type 2 diabetes. *Journal of Clinical Nursing*, *26*(23‐24), 4212–4222. https://doi.org/10.1111/jocn.13991

Genberg, B. L., Shangani, S., Sabatino, K., Rachlis, B., Wachira, J., Braitstein, P., & Operario, D. (2016). Improving engagement in the

HIV care cascade: A systematic review of interventions involving people living with HIV/AIDS as peers. *AIDS and Behavior*, *20*(10),

2452–2463. <https://doi.org/10.1007/s10461-016-1307-z>

Haines, K. J., Beesley, S. J., Hopkins, R. O., McPeake, J., Quasim, T., Ritchie, K., & Iwashyna, T. J. (2018). Peer support in critical care: A

systematic review. *Critical Care Medicine*, *46*(9), 1522–1531. <https://doi.org/10.1097/CCM.0000000000003293>

Huang, R., Yan, C., Tian, Y., Lei, B., Yang, D., Liu, D., & Lei, J. (2020). Effectiveness of peer support intervention on perinatal depression: A systematic review and meta‐analysis. *Journal of Affective Disorders*, *276*, 788–796. <https://doi.org/10.1016/j.jad.2020.06.048>

Hughes, R., Fleming, P., & Henshall, L. (2020). Peer support groups after acquired brain injury: A systematic review. *Brain Injury*,

*34*(7), 847–856. https://doi.org/10.1080/02699052.2020.1762002

Hunt, H., Abbott, R., Boddy, K., Whear, R., Wakely, L., Bethel, A., Morris, C., Prosser, S., Collinson, A., Kurinczuk, J., & Thompson‐ Coon, J. (2019). "They've walked the walk": A systematic review of quantitative and qualitative evidence for parent‐to‐parent support

for parents of babies in neonatal care. *Journal of Neonatal Nursing*, *25*(4), 166–176. <https://doi.org/10.1016/j.jnn.2019.03.011>

Kanters, S., Park, J. J., Chan, K., Ford, N., Forrest, J., Thorlund, K., Nachega, J. B., & Mills, E. J. (2016). Use of peers to improve

adherence to antiretroviral therapy: A global network meta‐analysis. *Journal of the International AIDS Society*, *19*(1), 21141. https://doi.

org/10.7448/IAS.19.1.21141

Kelly, J. F., Humphreys, K., & Ferri, M. (2020). Alcoholics anonymous and other 12‐step programs for alcohol use disorder. *The Cochrane*

*Database of Systematic Reviews*, *3*, CD012880. https://doi.org/10.1002/14651858.CD012880.pub2

Kong, L.‐N., Hu, P., Yang, L., & Cui, D. (2019). The effectiveness of peer support on self‐efficacy and quality of life in adults with type 2

diabetes: A systematic review and meta‐analysis. *Journal of Advanced Nursing*, *75*(4), 711–722. https://doi.org/10.1111/jan.13870

Kong, L.‐N., Hu, P., Zhao, Q.‐H., Yao, H.‐Y., & Chen, S.‐Z. (2020). Effect of peer support intervention on diabetes distress in people with type 2 diabetes: A systematic review and meta‐analysis. *International* *Journal of Nursing Practice*, *26*(5), e12830. https://doi.org/10.1111/

ijn.12830

Krishnamoorthy, Y., Sakthivel, M., Sarveswaran, G., & Eliyas, S. K. (2019). Effectiveness of peer led intervention in improvement of clinical

outcomes among diabetes mellitus and hypertension patients—A systematic review and meta‐analysis. *Primary Care Diabetes*, *13*(2),

158–169. <https://doi.org/10.1016/j.pcd.2018.11.007>

Lee, M. K., & Suh, S.‐R. (2018). Effects of peer‐led interventions for patients with cancer: A meta‐analysis. *Oncology Nursing Forum*,

*45*(2), 217–236. <https://doi.org/10.1188/18.ONF.217-236>

Levy, B. B., Luong, D., Perrier, L., Bayley, M. T., & Munce, S. E. P. (2019). Peer support interventions for individuals with acquired brain injury, cerebral palsy, and spina bifida: a systematic review. *BMC Health* *Services Research*, *19*(1), 288. https://doi.org/10.1186/s12913-019-

4110-5

Liang, D., Jia, R., Zhou, X., Lu, G., Wu, Z., Yu, J., Wang, Z., Huang, H., Guo, J., & Chen, C. (2021). The effectiveness of peer support on selfefficacy and self‐management in people with type 2 diabetes: A meta‐analysis. *Patient Education & Counseling*, *104*(4), 760–769.

<https://doi.org/10.1016/j.pec.2020.11.011>

Maclachlan, L. R., Mills, K., Lawford, B. J., Egerton, T., Setchell, J., Hall, L. M., Plinsinga, M. L., Besomi, M., Teo, P. L., Eyles, J. P.,

Mellor, R., Melo, L., Robbins, S., Hodges, P. W., Hunter, D. J., Vicenzino, B., & Bennell, K. L. (2020). Design, delivery, maintenance,

and outcomes of peer‐to‐peer online support groups for people with chronic musculoskeletal disorders: Systematic review. *Journal of*

*Medical Internet Research*, *22*(4), e15822. https://doi.org/10.2196/15822

Meyer, A., Coroiu, A., & Korner, A. (2015). One‐to‐one peer support in cancer care: A review of scholarship published between 2007 and

2014. *European Journal of Cancer Care*, *24*(3), 299–312. https://doi.org/10.1111/ecc.12273

Morris, R., Fletcher‐Smith, J., & Radford, K. (2017). A systematic review of peer mentoring interventions for people with traumatic brain injury. *Clinical Rehabilitation*, *31*(3), 426–427. https://doi.org/10.1177/0269215516676303

Patil, S. J., Ruppar, T., Koopman, R. J., Lindbloom, E. J., Elliott, S. G., Mehr, D. R., & Conn, V. S. (2016). Peer support interventions for

adults with diabetes: A meta‐analysis of hemoglobin A1C outcomes. *Annals of Family Medicine*, *14*(6), 540–551. https://doi.org/10.1370/

afm.1982

Patil, S. J., Ruppar, T., Koopman, R. J., Lindbloom, E. J., Elliott, S. G., Mehr, D. R., & Conn, V. S. (2018). Effect of peer support

interventions on cardiovascular disease risk factors in adults with diabetes: A systematic review and meta‐analysis. *BMC Public Health*,

*18*(1), 398. <https://doi.org/10.1186/s12889-018-5326-8>

Qi, L., Liu, Q., Qi, X., Wu, N., Tang, W., & Xiong, H. (2015). Effectiveness of peer support for improving glycaemic control in patients with type 2 diabetes: A meta‐analysis of randomized controlled trials. *BMC* *Public Health*, *15*, 471. <https://doi.org/10.1186/s12889-015-1798-y>

White, S., Foster, R., Marks, J., Morshead, R., & Goldsmith, L. (2020). The effectiveness of one‐to‐one peer support in mental health services: a systematic review and meta‐analysis. *BMC Psychiatry*, *20*(1), 534. <https://doi.org/10.1186/s12888-020-02923-3>

Wobma, R., Nijland, R. H. M., Ket, J. C. F., & Kwakkel, G. (2016). Evidence for peer support in rehabilitation for individuals with acquired brain injury: A systematic review. *Journal of Rehabilitation Medicine*, *48*(10), 837–840. <https://doi.org/10.2340/16501977-2160>

Zhang, X., Yang, S., Sun, K., Fisher, E. B., & Sun, X. (2016). How to achieve better effect of peer support among adults with type 2 diabetes: A meta‐analysis of randomized clinical trials. *Patient Education &* *Counseling*, *99*(2), 186–197. https://doi.org/10.1016/j.pec.2015.09.

006

Andreae, S. J., Andreae, L. J., Cherrington, A. L., Richman, J. S., Johnson, E., Clark, D., & Safford, M. M. (2021). Peer coach delivered storytelling program improved diabetes medication adherence: A cluster randomized trial. *Contemporary Clinical Trials*, *104*, 106358.

https://doi.org/10.1016/j.cct.2021.106358

Cabassa, L. J., Stefancic, A., Lewis‐Fernandez, R., Luchsinger, J., Weinstein, L. C., Guo, S., Palinkas, L., Bochicchio, L., Wang, X.,

O'Hara, K., Blady, M., Simiriglia, C., & Medina McCurdy, M. (2020). Main outcomes of a peer‐led healthy lifestyle Q8 intervention for

people with serious mental illness in supportive housing. *Psychiatric Services , 72*, 555-562. https://doi.org/10.1176/appi.

ps.202000304

Cabral, H. J., Davis‐Plourde, K., Sarango, M., Fox, J., Palmisano, J., & Rajabiun, S. (2018). Peer support and the HIV continuum of care:

Results from a multi‐site randomized clinical trial in three urban clinics in the United States. *AIDS and Behavior*, *22*(8), 2627–2639.

<https://doi.org/10.1007/s10461-017-1999-8>

Chang, M. W., Brown, R., & Nitzke, S. (2017). Results and lessons learned from a prevention of weight gain program for low‐income overweight and obese young mothers: Mothers in motion. *BMC Public* *Health*, *17*(1), 182. https://doi.org/10.1186/s12889-017-4109-y

Chien, W. T., Bressington, D., & Chan, S. W. C. (2018). A randomized controlled trial on mutual support group intervention for families of

people with recent‐onset psychosis: A four‐year follow‐up. *Frontiers in Psychiatry*, *9*, 710. https://doi.org/10.3389/fpsyt.2018.00710

Colella, T. J., & King‐Shier, K. (2018). The effect of a peer support intervention on early recovery outcomes in men recovering from

coronary bypass surgery: A randomized controlled trial. *European Journal of Cardiovascular Nursing*, *17*(5), 408–417. https://doi.org/

10.1177/1474515117725521

Conley, C. S., Hundert, C. G., Charles, J. L., Huguenel, B. M., Al‐khouja, M., Qin, S., Paniagua, D., & Corrigan, P. W. (2020). Honest, open, proudcollege: Effectiveness of a peer‐led small‐group intervention for reducing the stigma of mental illness. *Stigma and Health*, *5*(2), 168–178. https://doi.org/10.1037/sah0000185

Cook, J. A., Jonikas, J. A., Burke‐Miller, J. K., Hamilton, M., Powell, I. G., Tucker, S. J., Wolfgang, J. B., Fricks, L., Weidenaar, J., Morris, E., & Powers, D. L. (2020). Whole Health Action Management: A randomized controlled trial of a peer‐led health promotion intervention.

*Psychiatric Services*, *71*(10), 1039–1046. https://doi.org/10.1176/appi.ps.202000012

Corrigan, P., Sheehan, L., Morris, S., Larson, J. E., Torres, A., Lara, J. L., Paniagua, D., Mayes, J. I., & Doing, S. (2018). The impact of a peer navigator program in addressing the health needs of latinos with serious mental illness. *Psychiatric Services*, *69*(4), 456–461. https://

doi.org/10.1176/appi.ps.201700241

Corrigan, P. W., Kraus, D. J., Pickett, S. A., Schmidt, A., Stellon, E., Hantke, E., & Lara, J. L. (2017). Using peer navigators to address the

integrated health care needs of homeless African Americans with serious mental illness. *Psychiatric Services*, *68*(3), 264–270. https://

doi.org/10.1176/appi.ps.201600134

Crisanti, A. S., Murray‐Krezan, C., Reno, J., & Killough, C. (2019). Effectiveness of peer‐delivered trauma treatment in a rural

community: A randomized non‐inferiority trial. *Community Mental Health Journal*, *55*(7), 1125–1134. https://doi.org/10.1007/s10597-

019-00443-3

Cunningham, W. E., Weiss, R. E., Nakazono, T., Malek, M. A., Shoptaw, S. J., Ettner, S. L., & Harawa, N. T. (2018). Effectiveness

of a peer navigation intervention to sustain viral suppression among HIV‐positive men and transgender women released from jail: The

LINK LA randomized clinical trial. *JAMA Internal Medicine*, *178*(4), 542–553. https://doi.org/10.1001/jamainternmed.2018.0150

Depping, M. K., Uhlenbusch, N., Harter, M., Schramm, C., & Lowe, B. (2021). Efficacy of a brief, peer‐delivered self‐management intervention for patients with rare chronic diseases: A randomized clinical trial. *JAMA Psychiatry*, *24*, 24. https://doi.org/10.1001/

jamapsychiatry.2020.4783

Druss, B. G., Singh, M., von Esenwein, S. A., Glick, G. E., Tapscott, S., Tucker, S. J., Lally, C. A., & Sterling, E. W. (2018). Peer‐led selfmanagement of general medical conditions for patients with serious mental illnesses: A randomized trial. *Psychiatric Services*, *69*(5),

529–535. https://doi.org/10.1176/appi.ps.201700352

Easter, M. M., Swanson, J. W., Robertson, A. G., Moser, L. L., & Swartz, M. S. (2020). Impact of psychiatric advance directive

facilitation on mental Q9 health consumers: Empowerment, treatment attitudes and the role of peer support specialists. *Journal of Mental*

*Health*, *30,* 1–9. https://doi.org/10.1080/09638237.2020.1714008

Ellison, M. L., Schutt, R. K., Yuan, L. H., Mitchell‐Miland, C., Glickman, M. E., McCarthy, S., Smelson, D., Schultz, M. R., & Chinman, M. (2020). Impact of peer specialist services on residential stability and behavioral health status among formerly homeless veterans with cooccurring mental health and substance use conditions. *Medical Care*, *58*(4), 307–313. https://doi.org/10.1097/MLR.0000000000001284

Fraser, R. T., Johnson, E. K., Lashley, S., Barber, J., Chaytor, N., Miller, J. W., Ciechanowski, P., Temkin, N., & Caylor, L. (2015). PACES in epilepsy: Results of a self‐management randomized controlled trial. *Epilepsia*, *56*(8), 1264–1274. https://doi.org/10.1111/epi.13052

Gassaway, J., Jones, M. L., Sweatman, W. M., Hong, M., Anziano, P., & DeVault, K. (2017). Effects of peer mentoring on self‐efficacy and

hospital readmission after inpatient rehabilitation of individuals with spinal cord injury: A randomized controlled trial. *Archives of Physical*

*Medicine & Rehabilitation*, *98*(8), 1526–1534.e1522. <https://doi.org/> 10.1016/j.apmr.2017.02.018

Hart, T. A., Noor, S. W., Skakoon‐Sparling, S., Lazkani, S. N., Gardner, S., Leahy, B., Maxwell, J., Julien, R., Simpson, S., Steinberg, M., &

Adam, B. D. (2021). Gps: A randomized controlled trial of sexual health counseling for gay and bisexual men living with HIV. *Behavior Therapy*, *52*(1), 1–14. https://doi.org/10.1016/j.beth.2020.04.005

Hilari, K., Behn, N., James, K., Northcott, S., Marshall, J., Thomas, S., Simpson, A., Moss, B., Flood, C., McVicker, S., & Goldsmith, K.

(2021). Supporting wellbeing through peer‐befriending (SUPERB) for people with aphasia: A feasibility randomised controlled trial. *Clinical*

*Rehabilitation*, *35*. https://doi.org/10.1177/0269215521995671

Houlihan, B. V., Brody, M., Everhart‐Skeels, S., Pernigotti, D., Burnett, S., Zazula, J., Green, C., Hasiotis, S., Belliveau, T., Seetharama, S.,

Rosenblum, D., & Jette, A. (2017). Randomized trial of a peer‐led, telephone‐based empowerment intervention for persons with chronic spinal cord injury improves health self‐management. *Archives of Physical Medicine & Rehabilitation*, *98*(6), 1067–1076.

https://doi.org/10.1016/j.apmr.2017.02.005

Jamison, J. M., Fourie, E., Siper, P. M., Trelles, M. P., George‐Jones, J., Buxbaum Grice, A., Krata, J., Holl, E., Shaoul, J., Hernandez, B.,

Mitchell, L., McKay, M. M., Buxbaum, J. D., & Kolevzon, A. (2017). Examining the efficacy of a family peer advocate model for black and

Hispanic caregivers of children with autism spectrum disorder. *Journal of Autism & Developmental Disorders*, *47*(5), 1314–1322.

https://doi.org/10.1007/s10803-017-3045-0

Johnson, E. K., Fraser, R. T., Lashley, S., Barber, J., Brandling‐Bennett, E. M., Vossler, D. G., Miller, J. W., Caylor, L., & Warheit‐Niemi, T.

(2020). Program of active consumer engagement in selfmanagement in epilepsy: Replication and extension of a selfmanagement

randomized controlled trial. *Epilepsia*, *61*(6), 1129–1141. https://doi.org/10.1111/epi.16530

Kelly, E., Duan, L., Cohen, H., Kiger, H., Pancake, L., & Brekke, J. (2017). Integrating behavioral healthcare for individuals with serious mental illness: A randomized controlled trial of a peer health navigator intervention. *Schizophrenia Research*, *182*, 135–141. https://doi.org/

10.1016/j.schres.2016.10.031

Kidd, S. A., Mutschler, C., Lichtenstein, S., Yan, S., Virdee, G., Blair, F., Mihalakakos, G., McKinney, C., Collins, A., Guimond, T.,

George, T. P., Davidson, L., Velligan, D., & Voineskos, A. (2021). Randomized trial of a brief peer support intervention for individuals

with schizophrenia transitioning from hospital to community. *Schizophrenia Research*, *231*, 214–220. https://doi.org/10.1016/j.

schres.2021.03.019

Kyaw Tha Tun, E., Nagel, J., Bosbach, A., Bock, S., Kielblock, B., Siegmund‐Schultze, E., & Herrmann‐Lingen, C. (2021). Telephone‐based peer support intervention to reduce depressive symptoms in women with coronary heart disease, a randomized controlled trial in Germany.

*Women and Health*, *61*(7), 619–632. https://doi.org/10.1080/03630242.2021.1953208

Lara‐Cabrera, M. L., Gjerden, M., Grawe, R. W., Linaker, O. M., & Steinsbekk, A. (2016). Short‐term effects of a peer co‐led educational programme delivered before mental health treatment: A randomised controlled trial. *Patient Education & Counseling*, *99*(7), 1257–1261. <https://doi.org/10.1016/j.pec.2016.02.006>

Larsen, I. G., Gregersen Oestergaard, L., Thomsen, L. M., Vinther Nielsen, C., & Schiottz‐Christensen, B. (2019). Effect of adding lay‐tutors to the educational part of a back school programme for patients with subacute, non‐specific low back pain: A randomized controlled clinical trial with a two‐year follow‐up. *Journal of Rehabilitation Medicine*, *51*(9), 698–704. https://doi.org/10.2340/16501977-2584

Leahey, T. M., Fava, J. L., Seiden, A., Fernandes, D., Doyle, C., Kent, K., La Rue, M., Mitchell, M., & Wing, R. R. (2016). A randomized

controlled trial testing an Internet delivered cost‐benefit approach to weight loss maintenance. *Preventive Medicine*, *92*, 51–57. https://

doi.org/10.1016/j.ypmed.2016.04.013

Leahey, T. M., Huedo‐Medina, T. B., Grenga, A., Gay, L., Fernandes, D., Denmat, Z., Doyle, C., Areny‐Joval, R., & Wing, R. R. (2020). Patientprovided e‐support in reduced intensity obesity treatment: The INSPIRE randomized controlled trial. *Health Psychology*, *39*(12),

1037–1047. <https://doi.org/10.1037/hea0000996>

Long, J. A., Ganetsky, V. S., Canamucio, A., Dicks, T. N., Heisler, M., & Marcus, S. C. (2020). Effect of peer mentors in diabetes selfmanagement vs usual care on outcomes in us veterans with type 2 diabetes: A randomized clinical trial. *JAMA Network Open*, *3*(9),

e2016369. <https://doi.org/10.1001/jamanetworkopen.2020.16369>

Mathews, C. A., Mackin, R. S., Chou, C. Y., Uhm, S. Y., Bain, L. D., Stark, S. J., Gause, M., Vigil, O. R., Franklin, J., Salazar, M., Plumadore, J., Smith, L. C., Komaiko, K., Howell, G., Vega, E., Chan, J., Eckfield, M. B., Tsoh, J. Y., & Delucchi, K. (2018). Randomised clinical trial of community‐based peer‐led and psychologist‐led group treatment for hoarding disorder. *BJPsych* *Open*, *4*(4), 285–293. <https://doi.org/10.1192/bjo.2018.30>

Matthias, M. S., Bair, M. J., Ofner, S., Heisler, M., Kukla, M., McGuire, A. B., Adams, J., Kempf, C., Pierce, E., Menen, T., McCalley, S., Johnson, N. L., & Daggy, J. (2020). Peer support for selfmanagement of chronic pain: The evaluation of a peer coach‐led intervention to improve pain symptoms (ECLIPSE) trial. *Journal of* *General Internal Medicine*, *35*(12), 3525–3533. https://doi.org/10.1007/s11606-020-06007-6

Mayer, V. L., Vangeepuram, N., Fei, K., Hanlen‐Rosado, E. A., Arniella, G., Negron, R., Fox, A., Lorig, K., & Horowitz, C. R. (2019). Outcomes of a weight loss intervention to prevent diabetes among low‐income residents of East Harlem, New York. *Health Education & Behavior*, *46*(6), 1073–1082. <https://doi.org/10.1177/1090198119868232>

Mehlsen, M., Hegaard, L., Ornbol, E., Jensen, J. S., Fink, P., & Frostholm, L. (2017). The effect of a lay‐led, group‐based self‐management

program for patients with chronic pain: A randomized controlled trial of the Danish version of the Chronic Pain Self‐Management Programme. *Pain*, *158*(8), 1437–1445. https://doi.org/10.1097/j.pain.0000000000000931

Muralidharan, A., Brown, C. H., Peer, J., Klingman, E. A., Hack, S. M., Li, L., Walsh, M. B., & Goldberg, R. W. (2019). Living Well: An intervention to improve medical illness self‐management among individuals with serious mental illness. *Psychiatric Services*, *70*(1), 19–25. https://doi.org/10.1176/appi.ps.201800162

Napoles, A. M., Santoyo‐Olsson, J., Stewart, A. L., Ortiz, C., Samayoa, C., Torres‐Nguyen, A., Palomino, H., Coleman, L., Urias, A., Gonzalez, N., Cervantes, S. A., & Totten, V. Y. (2020). Nuevo Amanecer‐II: Results of a randomized controlled trial of a community‐based participatory, peer‐delivered stress management intervention for rural Latina breast cancer survivors. *Psycho‐Oncology*, *29*(11), 1802–1814.

<https://doi.org/10.1002/pon.5481>

Nyamathi, A., Salem, B. E., Zhang, S., Farabee, D., Hall, B., Khalilifard, F., & Leake, B. (2015). Nursing case management, peer coaching, and hepatitis A and B vaccine completion among homeless men recently released on parole: randomized clinical trial. *Nursing Research*, *64*(3), 177–189. <https://doi.org/10.1097/NNR.0000000000000083>

O'Connell, M. J., Flanagan, E. H., Delphin‐Rittmon, M. E., & Davidson, L. (2020). Enhancing outcomes for persons with co‐occurring disorders through skills training and peer recovery support. *Journal of Mental* *Health*, *29*(1), 6–11. <https://doi.org/10.1080/09638237.2017.1294733>

Piatt, G. A., Rodgers, E. A., Xue, L., & Zgibor, J. C. (2018). Integration and utilization of peer leaders for diabetes self‐management support:

Results from Project SEED (Support, Education, and Evaluation in Diabetes). *Diabetes Educator*, *44*(4), 373–382. <https://doi.org/10>. 1177/0145721718777855

Rao, D., Kemp, C. G., Huh, D., Nevin, P. E., Turan, J., Cohn, S. E., Simoni, J. M., Andrasik, M., Molina, Y., Mugavero, M. J., & French, A. L. (2018). Stigma reduction among African American women with HIV: UNITY Health Study. *Journal of Acquired Immune* *Deficiency Syndromes: JAIDS*, *78*(3), 269–275. https://doi.org/10.1097/QAI.0000000000001673

Sampson, M., Clark, A., Bachmann, M., Garner, N., Irvine, L., Howe, A., Greaves, C., Auckland, S., Smith, J., Turner, J., Rea, D., Rayman, G., Dhatariya, K., John, W. G., Barton, G., Usher, R., Ferns, C., Pascale, M., & Norfolk Diabetes Prevention Study Group. (2021). Lifestyle intervention with or without lay volunteers to prevent type 2 diabetes in people with impaired fasting glucose and/or nondiabetic hyperglycemia: A randomized clinical trial. *JAMA* *Internal Medicine*, *181*(2), 168–178. <https://doi.org/10.1001/jamainternmed.2020.5938>

Sanders, M., Tobin, J. N., Cassells, A., Carroll, J., Holder, T., Thomas, M., Luque, A., & Fiscella, K. (2020). Can a brief peer‐led group training intervention improve health literacy in persons living with HIV? Results from a randomized controlled trial. *Patient Education &*

*Counseling*, *31*, 31. <https://doi.org/10.1016/j.pec.2020.10.031>

Spencer, M. S., Kieffer, E. C., Sinco, B., Piatt, G., Palmisano, G., Hawkins, J., Lebron, A., Espitia, N., Tang, T., Funnell, M., & Heisler, M. (2018). Outcomes at 18 months from a community health worker and peer leader diabetes self‐management program for Latino adults. *Diabetes* *Care*, *41*(7), 1414–1422. <https://doi.org/10.2337/dc17-0978>

Stagg, H. R., Surey, J., Francis, M., MacLellan, J., Foster, G. R., Charlett, A., & Abubakar, I. (2019). Improving engagement with healthcare in hepatitis C: A randomised controlled trial of a peer support intervention. *BMC Medicine*, *17*(1), 71. https://doi.org/10.1186/s12916-019-1300-2

Sullivan, C. M., Barnswell, K. V., Greenway, K., Kamps, C. M., Wilson, D., Albert, J. M., Dolata, J., Huml, A., Pencak, J. A., Ducker, J. T.,

Gedaly, R., Jones, C. M., Pesavento, T., & Sehgal, A. R. (2018). Impact of navigators on first visit to a transplant center, waitlisting, and

kidney transplantation: A randomized, controlled trial. *Clinical Journal of the American Society of Nephrology*, *13*(10), 1550–1555. https://

doi.org/10.2215/CJN.03100318

Toija, A. S., Kettunen, T. H., Leidenius, M. H. K., Vainiola, T. H. K., & Roine, R. P. A. (2019). Effectiveness of peer support on healthrelated

quality of life in recently diagnosed breast cancer patients: A randomized controlled trial. *Supportive Care in Cancer*, *27*(1), 123–130. <https://doi.org/10.1007/s00520-018-4499-0>

Vagharseyyedin, S. A., Gholami, M., Hajihoseini, M., & Esmaeili, A. (2017). The effect of peer support groups on family adaptation from the

perspective of wives of war veterans with posttraumatic stress disorder. *Public Health Nursing*, *34*(6), 547–554. https://doi.org/10.

1111/phn.12349

Valenstein, M., Pfeiffer, P. N., Brandfon, S., Walters, H., Ganoczy, D., Kim, H. M., Cohen, J. L., Benn‐Burton, W., Carroll, E., Henry, J.,

Garcia, E., Risk, B., Kales, H. C., Piette, J. D., & Heisler, M. (2016). Augmenting ongoing depression care with a mutual peer support intervention versus self‐help materials alone: A randomized trial. *Psychiatric Services*, *67*(2), 236–239. https://doi.org/10.1176/appi.

ps.201400454

Wang, J. (2018). *Loneliness and mental health in a randomized controlled trial of a peer‐provided self‐management intervention for people leaving crisis resolution teams* [PhD, University College London].

Ward, K. M., Falade‐Nwulia, O., Moon, J., Sutcliffe, C. G., Brinkley, S., Haselhuhn, T., Katz, S., Herne, K., Arteaga, L., Mehta, S. H., Latkin, C., Brooner, R. K., & Sulkowski, M. S. (2019). A randomized controlled trial of cash incentives or peer support to increase HCV treatment

for persons with HIV who use drugs: The CHAMPS study. *Open Forum Infectious Diseases*, *6*(4), ofz166. https://doi.org/10.1093/ofid/ofz166

Yoon, J., Lo, J., Gehlert, E., Johnson, E. E., & O'Toole, T. P. (2017). Homeless veterans' use of peer mentors and effects on costs and

utilization in VA clinics. *Psychiatric Services*, *68*(6), 628–631. https://doi.org/10.1176/appi.ps.201600290

Andreae, S. J., Andreae, L. J., Richman, J. S., Cherrington, A. L., & Safford, M. M. (2021). Peer‐delivered cognitive behavioral therapybased

intervention reduced depression and stress in community dwelling adults with diabetes and chronic pain: A cluster randomized trial. *Annals of Behavioral Medicine*. *55*, 970-980, Online ahead of print. <https://doi.org/10.1093/abm/kaab034>

Chambers, S. K., Occhipinti, S., Stiller, A., Zajdlewicz, L., Nielsen, L., Wittman, D., Oliffe, J. L., Ralph, N., & Dunn, J. (2019).

Five‐year outcomes from a randomised controlled trial of a couples‐based intervention for men with localised prostate cancer. *Psycho‐Oncology*, *28*(4), 775–783. https://doi.org/10.1002/pon.5019

Cherrington, A. L., Khodneva, Y., Richman, J. S., Andreae, S. J., Gamboa, C., & Safford, M. M. (2018). Impact of peer support on acute care visits and hospitalizations for individuals with diabetes and depressive symptoms: A cluster‐randomized controlled trial. *Diabetes Care*,

*41*(12), 2463–2470. <https://doi.org/10.2337/dc18-0550>

Corrigan, P. W., Pickett, S., Schmidt, A., Stellon, E., Hantke, E., & Kraus, D. (2017). Peer navigators to promote engagement of homeless African Americans with serious mental illness in primary care. *Psychiatry* *Research*, *255*, 101–103. https://doi.org/10.1016/j.psychres.2017.

05.020

Easter, M. M., Swanson, J. W., Robertson, A. G., Moser, L. L., & Swartz, M. S. (2017). Facilitation of psychiatric advance directives

by peers and clinicians on assertive community treatment teams. *Psychiatric Services*, *68*(7), 717–723. https://doi.org/10.1176/appi.

ps.201600423

Hundert, C. G., Hareli, M., & Conley, C. S. (2021). Honest, open, proud—college: Follow‐up effects of a peer‐led group for reducing the

stigma of mental illness. *Stigma and Health*, *7*, 122-125. <https://doi.org/10.1037/sah0000326>

Khodneva, Y., Safford, M. M., Richman, J., Gamboa, C., Andreae, S., & Cherrington, A. (2016). Volunteer peer support, diabetes, and

depressive symptoms: Results from the ENCOURAGE trial. *Journal of Clinical & Translational Endocrinology*, *4*, 38–44. https://doi.org/

10.1016/j.jcte.2016.04.002

Lara‐Cabrera, M. L., Salvesen, O., Nesset, M. B., De las Cuevas, C., Iversen, V. C., & Grawe, R. W. (2016). The effect of a brief

educational programme added to mental health treatment to improve patient activation: A randomized controlled trial in community mental health centres. *Patient Education & Counseling*, *99*(5), 760–768. https://doi.org/10.1016/j.pec.2015.11.028

Schutt, R. K., Schultz, M., Mitchell‐Miland, C., McCarthy, S., Chinman, M., & Ellison, M. (2021). Explaining service use and residential stability in supported housing: Problems, preferences, peers. *Medical Care*, *59*(Suppl. 2), S117–S123. https://doi.org/10.1097/MLR.0000000000001498

Corrigan, P., Sheehan, L., Morris, S., Larson, J. E., Torres, A., Lara, J. L., Paniagua, D., Mayes, J. I., & Doig, S. (2018). The impact of a peer

navigator program in addressing the health needs of Latinos with serious mental illness": Correction. *Psychiatric Services*, *69*(7),

818–461.

Gassaway, J. (2017). Correction: Effects of peer mentoring on selfefficacy and hospital readmission after inpatient rehabilitation of

individuals with spinal cord injury: A randomized controlled trial (A*rchives of Physical Medicine and Rehabilitation* (2017) 98 (1526‐

1534.e2), (S0003999317301648), (10.1016/j.apmr.2017.02.018)). *Archives of Physical Medicine & Rehabilitation*, *98*(11), 2345–2346.

<https://doi.org/10.1016/j.apmr.2017.08.466>

Campbell, C. (2014). *An economic evaluation of a peer support intervention for diabetes self‐management* [PhD, University of Alabama at

Birmingham].

Hodgkin, D., Brolin, M. F., Ritter, G. A., Torres, M. E., Merrick, E. L., Horgan, C. M., Hopwood, J. C., De Marco, N., & Gewirtz, A. (2019).

Cost savings from a navigator intervention for repeat detoxification clients. *Journal of Mental Health Policy and Economics*, *22*(1), 3–13.

Williams, E. M., Dismuke, C. L., Faith, T. D., Smalls, B. L., Brown, E., Oates, J. C., & Egede, L. E. (2019). Cost‐effectiveness of a peer

mentoring intervention to improve disease self‐management practices and self‐efficacy among African American women with systemic lupus erythematosus: analysis of the Peer Approaches to Lupus Self‐management (PALS) pilot study. *Lupus*, *28*(8), 937–944. <https://doi.org/10.1177/0961203319851559>

Wingate, L., Graffy, J., Holman, D., & Simmons, D. (2017). Can peer support be cost saving? An economic evaluation of RAPSID: A

randomized controlled trial of peer support in diabetes compared to usual care alone in East of England communities. *BMJ Open Diabetes*

*Research & Care*, *5*(1), e000328. https://doi.org/10.1136/bmjdrc-2016-000328

Yu, D., Cai, Y., Graffy, J., Holman, D., Zhao, Z., & Simmons, D. (2021). Association between systolic blood pressure and cardiovascular

inpatient cost moderated by peer‐support intervention among adult patients with type 2 diabetes: A 2‐cohort study. *Canadian* *Journal of Diabetes*, *45*(2), 179–185. https://doi.org/10.1016/j.jcjd.2020.07.008
